# Gene expression profiling distinguishes prefibrotic from overtly fibrotic myeloproliferative neoplasms and identifies disease subsets with distinct inflammatory signatures

**DOI:** 10.1371/journal.pone.0216810

**Published:** 2019-05-09

**Authors:** Waihay J. Wong, Michele Baltay, Annaliese Getz, Kit Fuhrman, Jon C. Aster, Robert P. Hasserjian, Olga Pozdnyakova

**Affiliations:** 1 Department of Pathology, Brigham and Women’s Hospital, Harvard Medical School, Boston, Massachusetts, United States of America; 2 Nanostring Technologies Inc., Seattle, Washington, United States of America; 3 Department of Pathology, Massachusetts General Hospital, Harvard Medical School, Boston, Massachusetts, United States of America; Emory University, UNITED STATES

## Abstract

The Philadelphia chromosome-negative myeloproliferative neoplasms (MPN) share similar molecular characteristics in that they frequently harbor hotspot mutations in *JAK2*, *CALR* or *MPL*, leading to activated JAK/STAT signaling. However, these MPN have distinct symptoms, morphology, and natural histories, including different tendencies to progress to fibrosis. Although the role of inflammation in tissue fibrosis is well recognized, inflammatory gene expression in bone marrows involved by MPN has been understudied. We analyzed the expression of inflammatory genes by directly measuring RNA transcript abundance in bone marrow biopsies of 108 MPN patients. Unsupervised analyses identified gene expression patterns that distinguish prefibrotic (grade 1–2) MPN from overtly fibrotic (grade 2–3) MPN with high sensitivity and specificity in two independent cohorts. Furthermore, prefibrotic and overtly fibrotic MPN are separable into subsets with different activities in biological pathways linked to inflammation, including cytokines, chemokines, interferon response, and toll-like receptor signaling. In conclusion, this study demonstrates that MPN with overt fibrosis is associated with significant inflammatory gene upregulation in the bone marrow, revealing potential roles for multiple pro-inflammatory signaling networks in the development of myelofibrosis and suggesting potential therapeutic mechanisms to alleviate this process in the bone marrow microenvironment.

## Introduction

Essential thrombocythemia (ET), polycythemia vera (PV), and primary myelofibrosis (PMF) are a group of Philadelphia (Ph)-negative myeloproliferative neoplasms (MPN) characterized by overlapping clinical and laboratory features, as well as common phenotypic driver mutations in the *JAK2*, *CALR* and *MPL* tyrosine kinase genes. Despite these similarities, disease progression and clinical outcome vary greatly between disease types. For example, 12% of prefibrotic PMF patients develop overtly fibrotic disease within 10 years of diagnosis, whereas post-ET myelofibrosis is rarer, occurring in only 0.8% of ET patients [1]. The biological basis of bone marrow fibrosis in MPN remains unclear, but likely involves aberrant growth factor and cytokine signaling in neoplastic hematopoietic cells [2]. These proinflammatory molecules, such as transforming growth factor beta (TGF-β), platelet derived growth factor (PDGF), and fibroblast growth factor (FGF), elicit a secondary response in stromal fibroblasts and endothelial cells resulting in bone marrow fibrosis. However, the study of cytokine gene expression levels in the microenvironment has been technically difficult, and has sometimes produced contradictory findings [3].

We previously demonstrated that the mutational profiles of PMF, ET, and PV correlate with histomorphologic characteristics in a cohort of Ph-negative MPN patients [4]. In this study, we expand this cohort of PMF, ET, and PV patients to characterize levels of inflammatory gene expression in the bone marrow. Using a technique that permits direct measurement of transcript levels in clinical bone marrow biopsies, we demonstrate a strong correlation between myelofibrosis and inflammatory gene expression in the bone marrow. Gene expression profiles were identified that distinguish prefibrotic MPN from overtly fibrotic MPN and define MPN subsets with different inflammatory pathway activities. These results emphasize the central role of the inflammatory microenvironment in the initiation and persistence of myelofibrosis and suggest that distinct MPN phenotypes may be functionally categorized by differences in proinflammatory signals.

## Materials and methods

### Study population

The pathology archives at Brigham & Women’s Hospital (BWH) and Massachusetts General Hospital (MGH) was queried to identify patients diagnosed with PMF, ET, PV, or MPN, unclassifiable (MPN-U) on bone marrow biopsy with concurrent hematologic data obtained between 2005 and 2016. Patients diagnosed with myelodysplastic syndrome/MPN overlap disease and those who had progressed to acute leukemia, received treatment with chemotherapeutic agents for prior cancer diagnoses, or had undergone stem cell transplantation were excluded. BWH specimens were fixed in Bouin’s fixative and decalcified in RapidCal Immuno (BBC Biochemical) for 15 minutes, followed by routine processing. Specimens from MGH were fixed in B-plus fixative for a minimum of 4 hours and decalcified in RapidCal Immuno (BBC Biochemical) for 30 minutes, followed by routine processing. Histologic review and fibrosis grading were performed by W.W., R.P.H. and O.P. based on consensus, using the 2016 WHO Revised Classification of Myeloid Neoplasms [5]. The study was conducted in accordance with the principles set forth by the Declaration of Helsinki and the requirement for informed consent was waived by the institutional review board.

### Mutational analysis

Targeted sequencing of 95 commonly mutated genes in myeloid neoplasms was performed on DNA isolated from peripheral blood or bone marrow aspirates of 83 of the patients as part of their clinical evaluation. Amplicon library generation (TruSeq Custom Amplicon, Illumina, San Diego, CA) and next generation sequencing (MiSeq, Illumina, San Diego, CA) were performed as described [6]. Data processing and analysis were performed using MuTect for single-nucleotide variants with subsequent manual review and annotation (including evaluation of allele frequencies). Likely pathogenic variants were defined as frameshift, nonsense, splice-site mutations, insertions-deletions, or known pathogenic missense alterations.

### Gene expression analysis

RNA was isolated from 50 μm sections prepared from fixed, decalcified and paraffin-embedded bone marrow biopsies using Qiagen RNeasy Kit (Germantown, MD). Briefly, 50 μm histologic sections were scraped into deparaffinization solution and RNA was isolated according to manufacturer’s instructions. RNA concentration was measured using a Nanodrop spectrophotometer (Thermo Fisher, Waltham, MA). Multiplexed mRNA quantification was performed using Nanostring nCounter GX Human Inflammation Kit (Seattle, WA), which contains color-coded hybridization probes against 249 inflammation-related genes, including cytokines, chemokines, pattern recognition receptors, cell adhesion molecules, and regulators of lymphocyte activation. Gene expression analysis was carried out using nSolver software (Nanostring, Seattle, WA) and RStudio (Boston, MA). For each gene, transcript count was normalized to the geometric mean of five housekeeping genes (*GAPDH*, *GUSB*, *HPRT1*, *PGK1*, *TUBB*) and six synthetic positive control RNA probes. Baseline threshold expression was defined as two standard deviations above the mean of six synthetic negative control RNA probes. Target genes with raw counts below the baseline threshold in more than two-thirds (67%) of samples were excluded from analysis. Samples with more than 75% of raw transcript counts below the baseline threshold, or requiring a housekeeping gene normalization factor of greater than 20.0, were considered to be of inadequate quality and excluded from analysis.

### Statistical analysis

Cluster analysis was performed using *stats* R package. The k coefficient for k-means clustering was determined by the elbow method. Stepwise selection of differentially expressed genes was performed using the stepAIC function in *MASS*. Logistic regression modeling and leave one out cross validation were performed using *caret*. The predictive score cut-off was determined by recursive partitioning using *rpart* R package. Gene set enrichment analysis was performed using GSEA software and Molecular Signature Database (Broad Institute, Boston, MA). Differentially expressed genes were also analyzed for enrichment in Gene Ontology pathways (http://www.geneontology.org/page/go-enrichment-analysis, accessed August 2018). Statistical significance (p < 0.05) was determined using log_2_ transformed values by one-way ANOVA or Student’s t test, as appropriate. Adjusted false discovery rate (FDR; q < 0.05) was calculated using the Benjamini-Yekutieli method.

## Results

We identified 142 bone marrow biopsies from unique individuals diagnosed with MPN. 115 samples were from BWH; 27 samples were from MGH; tissue for gene expression analyses was available in 135 specimens. Of 135 specimens with available tissue, 108 (80.0%) yielded gene expression profiles of adequate quality and constituted the final study sample. Low quality samples excluded from the analysis were associated with older specimen age and fixation in B-Plus fixative. There was no significant association between sample exclusion and MPN disease type (p = 0.9) or fibrosis grade (p = 0.9). The final cohort of 108 MPN patients included 36 (33.3%) with PMF, 31 (28.7%) with ET, 25 (23.1%) with PV, and 16 (14.8%) with MPN-U (Table 1). Of the 36 PMF cases, 13 (34.2%) were prefibrotic (WHO MF grade 0–1), whereas 23 (65.8%) showed overt fibrosis (WHO MF grade 2–3). Eight cases (32%) of PV had progressed to post-PV myelofibrosis and 10 (32.2%) cases of ET had progressed to post-ET myelofibrosis at the time of the analyzed biopsy. Seven (43.8%) out of 16 MPN-U cases represented advanced stages of MPN in which fibrosis obscured the underlying diagnosis. In total, 60 cases (55.6%) represented MPN with WHO MF grade 0–1, whereas 48 cases (44.4%) demonstrated overt (WHO MF grade 2–3) fibrosis. These specimens were randomly divided into training (n = 76) and test (n = 32) cohorts for further analysis (Table 1).

**Table 1:** MPN patients’ characteristics.

	All patients (n = 108)				Training cohort (n = 76)				Test cohort (n = 32)			
	PMF	ET	PV	MPN-U	PMF	ET	PV	MPN-U	PMF	ET	PV	MPN-U
	(n = 36)	(n = 31)	(n = 25)	(n = 16)	(n = 26)	(n = 21)	(n = 17)	(n = 12)	(n = 10)	(n = 10)	(n = 8)	(n = 4)
Characteristics												
Mean age	65.4	60	60.8	62.4	65.2	57	61.4	63	65.8	64	61	60.3
Female (%)	14 (40)	18 (62)	14 (61)	8 (50)	9 (36)	11 (58)	8 (57)	7 (58)	5 (50)	6 (67)	5 (63)	1 (25)
Fibrosis												
Grade 0–1 (%)	13 (36)	21 (68)	17 (68)	9 (56)	7 (27)	14 (67)	9 (53)	7 (58)	6 (60)	7 (70)	8 (100)	2 (50)
Grade 2–3 (%)	23 (64)	10 (32)	8 (32)	7 (44)	19 (73)	7 (33)	8 (47)	5 (42)	4 (40)	3 (30)	0 (0)	2 (50)
Phenotypic driver mutation												
JAK2 (%)	19 (53)	12 (39)	20 (80)	11 (69)	16 (62)	10 (48)	14 (82)	9 (75)	3 (30)	2 (20)	6 (75)	2 (50)
CALR (%)	3 (8)	8 (26)	0 (0)	2 (13)	2 (8)	5 (24)	0	2 (17)	1 (10)	3 (30)	0	0
MPL (%)	4 (11)	1 (3)	0 (0)	0	3 (12)	1 (5)	0	0	1 (10)	0	0	0
No JAK2, CALR, or MPL (%)	4 (11)	1 (3)	2 (8)	2 (13)	0	0	0	1 (8)	4 (40)	1 (10)	2 (25)	1 (25)
Not assessed (%)	6 (17)	9 (29)	3 (12)	1 (6)	5 (19)	5 (24)	3 (18)	0	1 (10)	4 (40)	0	1 (25)
Treatment												
JAK inhibitor (prior)	5 (14)	0	2* (8)	0	4 (15)	0	2* (12)	0	1 (10)	0	0	0
JAK inhibitor (current)	3 (8)	1 (3)	2 (8)	0	3 (12)	1 (5)	2 (12)	0	0	0	0	0
Interferon (prior)	0	0	1* (4)	0	0	0	1* (6)	0	0	0	0	0
Interferon (current)	0	1 (3)	2 (8)	0	0	1 (5)	1 (6)	0	0	0	1 (13)	0

* 1 patient previously treated with both JAK2 inhibitor and interferon

**Fig 1:**
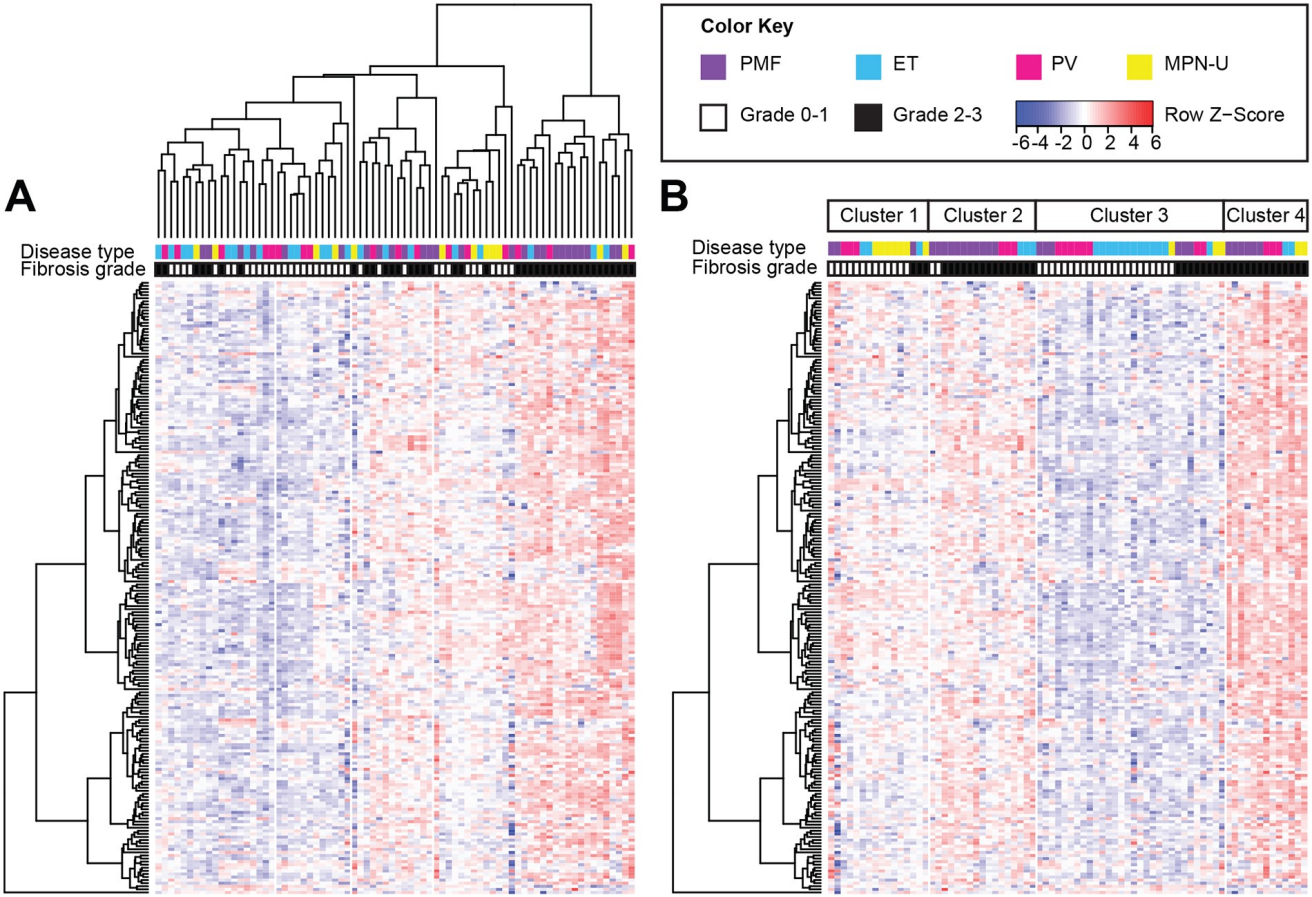
Cluster analysis of inflammatory gene expression in MPN patients. (A) Hierarchical clustering separates MF grade 0–1 MPN from MF grade 2–3 MPN in a training cohort of 76 patients. (B) K-means clustering (k = 4) separates prefibrotic (MF grade 0–1) disease from overtly fibrotic disease (MF grade 2–3). Four clusters with differential gene expression are seen: clusters 1 and 3 consist of mostly prefibrotic MPN, whereas clusters 2 and 4 consist of overtly fibrotic MPN. Each row represents a single gene (n = 199). Each column represents 1 of 76 patients. The relative abundance (log_2_ count) of each gene transcript is indicated by the color bar.

Unsupervised clustering of inflammatory gene expression was performed on the training cohort using both hierarchical and agglomerative methods. Hierarchical clustering separated overtly fibrotic MPN from prefibrotic disease with a sensitivity of 72% and a specificity of 89% (Fig 1A). However, this analysis did not distinguish specific MPN disease types (ET, PV, prefibrotic PMF, versus MPN-U with MF grade 0–1 in the prefibrotic cluster; and post-ET MF, post-PV MF, overtly fibrotic PMF, and MPN-U with MF grade 2–3 in the overtly fibrotic cluster) from each other. K-means agglomerative clustering (k = 4) demonstrated that both prefibrotic and overtly fibrotic MPN can be further partitioned into two groups, each with distinct gene expression patterns (Fig 1B). Cluster 1 and cluster 3 represented mostly prefibrotic MPN, whereas clusters 2 and 4 contained mostly fibrotic disease. The overall sensitivity was 95% for prefibrotic MPN and 72% for overtly fibrotic disease respectively, with an overall accuracy of 83%. Cluster 1 contained most of MF grade 0–1 MPN-U; only three cases (30%) were present in cluster 3, which was relatively enriched in ET instead (Fig 1B). In contrast, overtly fibrotic PMF was concentrated in cluster 2, whereas cluster 4 showed no disease-specific patterns. These differences in disease type were not statistically significant (p > 0.05), suggesting that in this analysis, pro-inflammatory responses represent a final common pathway leading to end-stage fibrosis in MPN. Therefore, we focused on inflammatory genes with altered expression in myelofibrosis independent of disease type.

Out of 199 genes expressed above background, the transcript levels of 123 genes were significantly altered in the bone marrow of patients with overt fibrosis (q < 0.05; S1 Table). Only three transcripts (*HMGB2*, *ALOX15*, *DEFA1*) were downregulated, while the remainder were upregulated. Five genes (*CCL2*, *MX1*, *IFIT1*, *CXCL10*, *C1R*) were upregulated by greater than 3-fold; 28 genes were upregulated by 2- to 3-fold (S1 Table). Notably, *CCL2* is upregulated in other disorders marked by abnormal fibrosis, including fibrosing diseases of the lung, liver, and kidney. Other upregulated transcripts include additional mediators of the fibrogenic response [7], such as *TGFB3* (2.6-fold upregulated), *TGFB1* (1.9-fold upregulated), *PDGFA* (2.2-fold upregulated), and *HIF1A* (1.3-fold upregulated). Cytokines implicated in tissue fibrosis, such as *TNF* (1.9-fold upregulated) and *IL1B* (1.5-fold upregulated), were also significantly upregulated. Other upregulated transcripts encode toll-like receptors and their downstream effectors, such as *TLR3*, *TLR5*, *RIPK2*, and *IRF7*. In addition, some of the most highly upregulated genes in overtly fibrotic MPN included downstream targets of interferon signaling (*IFIT1* and *MX1*; both upregulated 3.5-fold).

**Fig 2:**
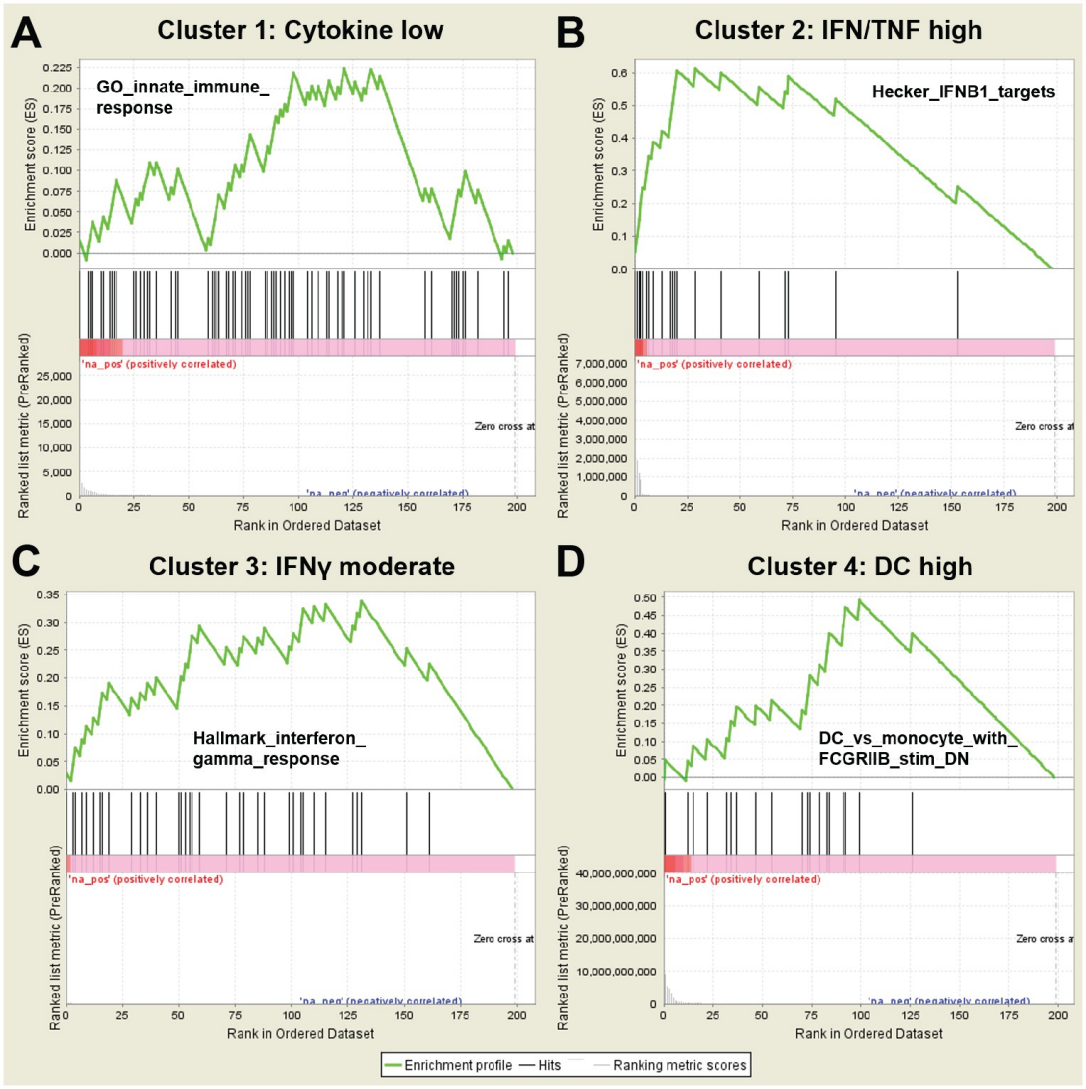
Inflammatory gene expression signatures in each MPN cluster. For each MPN subgroup identified by cluster analysis, an example of a positively or negatively correlated gene set from the Molecular Signature Database is shown. (A) Cluster 1 shows no significant enrichment in genes involved in innate immunity and other inflammatory pathways (q = 0.98). (B) Gene expression in cluster 2 is enriched for IFNB1 and TNF signaling (q < 0.05). (C) Cluster 3 shows moderate enrichment in interferon-γ pathway genes (q < 0.05). (D) Cluster 4 shows enrichment by genes associated with mature dendritic cells (q < 0.05).

Gene set enrichment analysis (GSEA) confirmed that each MPN cluster is enriched in genes associated with distinct expression modules (Table 2 and Fig 2). Cluster 1 showed no gene expression profile enrichment by GSEA and is termed “cytokine low”. Cluster 3 showed moderately increased transcript levels of genes involved in cytokine signaling, including a significant enrichment in interferon gamma gene expression signature (“IFNγ moderate”). Compared to clusters 1 and 3, clusters 2 and 4 were characterized by high inflammatory cytokine gene expression. Cluster 2 showed abundant expression of interferons and tumor necrosis factors (“IFN/TNF high”), whereas cluster 4 demonstrated a gene expression signature associated with mature dendritic cells (“DC high”). Interestingly, although clusters 2, 3 and 4 demonstrated increased expression of PPARG2 associated genes, only cluster 2 (“IFN/TNF high”) showed increased expression of PPARG1 pathway genes, indicating a differential contribution of these closely-related inflammatory mediators towards certain types of myelofibrosis. We further carried out gene ontology (GO) term enrichment analysis (S2 Table), which identified pathways similar to those highlighted by GSEA. These pathways included JAK/STAT signaling, Toll-like receptor signaling, NFκB signaling, TNF signaling, interferon signaling, cytokine production and chemotaxis. In addition, pathways involved in VEGF signaling and osteoclast differentiation were significantly enriched in MPN demonstrating overt fibrosis (MF grade 2–3).

**Table 2:** Gene set enrichment analysis of MPN subgroups identified by cluster analysis.

	Cluster 1		Cluster 2		Cluster 3		Cluster 4	
Fibrosis grade	(Cytokine low)		(IFN/TNF high)		(IFNγ moderate)		(DC high)	
MF grade 0–1 (%)	16 (84%)		2 (12%)		22 (73%)		0 (0%)	
MF grade 2–3 (5)	3 (16%)		15 (88%)		8 (27%)		13 (100%)	
	NES*	FDR*	NES*	FDR*	NES*	FDR*	NES*	FDR*
Cytokine signaling								
Hallmark_Interferon_Gamma_Response	-	-	2.47	0.003	2.18	0.12	2.77	0.01
Hecker_IFNB1_Targets	1.1	1	3.14	6.00E-04	1.67	0.35	1.7	0.29
Reactome_Interferon _Signaling	0.62	1	2.65	0.001	1.04	0.81	1.28	0.6
Sana_TNF_Signaling_Up	1.04	1	2.69	9.00E-04	1.54	0.43	1.46	0.46
Peroxisome activity								
GSE37533_PPARG1 transduced CD4 T cells	0.76	1	3.02	3.00E-04	1.93	0.27	2.08	0.11
GSE37533_PPARG2 transduced CD4 T cells	0.7	1	3.01	3.00E-04	2.49	0.09	2.46	0.03
Cell-type specific patterns								
GSE26030_Th1 vs Th17 stimulated CD4 T cells day 5	1.02	1	2.47	0.003	1.82	0.33	2.23	0.09
GSE7509_FcgR-mediated monocyte and dendritic cell maturation	0.84	1	2.33	0.007	2.25	0.09	2.47	0.04

*NES, normalized enrichment score; FDR, false discovery rate.

Only a few genes showed a significant correlation with distinct driver mutations. *TGFB2* was significantly upregulated 3.1-fold in *MPL*-mutant disease, whereas *PTGER3* was downregulated 2.2-fold in MPN lacking *JAK2*, *MPL*, or *CALR* mutations (‘triple negative’ disease). The number of genes that are differentially expressed in various MPN disease types were small and no statistically significant pathway enrichment was detected.

**Fig 3:**
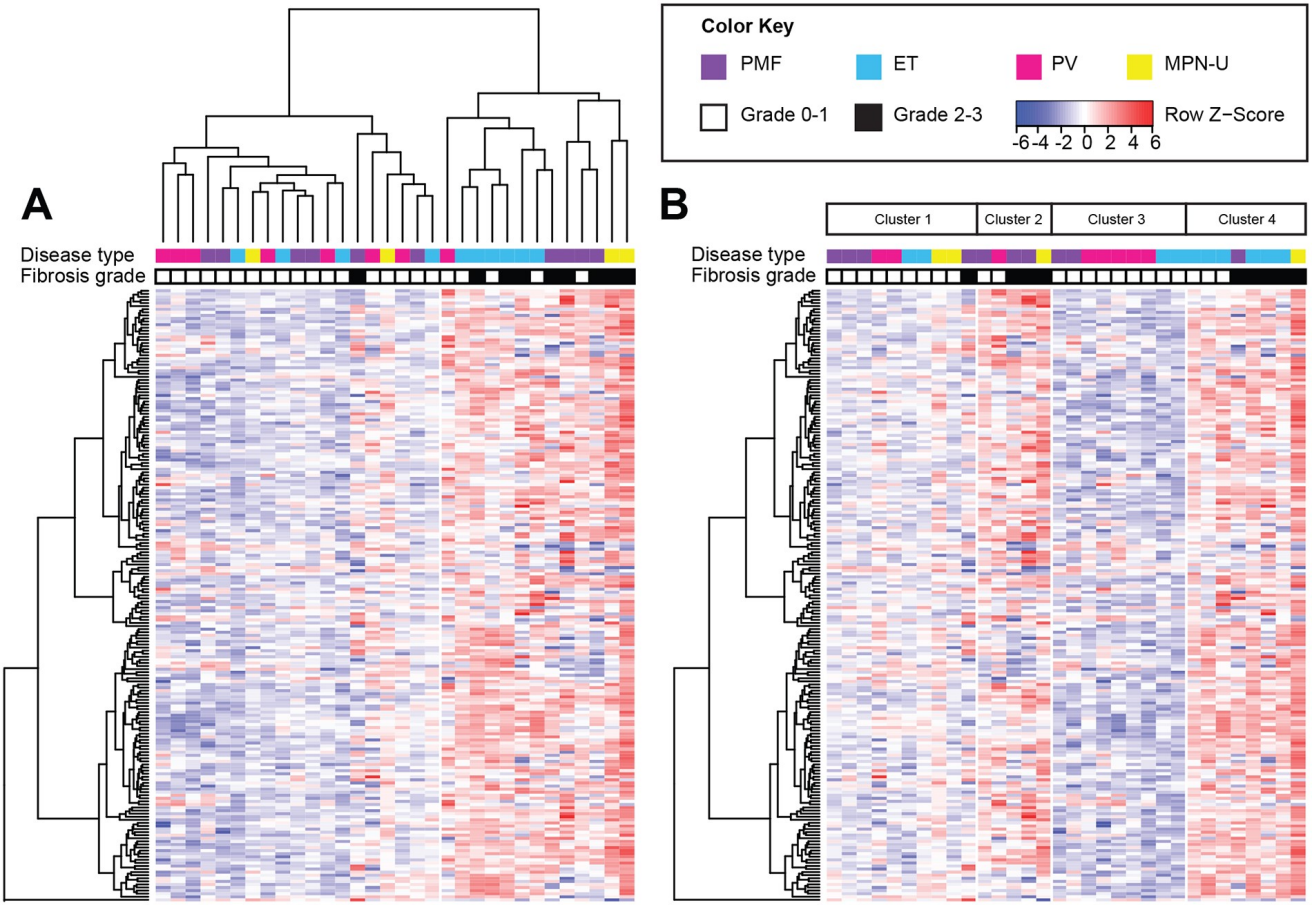
Independent validation of MPN subgroups by cluster analysis. (A) Hierarchical clustering separates MF grade 0–1 MPN from MF grade 2–3 MPN in a test cohort of 32 patients. (B) K-means clustering (k = 4) separates prefibrotic (MF grade 0–1) disease from overtly fibrotic disease (MF grade 2–3) into four clusters. Clusters 1 and 3 consist of mostly prefibrotic MPN, whereas clusters 2 and 4 consist of overtly fibrotic MPN. Each row represents a single gene (n = 199). Each column represents 1 of 32 patients. The relative abundance (log_2_ count) of each gene transcript is indicated by the color bar.

An independent validation cohort demonstrated similar changes in gene expression between non-fibrotic and fibrotic MPN. Similar to the training cohort, hierarchical and k-means clustering of differentially expressed genes in the test cohort segregated overtly fibrotic (grade 2–3) disease from MPN with grade 0–1 fibrosis, with a sensitivity of 89% and specificity of 78% in both clustering algorithms (Fig 3A and 3B). We constructed a generalized logistic regression model using stepwise selection to identify the most distinctive gene expression patterns between non-fibrotic and fibrotic MPN. This predictive signature consists of five differentially expressed genes in fibrotic MPN: *DDIT3*, *ALOX15*, *TCF4*, *MAPK14*, and *MAPKAPK5*. Using this model, we accurately predicted bone marrow fibrosis in the test cohort with a sensitivity of 78% and a specificity of 91%, with an overall accuracy of 88% (kappa = 0.69). We also performed leave-one-out cross validation in a combined dataset comprising both training and test cohorts, which generated an overall prediction accuracy of 86% (kappa = 0.72) in assigning fibrosis grade.

**Fig 4:**
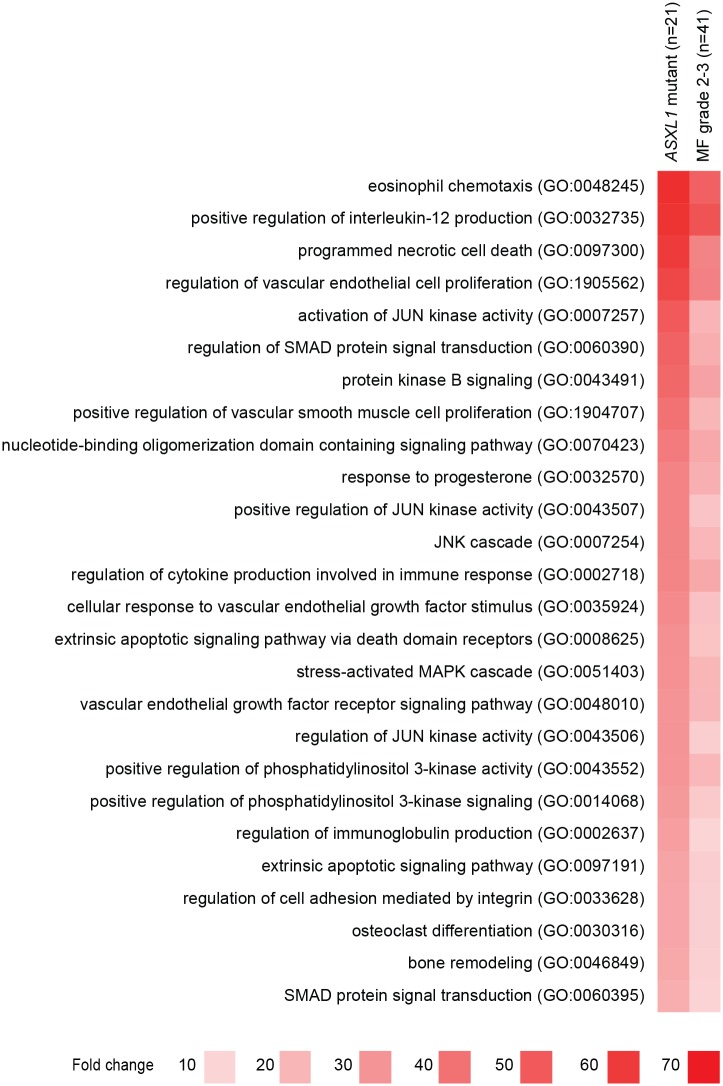
Gene ontology (GO) term enrichment in *ASXL1* mutant MPN and overtly fibrotic (MF grade 2–3) MPN. Select GO terms are shown that demonstrate greater fold enrichment in *ASXL1* mutant MPN compared to MPN with grade 2–3 fibrosis. The color intensity reflects absolute fold enrichment. q < 0.05 for all data shown.

We and others have reported that overtly fibrotic PMF is enriched in *ASXL1* mutations [4,8]. In 83 cases from both training and test cohorts with sequencing data, *ASXL1* mutation was present in 21 (25.3%) cases. The presence of *ASXL1* mutation correlated with the upregulation of 58 genes (S3 Table). All but five of these genes (*KEAP1*, *DAXX*, *MAPKAPK5*, *TLR9*, *RIPK1*; 8.6%) belong to the larger set of 123 differentially expressed genes in overtly fibrotic MPN (S1 Table). GO term enrichment analysis of these 58 genes demonstrates that the overrepresented biological processes in *ASXL1* mutant disease are similar to those observed in overtly fibrotic MPN (S4 Table). However, multiple growth factor pathways, such as MAPK/JNK, PI3K/AKT, and VEGF signaling show comparatively greater enrichment in *ASXL1* mutant disease than in overtly fibrotic MPN, suggesting that the lack of *ASXL1* specifically enhances receptor tyrosine kinase signaling in MPN (Fig 4).

Many of the therapies given to MPN patients, such as JAK inhibitors or interferon, would be expected to impact inflammatory gene expression. 16 out of 108 patients (14.8%) had prior (n = 8) or current (n = 8) exposure to JAK inhibitor or interferon (Table 1). In a comparison with 38 control patients matched for fibrosis grade, no differentially regulated genes were identified (data not shown).

## Discussion

The study of MPN is challenging due to the wide degree of overlap in mutational landscape, histologic features, and clinical presentation [9]. Furthermore, the development of myelofibrosis is a shared characteristic in all MPN, albeit with varying frequencies depending on the disease type. Our results suggest that overt myelofibrosis coincides with inflammatory gene upregulation in the bone marrow of MPN patients. The inflammatory gene expression pattern differs in various subsets of prefibrotic and overtly fibrotic MPN, suggesting that distinct inflammatory pathways give rise to the common phenotype of bone marrow fibrosis in advanced MPN. These findings suggest a new taxonomy of MPN based on transcriptional patterns in inflammation-related pathways. These gene expression profiles do not correlate with MPN disease type. Instead, the high degree of overlap between differentially regulated transcripts in *JAK*, *MPL* and *CALR*-mutant MPN supports the concept that MPN pathogenesis converges on a common JAK/STAT axis [2].

The broadest distinction in inflammatory gene expression lies between MF grade 0–1 (prefibrotic) and MF grade 2–3 (overtly fibrotic) MPN (Fig 1). Prefibrotic MPN is characterized by overall low cytokine gene expression. In contrast, overtly fibrotic MPN activate multiple inflammatory pathways. These pathways include Toll-like receptor (TLR) signaling and its downstream components--including NF kappa-B, MAPK, and TNF signaling (Table 2). TLR signaling in fibroblasts and tissue macrophages has been implicated in hepatic, cardiac and pulmonary fibrosis [10]. Our findings suggest that sustained proinflammatory cues in the bone marrow similarly lead to TLR-mediated fibrosis. Furthermore, two of the downregulated transcripts identified in this study, *HMGB1* and *HMGB2*, are putative endogenous TLR ligands, suggesting that there is a negative modulatory response to abnormally activated TLR signaling in the microenvironment of overtly fibrotic MPN.

In contrast, other upregulated genes are likely to affect both the neoplastic myeloid clone as well as bone marrow inflammatory cells. For example, activated JAK/STAT signaling is a defining feature of clonal myelopoiesis in MPN, but JAK/STAT activity in stromal cells can also lead to fibroblast activation and myelofibrosis [11,12]. Another example is the IL1 family of cytokines, which exert a direct effect on myeloid development and are associated with fibrotic transformation in PV and PMF [13–15]. *IL1RAP*, a gene encoding the IL1 coreceptor, is upregulated in overtly fibrotic MPN and may represent a potential therapeutic target against myelofibrosis via anti-IL1RAP antibodies [16]. In a subset of MPN with grade 2–3 fibrosis, GSEA identified a gene expression pattern characteristic of dendritic cells ("DC high") (Table 2). Dendritic cells are a major source of pro-inflammatory cytokines in MPN and are efficiently targeted by ruxolitinib [17]. Dendritic cells also present antigens to T cells, priming them toward effector or regulatory responses. A recent study showed that defective CD4 T cell activation by dendritic cells causes a myeloproliferative phenotype in mice, highlighting the contribution of stromal inflammatory cells to MPN pathogenesis [18].

Importantly, the pattern of inflammatory gene expression did not generally differ between MPN disease types in our study, except in rare specific circumstances. MPN-U with MF grade 0–1 was absent from the “cytokine low” cluster and was instead enriched in the “IFNγ moderate” cluster, demonstrating that MPN-U in the prefibrotic phase possesses elevated pro-inflammatory activity compared to other prefibrotic MPN. Conversely, ET with MF grade 0–1 was relatively enriched in the “cytokine low” cluster, suggesting minimal inflammatory pathway activity compared to pre-fibrotic PMF, PV, and MPN-U. This gene expression profile may be related to the histological characteristics of ET, which displays relatively lower cellularity and lacks myeloid and/or erythroid hyperplasia in comparison to PV and prefibrotic PMF [5].

In overtly fibrotic MPN, increased marrow fibrosis in MPN is accompanied by an alteration in the bone marrow cellular composition. These histologic changes include increased stromal elements as well as myeloid and megakaryocytic hyperplasia [5,19]. Although our experiments do not provide spatial resolution of transcriptional changes within the bone marrow environment, we observed upregulation of genes important in myofibroblast proliferation in overtly fibrotic MPN, such as *PDGFA* and *MAFK* [20,21]. Furthermore, the upregulation of specific transcripts that are highly expressed in distinct hematopoietic cell lineages (e.g. *CD163* and *CD40LG*) suggests that inflammatory cell populations, including macrophages and T lymphocytes, are increased in the bone marrow of overtly fibrotic MPN. GO analysis also demonstrated enrichment of osteoclast differentiation and angiogenic pathway genes in overtly fibrotic MPN. Therefore, multiple cell types contribute to the proinflammatory nature of a fibrotic marrow [22,23]. Intriguingly, a subset of MPN with grade 0–1 fibrosis demonstrate increased interferon gamma gene expression signature (cluster 3), suggesting that increased cytokine activity in the bone marrow microenvironment predates the onset of overt fibrosis [23].

In conclusion, we have found that overtly fibrotic MPN demonstrates marked upregulation of inflammatory genes within the bone marrow via multiple proinflammatory signaling networks. Clustering analysis and GSEA identified four subsets of MPN that are distinguished from each other by fibrosis grade and inflammatory gene expression (chemokines, cytokines, and innate immune response pathways) in the bone marrow. These results were validated in an independent cohort. By using bone marrow biopsies obtained as part of the routine care of patients with MPN, we have demonstrated a robust method of gene expression profiling that is applicable to Bouin’s-fixed, decalcified tissue. Furthermore, the upregulation of transcripts encoding targetable proteins in overtly fibrotic MPN suggests potential therapeutic mechanisms to alleviate myelofibrosis in MPN.

## Supporting information

S1 Table Differentially expressed genes in MPN with Grade 0–1 and Grade 2–3 fibrosis. (PDF)

S2 Table Gene ontology terms enriched in MPN with Grade 2–3 fibrosis. (PDF)

S3 Table Differentially expressed genes in *ASXL*1 mutant MPN. (PDF)

S4 Table Gene ontology terms enriched in *ASXL1* mutant MPN. (PDF)
