# Supplementary material for: Gene expression profiling distinguishes prefibrotic from overtly fibrotic myeloproliferative neoplasms and identifies disease subsets with distinct inflammatory signatures
Source: PLoS One. 2019 May 9;14(5):e0216810. doi: 10.1371/journal.pone.0216810 (PMC6534080; doi:10.1371/journal.pone.0216810)
Supplement: S1 Table — (PDF) [file pone.0216810.s001.pdf]

Supplementary Table 1. Differentially expressed genes in MPN with Grade 0-1 and Grade 2-3 fibrosis

Gene	Accession #	Class Name	Fold change	FDR
TNFAIP3	NM_006290.2	Endogenous	2.129999067	6.2082E-09
DDIT3	NM_004083.4	Endogenous	2.104483946	6.2082E-09
MX1	NM_002462.2	Endogenous	3.533993519	3.5688E-08
C1R	NM_001733.4	Endogenous	3.090994517	3.5688E-08
SHC1	NM_001130040.1	Endogenous	1.569043336	3.5688E-08
TCF4	NM_003199.1	Endogenous	1.975306981	1.2511E-07
CCL2	NM_002982.3	Endogenous	3.395385874	1.3964E-07
HSPB2	NM_001541.3	Endogenous	2.612969175	2.0858E-07
LY96	NM_015364.2	Endogenous	1.806650973	2.896E-07
C1S	NM_001734.2	Endogenous	2.606172629	4.1598E-07
IFIT1	NM_001548.3	Endogenous	3.497665973	5.4064E-07
SMAD7	NM_005904.2	Endogenous	2.257726881	7.5652E-07
HMGB2	NM_001130688.1	Endogenous	0.642776145	1.2495E-06
OASL	NM_198213.1	Endogenous	2.92860034	1.2924E-06
IFI44	NM_006417.4	Endogenous	2.758543193	1.7796E-06
MX2	NM_002463.1	Endogenous	2.081002115	2.4459E-06
OAS2	NM_016817.2	Endogenous	2.426908513	2.996E-06
STAT2	NM_005419.2	Endogenous	1.617207648	2.996E-06
TGFB1	NM_000660.3	Endogenous	1.937097255	6.9696E-06
TNF	NM_000594.2	Endogenous	1.895574392	8.722E-06
STAT3	NM_139276.2	Endogenous	1.512288992	8.9064E-06
TGFB3	NM_003239.2	Endogenous	2.561091945	1.2014E-05
CSF1	NM_000757.4	Endogenous	1.923082084	1.3256E-05
MEF2A	NM_005587.2	Endogenous	1.59666381	1.3256E-05
RIPK2	NM_003821.5	Endogenous	1.524821735	1.4756E-05
CCL8	NM_005623.2	Endogenous	2.357413588	1.9285E-05
C1QA	NM_015991.2	Endogenous	1.860856973	2.1701E-05
ELK1	NM_005229.3	Endogenous	1.536265805	2.522E-05
TLR3	NM_003265.2	Endogenous	2.095692929	2.5634E-05
IFIT2	NM_001547.4	Endogenous	2.596036028	2.6599E-05
RELB	NM_006509.2	Endogenous	2.021232345	2.8544E-05
CD40	NM_001250.4	Endogenous	1.776408558	4.4343E-05
HIF1A	NM_001530.2	Endogenous	1.349577312	4.6691E-05
PDGFA	NM_002607.5	Endogenous	2.202880232	4.6786E-05
MRC1	NM_002438.2	Endogenous	1.957177687	4.7171E-05
HSPB1	NM_001540.3	Endogenous	1.773728724	4.7171E-05
PTK2	NM_005607.3	Endogenous	1.664132817	5.1456E-05
MAFK	NM_002360.3	Endogenous	1.442018928	6.0734E-05
IL1RAP	NM_002182.2	Endogenous	1.553972727	9.1511E-05
CXCL10	NM_001565.1	Endogenous	3.171947165	9.1756E-05
IFIT3	NM_001031683.2	Endogenous	2.106539507	9.6443E-05
GNAQ	NM_002072.2	Endogenous	1.301117432	9.6899E-05
MEF2C	NM_002397.3	Endogenous	1.824870247	0.00010948

RHOA	NM_001664.2	Endogenous	1.285537606	0.00011339
TBXA2R	NM_001060.3	Endogenous	1.831832705	0.00013027
C1QB	NM_000491.3	Endogenous	1.704184699	0.00013027
ROCK2	NM_004850.3	Endogenous	1.37026072	0.00013027
C7	NM_000587.2	Endogenous	2.012346498	0.0001567
CCL13	NM_005408.2	Endogenous	2.492474283	0.00023893
IRF7	NM_001572.3	Endogenous	1.796105225	0.00027417
FLT1	NM_002019.4	Endogenous	1.696917629	0.00031402
C2	NM_000063.3	Endogenous	1.64820099	0.00033068
STAT1	NM_007315.2	Endogenous	1.726405901	0.00034258
IL1R1	NM_000877.2	Endogenous	1.572517489	0.00034258
MAPKAPK2	NM_004759.3	Endogenous	1.277385993	0.00034258
MAPK3	NM_001040056.1	Endogenous	1.361752826	0.00046768
TGFBR1	NM_004612.2	Endogenous	1.44330935	0.00048074
PTGS1	NM_000962.2	Endogenous	1.914615481	0.00050663
CFB	NM_001710.5	Endogenous	2.397945436	0.00054187
CCR1	NM_001295.2	Endogenous	1.568499542	0.00054187
HRAS	NM_005343.2	Endogenous	1.428130861	0.00056767
GNAS	NM_080425.1	Endogenous	1.365855806	0.00066499
ALOX15	NM_001140.3	Endogenous	0.522652094	0.00066499
CD40LG	NM_000074.2	Endogenous	1.75631659	0.0006693
MEF2D	NM_005920.2	Endogenous	1.540167107	0.00067005
RELA	NM_021975.2	Endogenous	1.332421157	0.00075887
PTGFR	NM_000959.3	Endogenous	2.091708011	0.00077742
RAC1	NM_198829.1	Endogenous	1.384779261	0.00082962
CXCL9	NM_002416.1	Endogenous	2.379156859	0.00101721
PRKCA	NM_002737.2	Endogenous	1.657401533	0.00103533
HLA-DRB1	NM_002124.1	Endogenous	2.173569769	0.0010706
PTGER3	NM_000957.2	Endogenous	1.638175354	0.0012236
PLCB1	NM_182734.1	Endogenous	1.47841802	0.0012236
CXCL3	NM_002090.2	Endogenous	2.196873336	0.00127739
TRADD	NM_003789.2	Endogenous	1.464813007	0.00129595
PTGIR	NM_000960.3	Endogenous	1.891587812	0.00134484
MAP3K5	NM_005923.3	Endogenous	1.334899356	0.00147979
CD163	NM_004244.4	Endogenous	1.610332172	0.00184802
MAPK8	NM_002750.2	Endogenous	1.254213384	0.00189374
TGFB2	NM_003238.2	Endogenous	2.020261327	0.00191898
PRKCB	NM_212535.1	Endogenous	1.368809615	0.00191898
CCR4	NM_005508.4	Endogenous	1.740139839	0.00194911
IL8	NM_000584.2	Endogenous	2.258287601	0.00206343
ATF2	NM_001880.2	Endogenous	1.245608753	0.00222105
LIMK1	NM_002314.3	Endogenous	1.475323081	0.00237043
CCL4	NM_002984.2	Endogenous	1.862651209	0.00263075
IRF1	NM_002198.1	Endogenous	1.384089672	0.00267518
MKNK1	NM_003684.3	Endogenous	1.286155696	0.00285861
CCL5	NM_002985.2	Endogenous	1.90220565	0.00295435
FASLG	NM_000639.1	Endogenous	1.901148393	0.00295435

TLR5	NM_003268.3	Endogenous	1.635351618	0.00295435
IL15	NM_000585.3	Endogenous	1.576259144	0.00295435
JUN	NM_002228.3	Endogenous	2.128919799	0.0033369
MYD88	NM_002468.3	Endogenous	1.301492064	0.00381957
NFE2L2	NM_006164.3	Endogenous	1.279468464	0.00381957
PTGER4	NM_000958.2	Endogenous	1.384638625	0.00391729
C3AR1	NM_004054.2	Endogenous	1.380192737	0.00404776
HLA-DRA	NM_019111.3	Endogenous	1.64746195	0.00452942
NOD1	NM_006092.1	Endogenous	1.367882236	0.00498751
CXCL5	NM_002994.3	Endogenous	2.009064417	0.00666898
MAX	NM_002382.3	Endogenous	1.34231673	0.0069336
CXCL2	NM_002089.3	Endogenous	1.802784633	0.00788429
C5	NM_001735.2	Endogenous	1.474010324	0.00788429
MAFG	NM_002359.2	Endogenous	1.259261378	0.00803648
MAP2K4	NM_003010.2	Endogenous	1.229983811	0.00893064
TLR4	NM_138554.2	Endogenous	1.369536711	0.00933644
CCL19	NM_006274.2	Endogenous	2.207699597	0.01232559
DEFA1	NM_004084.2	Endogenous	0.593920764	0.01500716
TWIST2	NM_057179.2	Endogenous	1.636145141	0.01749724
CD4	NM_000616.3	Endogenous	1.375515328	0.01864556
GNB1	NM_002074.3	Endogenous	1.263303868	0.0188964
FXYD2	NM_021603.3	Endogenous	1.693451714	0.01931326
CYSLTR2	NM_020377.2	Endogenous	1.552313398	0.01985236
TLR2	NM_003264.3	Endogenous	1.284582659	0.02202625
NR3C1	NM_001018074.1	Endogenous	1.204366837	0.02267203
LTA	NM_000595.2	Endogenous	1.690867878	0.0272804
IL1B	NM_000576.2	Endogenous	1.517199167	0.03053916
NFKB1	NM_003998.2	Endogenous	1.218289907	0.0308445
TLR8	NM_016610.2	Endogenous	1.397262727	0.03107877
CD86	NM_175862.3	Endogenous	1.56960683	0.03340624
NLRP3	NM_001079821.2	Endogenous	1.281261025	0.03603107
C4A	NM_007293.2	Endogenous	1.588255542	0.03618749
CCL24	NM_002991.2	Endogenous	1.583986646	0.04663868
