# Supplementary material for: Gene expression profiling distinguishes prefibrotic from overtly fibrotic myeloproliferative neoplasms and identifies disease subsets with distinct inflammatory signatures
Source: PLoS One. 2019 May 9;14(5):e0216810. doi: 10.1371/journal.pone.0216810 (PMC6534080; doi:10.1371/journal.pone.0216810)
Supplement: S2 Table — (PDF) [file pone.0216810.s002.pdf]

**Supplementary Table 2. Gene ontology terms enriched in MPN with Grade 2-3 fibrosis**

| | |
|---|---|
| Analysis Type: | PANTHER Overrepresentation Test (Released 20171205) |
| Annotation Version and Release Date: | GO Ontology database Released 2018-08-09 |
| Analyzed List: | MPN Grade 2-3 (Homo sapiens) |
| Reference List: | Homo sapiens (all genes in database) |
| Test Type: | FISHER |

| GO biological process complete | REFLIST | MPN Grade 2-3 | Fold enrichment | FDR |
|---|---|---|---|---|
| inflammatory response (GO:0006954) | 466 | 56 | 19.76 | 4.29E-52 |
| response to biotic stimulus (GO:0009607) | 950 | 66 | 11.42 | 3.51E-49 |
| response to other organism (GO:0051707) | 911 | 65 | 11.73 | 5.19E-49 |
| response to external biotic stimulus (GO:0043207) | 913 | 65 | 11.7 | 5.26E-49 |
| cellular response to cytokine stimulus (GO:0071345) | 931 | 62 | 10.95 | 7.17E-45 |
| cytokine-mediated signaling pathway (GO:0019221) | 619 | 53 | 14.08 | 1.53E-42 |
| response to lipopolysaccharide (GO:0032496) | 307 | 39 | 20.88 | 4.08E-36 |
| response to bacterium (GO:0009617) | 643 | 48 | 12.27 | 1.83E-35 |
| response to molecule of bacterial origin (GO:0002237) | 323 | 39 | 19.85 | 2.30E-35 |
| regulation of cytokine production (GO:0001817) | 634 | 47 | 12.19 | 1.51E-34 |
| response to lipid (GO:0033993) | 846 | 51 | 9.91 | 7.70E-34 |
| positive regulation of immune response (GO:0050778) | 763 | 49 | 10.56 | 1.62E-33 |
| positive regulation of cytokine production (GO:0001819) | 403 | 40 | 16.32 | 2.17E-33 |
| regulation of response to external stimulus (GO:0032101) | 816 | 49 | 9.87 | 2.98E-32 |
| innate immune response (GO:0045087) | 708 | 46 | 10.68 | 2.14E-31 |
| cellular response to lipopolysaccharide (GO:0071222) | 179 | 30 | 27.55 | 1.76E-30 |
| cellular response to biotic stimulus (GO:0071216) | 206 | 31 | 24.74 | 2.65E-30 |
| cellular response to oxygen-containing compound (GO:1901701) | 960 | 50 | 8.56 | 2.69E-30 |
| cellular response to molecule of bacterial origin (GO:0071219) | 186 | 30 | 26.51 | 4.64E-30 |
| regulation of defense response (GO:0031347) | 755 | 44 | 9.58 | 4.41E-28 |

| | | | | |
|---|---|---|---|---|
| positive regulation of MAPK cascade (GO:0043410) | 552 | 39 | 11.61 | 1.99E-27 |
| cellular response to lipid (GO:0071396) | 522 | 38 | 11.97 | 4.33E-27 |
| positive regulation of protein phosphorylation (GO:0001934) | 990 | 45 | 7.47 | 1.34E-24 |
| activation of immune response (GO:0002253) | 550 | 36 | 10.76 | 4.29E-24 |
| regulation of cell migration (GO:0030334) | 799 | 40 | 8.23 | 5.54E-23 |
| regulation of MAPK cascade (GO:0043408) | 753 | 39 | 8.51 | 7.63E-23 |
| positive regulation of response to external stimulus (GO:0032103) | 284 | 28 | 16.21 | 9.53E-23 |
| regulation of cell motility (GO:2000145) | 854 | 40 | 7.7 | 5.41E-22 |
| response to virus (GO:0009615) | 275 | 27 | 16.14 | 7.57E-22 |
| protein phosphorylation (GO:0006468) | 927 | 41 | 7.27 | 9.86E-22 |
| regulation of locomotion (GO:0040012) | 930 | 41 | 7.25 | 1.09E-21 |
| defense response to other organism (GO:0098542) | 492 | 32 | 10.69 | 3.79E-21 |
| positive regulation of cell proliferation (GO:0008284) | 922 | 40 | 7.13 | 7.43E-21 |
| positive regulation of cell migration (GO:0030335) | 468 | 31 | 10.89 | 1.18E-20 |
| regulation of cellular component movement (GO:0051270) | 935 | 40 | 7.03 | 1.19E-20 |
| positive regulation of locomotion (GO:0040017) | 519 | 32 | 10.14 | 1.66E-20 |
| regulation of angiogenesis (GO:0045765) | 282 | 26 | 15.16 | 2.19E-20 |
| regulation of immune effector process (GO:0002697) | 436 | 30 | 11.31 | 2.23E-20 |
| positive regulation of cell motility (GO:2000147) | 486 | 31 | 10.49 | 3.21E-20 |
| positive regulation of cellular component movement (GO:0051272) | 500 | 31 | 10.19 | 7.00E-20 |
| regulation of leukocyte migration (GO:0002685) | 172 | 22 | 21.03 | 7.55E-20 |
| positive regulation of defense response (GO:0031349) | 378 | 28 | 12.18 | 1.03E-19 |
| response to interleukin-1 (GO:0070555) | 149 | 21 | 23.17 | 1.10E-19 |
| cellular response to interleukin-1 (GO:0071347) | 126 | 20 | 26.09 | 1.30E-19 |
| positive regulation of ERK1 and ERK2 cascade (GO:0070374) | 239 | 24 | 16.51 | 1.58E-19 |
| regulation of vasculature development (GO:1901342) | 310 | 26 | 13.79 | 1.86E-19 |
| immune response-regulating signaling pathway (GO:0002764) | 520 | 31 | 9.8 | 1.96E-19 |
| regulation of hemopoiesis (GO:1903706) | 391 | 28 | 11.77 | 2.30E-19 |
| regulation of inflammatory response (GO:0050727) | 410 | 28 | 11.23 | 7.34E-19 |
| positive regulation of cell differentiation (GO:0045597) | 939 | 38 | 6.65 | 9.64E-19 |
| cell chemotaxis (GO:0060326) | 198 | 22 | 18.27 | 1.10E-18 |

| | | | | |
|---|---|---|---|---|
| apoptotic process (GO:0006915) | 889 | 37 | 6.84 | 1.41E-18 |
| chemotaxis (GO:0006935) | 518 | 30 | 9.52 | 1.93E-18 |
| taxis (GO:0042330) | 520 | 30 | 9.48 | 2.12E-18 |
| regulation of ERK1 and ERK2 cascade (GO:0070372) | 311 | 25 | 13.21 | 2.79E-18 |
| signal transduction by protein phosphorylation (GO:0023014) | 356 | 26 | 12.01 | 4.14E-18 |
| leukocyte chemotaxis (GO:0030595) | 139 | 19 | 22.47 | 1.53E-17 |
| positive regulation of hemopoiesis (GO:1903708) | 181 | 20 | 18.16 | 7.21E-17 |
| response to chemokine (GO:1990868) | 82 | 16 | 32.08 | 8.02E-17 |
| cellular response to chemokine (GO:1990869) | 82 | 16 | 32.08 | 8.07E-17 |
| MAPK cascade (GO:0000165) | 328 | 24 | 12.03 | 1.19E-16 |
| positive regulation of kinase activity (GO:0033674) | 559 | 29 | 8.53 | 1.37E-16 |
| positive regulation of protein kinase activity (GO:0045860) | 518 | 28 | 8.89 | 2.08E-16 |
| positive regulation of leukocyte migration (GO:0002687) | 116 | 17 | 24.09 | 4.35E-16 |
| immune response-activating signal transduction (GO:0002757) | 485 | 27 | 9.15 | 4.40E-16 |
| regulation of transferase activity (GO:0051338) | 942 | 35 | 6.11 | 4.98E-16 |
| regulation of protein kinase activity (GO:0045859) | 765 | 32 | 6.88 | 6.63E-16 |
| chemokine-mediated signaling pathway (GO:0070098) | 76 | 15 | 32.45 | 8.06E-16 |
| regulation of kinase activity (GO:0043549) | 832 | 33 | 6.52 | 8.27E-16 |
| myeloid leukocyte migration (GO:0097529) | 123 | 17 | 22.72 | 1.02E-15 |
| response to mechanical stimulus (GO:0009612) | 213 | 20 | 15.44 | 1.17E-15 |
| response to interferon-gamma (GO:0034341) | 181 | 19 | 17.26 | 1.19E-15 |
| cellular response to mechanical stimulus (GO:0071260) | 80 | 15 | 30.82 | 1.54E-15 |
| neutrophil chemotaxis (GO:0030593) | 81 | 15 | 30.44 | 1.81E-15 |
| defense response to virus (GO:0051607) | 186 | 19 | 16.79 | 1.86E-15 |
| negative regulation of apoptotic process (GO:0043066) | 922 | 34 | 6.06 | 1.93E-15 |
| cellular response to interferon-gamma (GO:0071346) | 159 | 18 | 18.61 | 2.56E-15 |
| positive regulation of transferase activity (GO:0051347) | 631 | 29 | 7.56 | 2.64E-15 |
| negative regulation of programmed cell death (GO:0043069) | 936 | 34 | 5.97 | 2.94E-15 |
| positive regulation of MAP kinase activity (GO:0043406) | 264 | 21 | 13.08 | 3.70E-15 |
| regulation of leukocyte differentiation (GO:1902105) | 265 | 21 | 13.03 | 3.95E-15 |
| granulocyte chemotaxis (GO:0071621) | 88 | 15 | 28.02 | 5.21E-15 |

| | | | | |
|---|---|---|---|---|
| cellular response to tumor necrosis factor (GO:0071356) | 199 | 19 | 15.7 | 5.65E-15 |
| neutrophil migration (GO:1990266) | 90 | 15 | 27.4 | 6.96E-15 |
| response to organic cyclic compound (GO:0014070) | 901 | 33 | 6.02 | 7.27E-15 |
| positive regulation of cell death (GO:0010942) | 667 | 29 | 7.15 | 1.02E-14 |
| positive regulation of NF-kappaB transcription factor activity (GO:0051092) | 145 | 17 | 19.27 | 1.13E-14 |
| response to wounding (GO:0009611) | 560 | 27 | 7.93 | 1.17E-14 |
| granulocyte migration (GO:0097530) | 99 | 15 | 24.91 | 2.40E-14 |
| response to tumor necrosis factor (GO:0034612) | 222 | 19 | 14.07 | 3.48E-14 |
| positive regulation of protein serine/threonine kinase activity (GO:0071902) | 341 | 22 | 10.61 | 3.55E-14 |
| humoral immune response (GO:0006959) | 345 | 22 | 10.48 | 4.38E-14 |
| innate immune response-activating signal transduction (GO:0002758) | 166 | 17 | 16.84 | 8.49E-14 |
| lipopolysaccharide-mediated signaling pathway (GO:0031663) | 32 | 11 | 56.51 | 9.94E-14 |
| leukocyte migration (GO:0050900) | 361 | 22 | 10.02 | 1.05E-13 |
| regulation of DNA-binding transcription factor activity (GO:0051090) | 408 | 23 | 9.27 | 1.08E-13 |
| regulation of signaling receptor activity (GO:0010469) | 562 | 26 | 7.61 | 1.10E-13 |
| positive regulation of leukocyte differentiation (GO:1902107) | 143 | 16 | 18.39 | 1.66E-13 |
| regulation of multi-organism process (GO:0043900) | 374 | 22 | 9.67 | 2.02E-13 |
| response to hormone (GO:0009725) | 889 | 31 | 5.73 | 2.42E-13 |
| regulation of interleukin-12 production (GO:0032655) | 52 | 12 | 37.94 | 2.68E-13 |
| regulation of protein secretion (GO:0050708) | 430 | 23 | 8.79 | 2.98E-13 |
| positive regulation of DNA-binding transcription factor activity (GO:0051091) | 254 | 19 | 12.3 | 3.12E-13 |
| activation of innate immune response (GO:0002218) | 184 | 17 | 15.19 | 3.78E-13 |
| regulation of MAP kinase activity (GO:0043405) | 341 | 21 | 10.12 | 3.79E-13 |
| regulation of I-kappaB kinase/NF-kappaB signaling (GO:0043122) | 222 | 18 | 13.33 | 4.63E-13 |
| cell migration (GO:0016477) | 913 | 31 | 5.58 | 4.68E-13 |
| cellular response to environmental stimulus (GO:0104004) | 304 | 20 | 10.82 | 5.37E-13 |
| cellular response to abiotic stimulus (GO:0071214) | 304 | 20 | 10.82 | 5.39E-13 |
| regulation of adaptive immune response (GO:0002819) | 156 | 16 | 16.86 | 5.42E-13 |
| positive regulation of apoptotic process (GO:0043065) | 609 | 26 | 7.02 | 6.15E-13 |
| positive regulation of innate immune response (GO:0045089) | 265 | 19 | 11.79 | 6.16E-13 |
| positive regulation of programmed cell death (GO:0043068) | 615 | 26 | 6.95 | 7.60E-13 |

| | | | | |
|---|---|---|---|---|
| adaptive immune response based on somatic recombination of immune receptors built from immunoglobulin superfamily domains (GO:0002460) | 269 | 19 | 11.61 | 7.77E-13 |
| regulation of protein serine/threonine kinase activity (GO:0071900) | 506 | 24 | 7.8 | 8.22E-13 |
| positive regulation of cytokine biosynthetic process (GO:0042108) | 59 | 12 | 33.44 | 9.26E-13 |
| regulation of peptide secretion (GO:0002791) | 458 | 23 | 8.26 | 9.79E-13 |
| positive regulation of angiogenesis (GO:0045766) | 163 | 16 | 16.14 | 9.80E-13 |
| cellular response to external stimulus (GO:0071496) | 318 | 20 | 10.34 | 1.14E-12 |
| regulation of innate immune response (GO:0045088) | 365 | 21 | 9.46 | 1.24E-12 |
| positive regulation of hydrolase activity (GO:0051345) | 754 | 28 | 6.1 | 1.33E-12 |
| wound healing (GO:0042060) | 470 | 23 | 8.04 | 1.61E-12 |
| pattern recognition receptor signaling pathway (GO:0002221) | 110 | 14 | 20.92 | 1.75E-12 |
| regulation of type I interferon production (GO:0032479) | 110 | 14 | 20.92 | 1.75E-12 |
| positive regulation of type I interferon production (GO:0032481) | 64 | 12 | 30.82 | 2.09E-12 |
| cellular cation homeostasis (GO:0030003) | 589 | 25 | 6.98 | 2.28E-12 |
| cellular response to type I interferon (GO:0071357) | 66 | 12 | 29.89 | 2.87E-12 |
| type I interferon signaling pathway (GO:0060337) | 66 | 12 | 29.89 | 2.88E-12 |
| cellular ion homeostasis (GO:0006873) | 603 | 25 | 6.82 | 3.72E-12 |
| positive regulation of interleukin-12 production (GO:0032735) | 32 | 10 | 51.37 | 3.73E-12 |
| positive regulation of vasculature development (GO:1904018) | 180 | 16 | 14.61 | 3.84E-12 |
| regulation of leukocyte proliferation (GO:0070663) | 218 | 17 | 12.82 | 4.39E-12 |
| negative regulation of cell proliferation (GO:0008285) | 676 | 26 | 6.32 | 5.72E-12 |
| activation of MAPK activity (GO:0000187) | 152 | 15 | 16.22 | 5.88E-12 |
| response to type I interferon (GO:0034340) | 71 | 12 | 27.78 | 6.03E-12 |
| regulation of myeloid cell differentiation (GO:0045637) | 223 | 17 | 12.53 | 6.08E-12 |
| positive regulation of transport (GO:0051050) | 943 | 30 | 5.23 | 6.22E-12 |
| response to drug (GO:0042493) | 956 | 30 | 5.16 | 8.64E-12 |
| regulation of cytokine biosynthetic process (GO:0042035) | 98 | 13 | 21.81 | 8.70E-12 |
| positive regulation of chemotaxis (GO:0050921) | 126 | 14 | 18.27 | 8.88E-12 |
| cellular calcium ion homeostasis (GO:0006874) | 409 | 21 | 8.44 | 9.15E-12 |
| regulation of leukocyte chemotaxis (GO:0002688) | 102 | 13 | 20.95 | 1.37E-11 |
| regulation of chemotaxis (GO:0050920) | 198 | 16 | 13.28 | 1.42E-11 |

| | | | | |
|---|---|---|---|---|
| calcium ion homeostasis (GO:0055074) | 422 | 21 | 8.18 | 1.61E-11 |
| cellular metal ion homeostasis (GO:0006875) | 530 | 23 | 7.13 | 1.61E-11 |
| positive regulation of protein secretion (GO:0050714) | 244 | 17 | 11.45 | 2.27E-11 |
| cation homeostasis (GO:0055080) | 660 | 25 | 6.23 | 2.38E-11 |
| regulation of cell-cell adhesion (GO:0022407) | 382 | 20 | 8.61 | 2.54E-11 |
| stress-activated MAPK cascade (GO:0051403) | 108 | 13 | 19.79 | 2.60E-11 |
| adaptive immune response (GO:0002250) | 544 | 23 | 6.95 | 2.65E-11 |
| regulation of neuron death (GO:1901214) | 292 | 18 | 10.13 | 3.06E-11 |
| cellular divalent inorganic cation homeostasis (GO:0072503) | 441 | 21 | 7.83 | 3.51E-11 |
| inorganic ion homeostasis (GO:0098771) | 673 | 25 | 6.11 | 3.53E-11 |
| ion homeostasis (GO:0050801) | 737 | 26 | 5.8 | 3.53E-11 |
| positive regulation of I-kappaB kinase/NF-kappaB signaling (GO:0043123) | 175 | 15 | 14.09 | 3.58E-11 |
| stress-activated protein kinase signaling cascade (GO:0031098) | 145 | 14 | 15.87 | 4.90E-11 |
| positive regulation of myeloid cell differentiation (GO:0045639) | 88 | 12 | 22.42 | 5.45E-11 |
| cellular chemical homeostasis (GO:0055082) | 691 | 25 | 5.95 | 6.07E-11 |
| positive regulation of peptide secretion (GO:0002793) | 264 | 17 | 10.59 | 7.12E-11 |
| regulation of secretion (GO:0051046) | 763 | 26 | 5.6 | 7.36E-11 |
| divalent inorganic cation homeostasis (GO:0072507) | 462 | 21 | 7.47 | 7.89E-11 |
| activation of protein kinase activity (GO:0032147) | 313 | 18 | 9.45 | 8.82E-11 |
| response to growth factor (GO:0070848) | 521 | 22 | 6.94 | 8.82E-11 |
| I-kappaB kinase/NF-kappaB signaling (GO:0007249) | 68 | 11 | 26.59 | 9.08E-11 |
| cellular homeostasis (GO:0019725) | 841 | 27 | 5.28 | 9.56E-11 |
| regulation of establishment of protein localization (GO:0070201) | 708 | 25 | 5.8 | 9.80E-11 |
| regulation of acute inflammatory response (GO:0002673) | 154 | 14 | 14.94 | 9.98E-11 |
| interspecies interaction between organisms (GO:0044419) | 709 | 25 | 5.8 | 9.99E-11 |
| negative regulation of developmental process (GO:0051093) | 918 | 28 | 5.01 | 1.12E-10 |
| positive regulation of tumor necrosis factor superfamily cytokine production (GO:1903557) | 70 | 11 | 25.83 | 1.18E-10 |
| regulation of epithelial cell proliferation (GO:0050678) | 320 | 18 | 9.25 | 1.21E-10 |
| regulation of cell adhesion (GO:0030155) | 655 | 24 | 6.02 | 1.33E-10 |
| response to ketone (GO:1901654) | 195 | 15 | 12.65 | 1.43E-10 |

| | | | | |
|---|---|---|---|---|
| metal ion homeostasis (GO:0055065) | 597 | 23 | 6.33 | 1.51E-10 |
| cellular response to nitrogen compound (GO:1901699) | 599 | 23 | 6.31 | 1.61E-10 |
| interleukin-1-mediated signaling pathway (GO:0070498) | 51 | 10 | 32.23 | 1.69E-10 |
| symbiont process (GO:0044403) | 664 | 24 | 5.94 | 1.73E-10 |
| regulation of protein transport (GO:0051223) | 664 | 24 | 5.94 | 1.73E-10 |
| regulation of cell activation (GO:0050865) | 607 | 23 | 6.23 | 2.06E-10 |
| regulation of leukocyte cell-cell adhesion (GO:1903037) | 286 | 17 | 9.77 | 2.19E-10 |
| immune system development (GO:0002520) | 618 | 23 | 6.12 | 2.89E-10 |
| positive regulation of cell-cell adhesion (GO:0022409) | 248 | 16 | 10.61 | 3.04E-10 |
| positive regulation of leukocyte chemotaxis (GO:0002690) | 79 | 11 | 22.89 | 3.68E-10 |
| regulation of mononuclear cell proliferation (GO:0032944) | 211 | 15 | 11.69 | 3.94E-10 |
| positive regulation of cell adhesion (GO:0045785) | 397 | 19 | 7.87 | 3.97E-10 |
| regulation of peptide transport (GO:0090087) | 694 | 24 | 5.68 | 4.11E-10 |
| regulation of secretion by cell (GO:1903530) | 705 | 24 | 5.6 | 5.58E-10 |
| regulation of mononuclear cell migration (GO:0071675) | 39 | 9 | 37.94 | 5.67E-10 |
| regulation of adaptive immune response based on somatic recombination of immune receptors built from immunoglobulin superfamily domains (GO:0002822) | 144 | 13 | 14.84 | 6.39E-10 |
| regulation of cytokine secretion (GO:0050707) | 180 | 14 | 12.79 | 6.47E-10 |
| positive regulation of neuron death (GO:1901216) | 84 | 11 | 21.53 | 6.52E-10 |
| regulation of ossification (GO:0030278) | 181 | 14 | 12.72 | 6.89E-10 |
| toll-like receptor signaling pathway (GO:0002224) | 85 | 11 | 21.27 | 7.28E-10 |
| regulation of proteolysis (GO:0030162) | 789 | 25 | 5.21 | 8.53E-10 |
| positive regulation of cytosolic calcium ion concentration (GO:0007204) | 268 | 16 | 9.81 | 8.70E-10 |
| negative regulation of immune system process (GO:0002683) | 423 | 19 | 7.38 | 1.09E-09 |
| cellular response to hormone stimulus (GO:0032870) | 601 | 22 | 6.02 | 1.13E-09 |
| regulation of interleukin-6 production (GO:0032675) | 119 | 12 | 16.58 | 1.19E-09 |
| immune response-regulating cell surface receptor signaling pathway (GO:0002768) | 426 | 19 | 7.33 | 1.20E-09 |
| positive regulation of cytokine secretion (GO:0050715) | 121 | 12 | 16.3 | 1.42E-09 |
| cellular response to drug (GO:0035690) | 327 | 17 | 8.55 | 1.51E-09 |
| regulation of tumor necrosis factor superfamily cytokine production (GO:1903555) | 122 | 12 | 16.17 | 1.54E-09 |
| positive regulation of tumor necrosis factor production (GO:0032760) | 67 | 10 | 24.54 | 1.71E-09 |

| | | | | |
|---|---|---|---|---|
| positive regulation of establishment of protein localization (GO:1904951) | 436 | 19 | 7.16 | 1.73E-09 |
| regulation of endothelial cell proliferation (GO:0001936) | 124 | 12 | 15.91 | 1.81E-09 |
| leukocyte activation (GO:0045321) | 892 | 26 | 4.79 | 1.81E-09 |
| lymphocyte chemotaxis (GO:0048247) | 46 | 9 | 32.16 | 1.92E-09 |
| regulation of cytokine production involved in immune response (GO:0002718) | 68 | 10 | 24.18 | 1.92E-09 |
| regulation of lymphocyte activation (GO:0051249) | 497 | 20 | 6.62 | 1.93E-09 |
| regulation of production of molecular mediator of immune response (GO:0002700) | 125 | 12 | 15.78 | 1.94E-09 |
| enzyme linked receptor protein signaling pathway (GO:0007167) | 686 | 23 | 5.51 | 1.96E-09 |
| response to organonitrogen compound (GO:0010243) | 899 | 26 | 4.75 | 2.08E-09 |
| positive regulation of stress-activated MAPK cascade (GO:0032874) | 161 | 13 | 13.27 | 2.15E-09 |
| positive regulation of stress-activated protein kinase signaling cascade (GO:0070304) | 162 | 13 | 13.19 | 2.30E-09 |
| positive regulation of interleukin-8 production (GO:0032757) | 48 | 9 | 30.82 | 2.63E-09 |
| regulation of leukocyte activation (GO:0002694) | 572 | 21 | 6.04 | 3.00E-09 |
| regulation of interferon-beta production (GO:0032648) | 49 | 9 | 30.19 | 3.07E-09 |
| regulation of smooth muscle cell proliferation (GO:0048660) | 131 | 12 | 15.06 | 3.13E-09 |
| regulation of humoral immune response (GO:0002920) | 132 | 12 | 14.94 | 3.38E-09 |
| positive regulation of protein transport (GO:0051222) | 401 | 18 | 7.38 | 3.53E-09 |
| cellular response to organic cyclic compound (GO:0071407) | 518 | 20 | 6.35 | 3.76E-09 |
| regulation of lymphocyte proliferation (GO:0050670) | 210 | 14 | 10.96 | 3.95E-09 |
| positive regulation of leukocyte cell-cell adhesion (GO:1903039) | 210 | 14 | 10.96 | 3.96E-09 |
| positive regulation of interferon-beta production (GO:0032728) | 32 | 8 | 41.1 | 4.19E-09 |
| regulation of T cell activation (GO:0050863) | 305 | 16 | 8.62 | 4.84E-09 |
| positive regulation of mononuclear cell proliferation (GO:0032946) | 137 | 12 | 14.4 | 4.93E-09 |
| positive regulation of JNK cascade (GO:0046330) | 138 | 12 | 14.29 | 5.32E-09 |
| regulation of cytosolic calcium ion concentration (GO:0051480) | 308 | 16 | 8.54 | 5.49E-09 |
| positive regulation of chemokine production (GO:0032722) | 53 | 9 | 27.92 | 5.53E-09 |
| positive regulation of leukocyte proliferation (GO:0070665) | 140 | 12 | 14.09 | 6.16E-09 |
| regulation of stress-activated MAPK cascade (GO:0032872) | 221 | 14 | 10.41 | 7.26E-09 |
| regulation of stress-activated protein kinase signaling cascade (GO:0070302) | 222 | 14 | 10.37 | 7.66E-09 |
| regulation of protein localization (GO:0032880) | 960 | 26 | 4.45 | 7.80E-09 |
| positive regulation of secretion by cell (GO:1903532) | 369 | 17 | 7.57 | 8.09E-09 |

| | | | | |
|---|---|---|---|---|
| positive regulation of interleukin-6 production (GO:0032755) | 81 | 10 | 20.3 | 8.40E-09 |
| cytokine production (GO:0001816) | 145 | 12 | 13.6 | 8.84E-09 |
| regulation of body fluid levels (GO:0050878) | 489 | 19 | 6.39 | 1.01E-08 |
| cellular response to growth factor stimulus (GO:0071363) | 490 | 19 | 6.37 | 1.04E-08 |
| regulation of myeloid leukocyte differentiation (GO:0002761) | 115 | 11 | 15.72 | 1.23E-08 |
| response to peptide (GO:1901652) | 437 | 18 | 6.77 | 1.26E-08 |
| JNK cascade (GO:0007254) | 86 | 10 | 19.12 | 1.41E-08 |
| lymphocyte migration (GO:0072676) | 60 | 9 | 24.66 | 1.43E-08 |
| regulation of tumor necrosis factor production (GO:0032680) | 118 | 11 | 15.32 | 1.57E-08 |
| regulation of granulocyte chemotaxis (GO:0071622) | 40 | 8 | 32.88 | 1.85E-08 |
| response to extracellular stimulus (GO:0009991) | 509 | 19 | 6.14 | 1.87E-08 |
| regulation of neuron apoptotic process (GO:0043523) | 199 | 13 | 10.74 | 2.25E-08 |
| positive regulation of leukocyte activation (GO:0002696) | 398 | 17 | 7.02 | 2.34E-08 |
| immunoglobulin mediated immune response (GO:0016064) | 200 | 13 | 10.69 | 2.37E-08 |
| positive regulation of T cell activation (GO:0050870) | 201 | 13 | 10.63 | 2.49E-08 |
| B cell mediated immunity (GO:0019724) | 201 | 13 | 10.63 | 2.49E-08 |
| positive regulation of secretion (GO:0051047) | 400 | 17 | 6.99 | 2.49E-08 |
| monocyte chemotaxis (GO:0002548) | 42 | 8 | 31.31 | 2.53E-08 |
| hematopoietic or lymphoid organ development (GO:0048534) | 585 | 20 | 5.62 | 2.64E-08 |
| positive regulation of inflammatory response (GO:0050729) | 126 | 11 | 14.35 | 2.87E-08 |
| positive regulation of interferon-gamma production (GO:0032729) | 66 | 9 | 22.42 | 2.94E-08 |
| viral process (GO:0016032) | 589 | 20 | 5.58 | 2.94E-08 |
| regulation of symbiosis, encompassing mutualism through parasitism (GO:0043903) | 205 | 13 | 10.42 | 3.07E-08 |
| positive regulation of cell activation (GO:0050867) | 408 | 17 | 6.85 | 3.26E-08 |
| regulation of interleukin-8 production (GO:0032677) | 67 | 9 | 22.08 | 3.28E-08 |
| regulation of response to cytokine stimulus (GO:0060759) | 165 | 12 | 11.96 | 3.28E-08 |
| mononuclear cell migration (GO:0071674) | 44 | 8 | 29.89 | 3.43E-08 |
| negative regulation of cytokine production (GO:0001818) | 253 | 14 | 9.1 | 3.51E-08 |
| positive regulation of NIK/NF-kappaB signaling (GO:1901224) | 68 | 9 | 21.76 | 3.67E-08 |
| regulation of epithelial cell migration (GO:0010632) | 210 | 13 | 10.18 | 3.97E-08 |
| positive regulation of lymphocyte activation (GO:0051251) | 359 | 16 | 7.33 | 4.13E-08 |

| | | | | |
|---|---|---|---|---|
| positive regulation of adaptive immune response based on somatic recombination of immune receptors built from immunoglobulin superfamily domains (GO:0002824) | 99 | 10 | 16.61 | 4.57E-08 |
| regulation of NIK/NF-kappaB signaling (GO:1901222) | 99 | 10 | 16.61 | 4.58E-08 |
| regulation of interferon-gamma production (GO:0032649) | 99 | 10 | 16.61 | 4.59E-08 |
| response to nutrient levels (GO:0031667) | 478 | 18 | 6.19 | 4.59E-08 |
| transmembrane receptor protein tyrosine kinase signaling pathway (GO:0007169) | 485 | 18 | 6.1 | 5.66E-08 |
| positive regulation of lymphocyte proliferation (GO:0050671) | 136 | 11 | 13.3 | 5.81E-08 |
| regulation of chemokine production (GO:0032642) | 73 | 9 | 20.27 | 6.32E-08 |
| positive regulation of adaptive immune response (GO:0002821) | 103 | 10 | 15.96 | 6.44E-08 |
| leukocyte differentiation (GO:0002521) | 319 | 15 | 7.73 | 6.92E-08 |
| negative regulation of angiogenesis (GO:0016525) | 104 | 10 | 15.81 | 6.99E-08 |
| positive regulation of neuron apoptotic process (GO:0043525) | 50 | 8 | 26.3 | 8.12E-08 |
| negative regulation of blood vessel morphogenesis (GO:2000181) | 106 | 10 | 15.51 | 8.25E-08 |
| regulation of JNK cascade (GO:0046328) | 181 | 12 | 10.9 | 8.37E-08 |
| apoptotic signaling pathway (GO:0097190) | 277 | 14 | 8.31 | 1.02E-07 |
| cellular response to organonitrogen compound (GO:0071417) | 506 | 18 | 5.85 | 1.05E-07 |
| positive regulation of myeloid leukocyte differentiation (GO:0002763) | 52 | 8 | 25.29 | 1.06E-07 |
| regulation of cellular response to stress (GO:0080135) | 647 | 20 | 5.08 | 1.30E-07 |
| regulation of myoblast differentiation (GO:0045661) | 54 | 8 | 24.35 | 1.37E-07 |
| regulation of complement activation (GO:0030449) | 113 | 10 | 14.55 | 1.43E-07 |
| regulation of neutrophil migration (GO:1902622) | 33 | 7 | 34.87 | 1.46E-07 |
| regulation of endothelial cell migration (GO:0010594) | 150 | 11 | 12.06 | 1.46E-07 |
| regulation of protein activation cascade (GO:2000257) | 114 | 10 | 14.42 | 1.53E-07 |
| positive regulation of interleukin-1 beta production (GO:0032731) | 34 | 7 | 33.85 | 1.72E-07 |
| positive regulation of proteolysis (GO:0045862) | 345 | 15 | 7.15 | 1.81E-07 |
| negative regulation of vasculature development (GO:1901343) | 117 | 10 | 14.05 | 1.90E-07 |
| regulation of interleukin-1 beta production (GO:0032651) | 58 | 8 | 22.67 | 2.21E-07 |
| defense response to Gram-positive bacterium (GO:0050830) | 86 | 9 | 17.2 | 2.22E-07 |
| response to transforming growth factor beta (GO:0071559) | 157 | 11 | 11.52 | 2.22E-07 |
| regulation of T cell proliferation (GO:0042129) | 157 | 11 | 11.52 | 2.22E-07 |
| regulation of blood vessel endothelial cell migration (GO:0043535) | 87 | 9 | 17.01 | 2.42E-07 |

| | | | | |
|---|---|---|---|---|
| defense response to bacterium (GO:0042742) | 301 | 14 | 7.65 | 2.63E-07 |
| positive regulation of multi-organism process (GO:0043902) | 162 | 11 | 11.16 | 2.98E-07 |
| lymphocyte mediated immunity (GO:0002449) | 258 | 13 | 8.28 | 3.75E-07 |
| response to toxic substance (GO:0009636) | 488 | 17 | 5.73 | 3.79E-07 |
| adenylate cyclase-activating G-protein coupled receptor signaling pathway (GO:0007189) | 127 | 10 | 12.94 | 3.85E-07 |
| regulation of toll-like receptor signaling pathway (GO:0034121) | 63 | 8 | 20.88 | 3.88E-07 |
| eosinophil chemotaxis (GO:0048245) | 21 | 6 | 46.97 | 4.05E-07 |
| eosinophil migration (GO:0072677) | 22 | 6 | 44.83 | 5.10E-07 |
| positive regulation of interferon-alpha production (GO:0032727) | 22 | 6 | 44.83 | 5.11E-07 |
| antimicrobial humoral immune response mediated by antimicrobial peptide (GO:0061844) | 66 | 8 | 19.93 | 5.36E-07 |
| vascular endothelial growth factor receptor signaling pathway (GO:0048010) | 67 | 8 | 19.63 | 5.94E-07 |
| regulation of response to biotic stimulus (GO:0002831) | 134 | 10 | 12.27 | 6.09E-07 |
| regulation of T cell differentiation (GO:0045580) | 134 | 10 | 12.27 | 6.10E-07 |
| negative regulation of multi-organism process (GO:0043901) | 176 | 11 | 10.27 | 6.46E-07 |
| regulation of viral process (GO:0050792) | 177 | 11 | 10.22 | 6.81E-07 |
| positive regulation of pri-miRNA transcription by RNA polymerase II (GO:1902895) | 24 | 6 | 41.1 | 7.75E-07 |
| detection of biotic stimulus (GO:0009595) | 24 | 6 | 41.1 | 7.76E-07 |
| positive regulation of cellular component biogenesis (GO:0044089) | 515 | 17 | 5.43 | 7.80E-07 |
| positive regulation of interleukin-1 production (GO:0032732) | 44 | 7 | 26.15 | 7.83E-07 |
| immune response-activating cell surface receptor signaling pathway (GO:0002429) | 392 | 15 | 6.29 | 8.63E-07 |
| positive regulation of peptidase activity (GO:0010952) | 183 | 11 | 9.88 | 9.26E-07 |
| positive regulation of epithelial cell proliferation (GO:0050679) | 185 | 11 | 9.77 | 1.03E-06 |
| regulation of interleukin-1 production (GO:0032652) | 74 | 8 | 17.77 | 1.17E-06 |
| response to inorganic substance (GO:0010035) | 532 | 17 | 5.25 | 1.22E-06 |
| cAMP-mediated signaling (GO:0019933) | 146 | 10 | 11.26 | 1.27E-06 |
| blood coagulation (GO:0007596) | 289 | 13 | 7.39 | 1.27E-06 |
| regulation of phagocytosis (GO:0050764) | 75 | 8 | 17.54 | 1.28E-06 |
| coagulation (GO:0050817) | 291 | 13 | 7.34 | 1.37E-06 |
| positive regulation of neutrophil migration (GO:1902624) | 27 | 6 | 36.53 | 1.39E-06 |

| | | | | |
|---|---|---|---|---|
| hemopoiesis (GO:0030097) | 538 | 17 | 5.19 | 1.41E-06 |
| cellular response to transforming growth factor beta stimulus (GO:0071560) | 149 | 10 | 11.03 | 1.50E-06 |
| hemostasis (GO:0007599) | 294 | 13 | 7.27 | 1.52E-06 |
| circulatory system development (GO:0072359) | 839 | 21 | 4.11 | 1.57E-06 |
| G-protein coupled receptor signaling pathway, coupled to cyclic nucleotide second messenger (GO:0007187) | 243 | 12 | 8.12 | 1.62E-06 |
| regulation of interferon-alpha production (GO:0032647) | 28 | 6 | 35.23 | 1.65E-06 |
| programmed necrotic cell death (GO:0097300) | 28 | 6 | 35.23 | 1.65E-06 |
| animal organ morphogenesis (GO:0009887) | 924 | 22 | 3.91 | 1.69E-06 |
| cellular response to peptide (GO:1901653) | 300 | 13 | 7.12 | 1.88E-06 |
| regulation of cytokine-mediated signaling pathway (GO:0001959) | 155 | 10 | 10.61 | 2.07E-06 |
| positive regulation of response to cytokine stimulus (GO:0060760) | 52 | 7 | 22.13 | 2.10E-06 |
| lymphocyte activation (GO:0046649) | 362 | 14 | 6.36 | 2.16E-06 |
| positive regulation of immune effector process (GO:0002699) | 201 | 11 | 9 | 2.17E-06 |
| necrotic cell death (GO:0070265) | 30 | 6 | 32.88 | 2.31E-06 |
| regulation of lymphocyte differentiation (GO:0045619) | 163 | 10 | 10.09 | 3.17E-06 |
| positive regulation of nitric-oxide synthase biosynthetic process (GO:0051770) | 15 | 5 | 54.8 | 3.37E-06 |
| negative regulation of epithelial cell proliferation (GO:0050680) | 124 | 9 | 11.93 | 3.68E-06 |
| positive regulation of endopeptidase activity (GO:0010950) | 166 | 10 | 9.9 | 3.70E-06 |
| MyD88-dependent toll-like receptor signaling pathway (GO:0002755) | 33 | 6 | 29.89 | 3.74E-06 |
| tube development (GO:0035295) | 813 | 20 | 4.04 | 4.25E-06 |
| regulation of reactive oxygen species metabolic process (GO:2000377) | 169 | 10 | 9.73 | 4.31E-06 |
| cyclic-nucleotide-mediated signaling (GO:0019935) | 169 | 10 | 9.73 | 4.31E-06 |
| regulation of pri-miRNA transcription by RNA polymerase II (GO:1902893) | 34 | 6 | 29.01 | 4.35E-06 |
| response to nutrient (GO:0007584) | 219 | 11 | 8.26 | 4.81E-06 |
| second-messenger-mediated signaling (GO:0019932) | 329 | 13 | 6.5 | 4.98E-06 |
| negative regulation of apoptotic signaling pathway (GO:2001234) | 222 | 11 | 8.15 | 5.46E-06 |
| regulation of actin cytoskeleton organization (GO:0032956) | 332 | 13 | 6.44 | 5.48E-06 |
| positive regulation of organelle organization (GO:0010638) | 597 | 17 | 4.68 | 5.52E-06 |
| regulation of protein processing (GO:0070613) | 175 | 10 | 9.39 | 5.77E-06 |
| negative regulation of viral process (GO:0048525) | 94 | 8 | 13.99 | 5.92E-06 |

| | | | | |
|---|---|---|---|---|
| positive regulation of lymphocyte differentiation (GO:0045621) | 94 | 8 | 13.99 | 5.93E-06 |
| negative regulation of transcription by RNA polymerase II (GO:0000122) | 833 | 20 | 3.95 | 6.05E-06 |
| regulation of protein maturation (GO:1903317) | 177 | 10 | 9.29 | 6.32E-06 |
| leukocyte mediated immunity (GO:0002443) | 759 | 19 | 4.12 | 6.78E-06 |
| cellular response to ketone (GO:1901655) | 96 | 8 | 13.7 | 6.82E-06 |
| transforming growth factor beta receptor signaling pathway (GO:0007179) | 96 | 8 | 13.7 | 6.83E-06 |
| regulation of cellular component biogenesis (GO:0044087) | 923 | 21 | 3.74 | 6.86E-06 |
| protein kinase B signaling (GO:0043491) | 38 | 6 | 25.96 | 7.58E-06 |
| negative regulation of endothelial cell proliferation (GO:0001937) | 38 | 6 | 25.96 | 7.60E-06 |
| negative regulation of cell differentiation (GO:0045596) | 688 | 18 | 4.3 | 7.69E-06 |
| platelet activation (GO:0030168) | 137 | 9 | 10.8 | 7.77E-06 |
| positive regulation of epithelial cell migration (GO:0010634) | 137 | 9 | 10.8 | 7.78E-06 |
| positive regulation of T cell proliferation (GO:0042102) | 98 | 8 | 13.42 | 7.80E-06 |
| transcription by RNA polymerase II (GO:0006366) | 770 | 19 | 4.06 | 8.23E-06 |
| regulation of nitric-oxide synthase biosynthetic process (GO:0051769) | 19 | 5 | 43.26 | 8.52E-06 |
| regulation of monocyte differentiation (GO:0045655) | 19 | 5 | 43.26 | 8.53E-06 |
| blood vessel development (GO:0001568) | 478 | 15 | 5.16 | 9.05E-06 |
| response to reactive oxygen species (GO:0000302) | 187 | 10 | 8.79 | 9.94E-06 |
| response to alcohol (GO:0097305) | 240 | 11 | 7.53 | 1.10E-05 |
| detection of external biotic stimulus (GO:0098581) | 21 | 5 | 39.14 | 1.29E-05 |
| interferon-gamma-mediated signaling pathway (GO:0060333) | 71 | 7 | 16.21 | 1.34E-05 |
| negative regulation of ossification (GO:0030279) | 71 | 7 | 16.21 | 1.34E-05 |
| myeloid leukocyte differentiation (GO:0002573) | 106 | 8 | 12.41 | 1.34E-05 |
| negative regulation of extrinsic apoptotic signaling pathway (GO:2001237) | 106 | 8 | 12.41 | 1.34E-05 |
| negative regulation of cell migration (GO:0030336) | 249 | 11 | 7.26 | 1.53E-05 |
| necroptotic process (GO:0070266) | 22 | 5 | 37.36 | 1.55E-05 |
| regulation of macrophage chemotaxis (GO:0010758) | 22 | 5 | 37.36 | 1.55E-05 |
| positive regulation of mononuclear cell migration (GO:0071677) | 22 | 5 | 37.36 | 1.56E-05 |
| vasculature development (GO:0001944) | 501 | 15 | 4.92 | 1.57E-05 |
| response to antibiotic (GO:0046677) | 309 | 12 | 6.38 | 1.69E-05 |
| heart development (GO:0007507) | 505 | 15 | 4.88 | 1.72E-05 |

| | | | | |
|---|---|---|---|---|
| antimicrobial humoral response (GO:0019730) | 111 | 8 | 11.85 | 1.81E-05 |
| positive regulation of neutrophil chemotaxis (GO:0090023) | 23 | 5 | 35.74 | 1.85E-05 |
| response to peptide hormone (GO:0043434) | 375 | 13 | 5.7 | 1.91E-05 |
| regulation of actin filament-based process (GO:0032970) | 375 | 13 | 5.7 | 1.92E-05 |
| regulation of endothelial cell apoptotic process (GO:2000351) | 46 | 6 | 21.44 | 1.97E-05 |
| positive regulation of JUN kinase activity (GO:0043507) | 76 | 7 | 15.14 | 1.99E-05 |
| cardiovascular system development (GO:0072358) | 512 | 15 | 4.82 | 2.00E-05 |
| peptidyl-serine phosphorylation (GO:0018105) | 156 | 9 | 9.48 | 2.05E-05 |
| regulation of cytoskeleton organization (GO:0051493) | 515 | 15 | 4.79 | 2.14E-05 |
| negative regulation of cytokine production involved in immune response (GO:0002719) | 24 | 5 | 34.25 | 2.19E-05 |
| adenylate cyclase-modulating G-protein coupled receptor signaling pathway (GO:0007188) | 207 | 10 | 7.94 | 2.28E-05 |
| negative regulation of cell motility (GO:2000146) | 264 | 11 | 6.85 | 2.54E-05 |
| positive regulation of granulocyte chemotaxis (GO:0071624) | 25 | 5 | 32.88 | 2.59E-05 |
| positive regulation of interleukin-1 beta secretion (GO:0050718) | 25 | 5 | 32.88 | 2.59E-05 |
| tumor necrosis factor-mediated signaling pathway (GO:0033209) | 80 | 7 | 14.38 | 2.68E-05 |
| regulation of animal organ morphogenesis (GO:2000027) | 212 | 10 | 7.75 | 2.77E-05 |
| regulation of apoptotic signaling pathway (GO:2001233) | 390 | 13 | 5.48 | 2.84E-05 |
| regulation of vascular smooth muscle cell proliferation (GO:1904705) | 50 | 6 | 19.73 | 2.99E-05 |
| positive regulation of T cell differentiation (GO:0045582) | 82 | 7 | 14.03 | 3.10E-05 |
| blood vessel morphogenesis (GO:0048514) | 396 | 13 | 5.4 | 3.32E-05 |
| humoral immune response mediated by circulating immunoglobulin (GO:0002455) | 167 | 9 | 8.86 | 3.40E-05 |
| positive regulation of vascular endothelial growth factor production (GO:0010575) | 27 | 5 | 30.44 | 3.54E-05 |
| regulation of macrophage migration (GO:1905521) | 27 | 5 | 30.44 | 3.55E-05 |
| positive regulation of smooth muscle cell proliferation (GO:0048661) | 84 | 7 | 13.7 | 3.56E-05 |
| leukocyte proliferation (GO:0070661) | 84 | 7 | 13.7 | 3.57E-05 |
| intracellular receptor signaling pathway (GO:0030522) | 170 | 9 | 8.7 | 3.87E-05 |
| T cell activation (GO:0042110) | 221 | 10 | 7.44 | 3.88E-05 |
| regulation of neutrophil chemotaxis (GO:0090022) | 28 | 5 | 29.36 | 4.12E-05 |
| regulation of actomyosin structure organization (GO:0110020) | 87 | 7 | 13.23 | 4.40E-05 |

| | | | | |
|---|---|---|---|---|
| response to fatty acid (GO:0070542) | 87 | 7 | 13.23 | 4.40E-05 |
| positive regulation of blood vessel endothelial cell migration (GO:0043536) | 54 | 6 | 18.27 | 4.41E-05 |
| peptidyl-serine modification (GO:0018209) | 175 | 9 | 8.45 | 4.82E-05 |
| positive regulation of GTPase activity (GO:0043547) | 411 | 13 | 5.2 | 4.82E-05 |
| regulation of GTPase activity (GO:0043087) | 483 | 14 | 4.76 | 5.11E-05 |
| T cell differentiation (GO:0030217) | 130 | 8 | 10.12 | 5.18E-05 |
| tube morphogenesis (GO:0035239) | 634 | 16 | 4.15 | 5.24E-05 |
| regulation of alpha-beta T cell activation (GO:0046634) | 90 | 7 | 12.79 | 5.34E-05 |
| regulation of JUN kinase activity (GO:0043506) | 90 | 7 | 12.79 | 5.35E-05 |
| secretion by cell (GO:0032940) | 970 | 20 | 3.39 | 5.35E-05 |
| response to vitamin (GO:0033273) | 90 | 7 | 12.79 | 5.36E-05 |
| regulation of peptidase activity (GO:0052547) | 417 | 13 | 5.12 | 5.55E-05 |
| positive regulation of endothelial cell proliferation (GO:0001938) | 91 | 7 | 12.65 | 5.66E-05 |
| complement activation (GO:0006956) | 179 | 9 | 8.27 | 5.66E-05 |
| extrinsic apoptotic signaling pathway (GO:0097191) | 92 | 7 | 12.51 | 6.05E-05 |
| positive regulation of interleukin-1 secretion (GO:0050716) | 31 | 5 | 26.51 | 6.22E-05 |
| response to vitamin D (GO:0033280) | 31 | 5 | 26.51 | 6.23E-05 |
| regulation of actin filament bundle assembly (GO:0032231) | 93 | 7 | 12.37 | 6.45E-05 |
| myeloid leukocyte activation (GO:0002274) | 569 | 15 | 4.33 | 6.51E-05 |
| cytoplasmic pattern recognition receptor signaling pathway (GO:0002753) | 32 | 5 | 25.69 | 7.10E-05 |
| regulation of vascular endothelial growth factor production (GO:0010574) | 32 | 5 | 25.69 | 7.11E-05 |
| regulation of cell development (GO:0060284) | 903 | 19 | 3.46 | 7.22E-05 |
| regulation of viral life cycle (GO:1903900) | 137 | 8 | 9.6 | 7.24E-05 |
| negative regulation of cellular component movement (GO:0051271) | 298 | 11 | 6.07 | 7.25E-05 |
| alpha-beta T cell activation (GO:0046631) | 60 | 6 | 16.44 | 7.38E-05 |
| positive regulation of endothelial cell migration (GO:0010595) | 96 | 7 | 11.99 | 7.71E-05 |
| negative regulation of production of molecular mediator of immune response (GO:0002701) | 33 | 5 | 24.91 | 8.01E-05 |
| response to prostaglandin (GO:0034694) | 33 | 5 | 24.91 | 8.02E-05 |
| regulation of alpha-beta T cell differentiation (GO:0046637) | 61 | 6 | 16.17 | 8.02E-05 |
| regulation of leukocyte mediated immunity (GO:0002703) | 189 | 9 | 7.83 | 8.32E-05 |

| | | | | |
|---|---|---|---|---|
| Fc receptor signaling pathway (GO:0038093) | 189 | 9 | 7.83 | 8.33E-05 |
| positive regulation of actin filament bundle assembly (GO:0032233) | 62 | 6 | 15.91 | 8.69E-05 |
| negative regulation of immune response (GO:0050777) | 141 | 8 | 9.33 | 8.71E-05 |
| regulation of cellular catabolic process (GO:0031329) | 747 | 17 | 3.74 | 9.03E-05 |
| regulation of T-helper cell differentiation (GO:0045622) | 34 | 5 | 24.18 | 9.04E-05 |
| negative regulation of neuron apoptotic process (GO:0043524) | 142 | 8 | 9.26 | 9.09E-05 |
| transmembrane receptor protein serine/threonine kinase signaling pathway (GO:0007178) | 192 | 9 | 7.71 | 9.32E-05 |
| negative regulation of neuron death (GO:1901215) | 193 | 9 | 7.67 | 9.69E-05 |
| angiogenesis (GO:0001525) | 309 | 11 | 5.85 | 9.88E-05 |
| response to fluid shear stress (GO:0034405) | 35 | 5 | 23.48 | 1.02E-04 |
| positive regulation of cysteine-type endopeptidase activity (GO:2001056) | 145 | 8 | 9.07 | 1.05E-04 |
| negative regulation of locomotion (GO:0040013) | 312 | 11 | 5.8 | 1.07E-04 |
| regulation of osteoclast differentiation (GO:0045670) | 65 | 6 | 15.17 | 1.09E-04 |
| cranial skeletal system development (GO:1904888) | 65 | 6 | 15.17 | 1.09E-04 |
| regulation of lymphocyte mediated immunity (GO:0002706) | 146 | 8 | 9.01 | 1.09E-04 |
| cellular response to peptide hormone stimulus (GO:0071375) | 252 | 10 | 6.52 | 1.09E-04 |
| response to oxidative stress (GO:0006979) | 378 | 12 | 5.22 | 1.09E-04 |
| positive regulation of macrophage migration (GO:1905523) | 15 | 4 | 43.84 | 1.11E-04 |
| response to laminar fluid shear stress (GO:0034616) | 15 | 4 | 43.84 | 1.11E-04 |
| regulation of interleukin-1 beta secretion (GO:0050706) | 36 | 5 | 22.83 | 1.13E-04 |
| regulation of endocytosis (GO:0030100) | 254 | 10 | 6.47 | 1.15E-04 |
| positive regulation of lymphocyte mediated immunity (GO:0002708) | 104 | 7 | 11.06 | 1.21E-04 |
| macrophage activation (GO:0042116) | 37 | 5 | 22.21 | 1.27E-04 |
| regulation of phosphatidylinositol 3-kinase signaling (GO:0014066) | 105 | 7 | 10.96 | 1.28E-04 |
| detection of other organism (GO:0098543) | 16 | 4 | 41.1 | 1.36E-04 |
| positive regulation of vascular endothelial growth factor receptor signaling pathway (GO:0030949) | 16 | 4 | 41.1 | 1.36E-04 |
| cytokine biosynthetic process (GO:0042089) | 16 | 4 | 41.1 | 1.36E-04 |
| regulation of growth (GO:0040008) | 690 | 16 | 3.81 | 1.37E-04 |
| response to progesterone (GO:0032570) | 38 | 5 | 21.63 | 1.42E-04 |

| | | | | |
|---|---|---|---|---|
| regulation of catabolic process (GO:0009894) | 863 | 18 | 3.43 | 1.42E-04 |
| protein activation cascade (GO:0072376) | 204 | 9 | 7.25 | 1.43E-04 |
| regulation of endopeptidase activity (GO:0052548) | 390 | 12 | 5.06 | 1.44E-04 |
| anatomical structure formation involved in morphogenesis (GO:0048646) | 866 | 18 | 3.42 | 1.48E-04 |
| regulation of neurogenesis (GO:0050767) | 783 | 17 | 3.57 | 1.57E-04 |
| cytokine metabolic process (GO:0042107) | 17 | 4 | 38.68 | 1.65E-04 |
| negative regulation of phosphate metabolic process (GO:0045936) | 542 | 14 | 4.25 | 1.65E-04 |
| negative regulation of phosphorus metabolic process (GO:0010563) | 543 | 14 | 4.24 | 1.68E-04 |
| positive regulation of phosphatidylinositol 3-kinase signaling (GO:0014068) | 71 | 6 | 13.89 | 1.69E-04 |
| regulation of defense response to virus by host (GO:0050691) | 40 | 5 | 20.55 | 1.75E-04 |
| regulation of epithelial cell apoptotic process (GO:1904035) | 72 | 6 | 13.7 | 1.81E-04 |
| cardiac ventricle morphogenesis (GO:0003208) | 72 | 6 | 13.7 | 1.81E-04 |
| response to steroid hormone (GO:0048545) | 334 | 11 | 5.41 | 1.89E-04 |
| activation of JUN kinase activity (GO:0007257) | 41 | 5 | 20.05 | 1.94E-04 |
| positive regulation of endothelial cell apoptotic process (GO:2000353) | 18 | 4 | 36.53 | 1.98E-04 |
| regulation of vascular endothelial cell proliferation (GO:1905562) | 18 | 4 | 36.53 | 1.98E-04 |
| regulation of extrinsic apoptotic signaling pathway (GO:2001236) | 161 | 8 | 8.17 | 2.04E-04 |
| complement activation, classical pathway (GO:0006958) | 162 | 8 | 8.12 | 2.13E-04 |
| JAK-STAT cascade (GO:0007259) | 42 | 5 | 19.57 | 2.14E-04 |
| STAT cascade (GO:0097696) | 42 | 5 | 19.57 | 2.14E-04 |
| response to acid chemical (GO:0001101) | 339 | 11 | 5.33 | 2.14E-04 |
| positive regulation of cytoskeleton organization (GO:0051495) | 216 | 9 | 6.85 | 2.15E-04 |
| microglial cell activation (GO:0001774) | 19 | 4 | 34.61 | 2.35E-04 |
| T-helper 1 type immune response (GO:0042088) | 19 | 4 | 34.61 | 2.35E-04 |
| leukocyte activation involved in inflammatory response (GO:0002269) | 19 | 4 | 34.61 | 2.36E-04 |
| positive regulation of response to biotic stimulus (GO:0002833) | 43 | 5 | 19.12 | 2.36E-04 |
| mononuclear cell proliferation (GO:0032943) | 76 | 6 | 12.98 | 2.37E-04 |
| positive regulation of calcium ion transport (GO:0051928) | 117 | 7 | 9.84 | 2.38E-04 |
| regulation of interleukin-1 secretion (GO:0050704) | 44 | 5 | 18.68 | 2.60E-04 |
| positive regulation of T-helper 1 type immune response (GO:0002827) | 20 | 4 | 32.88 | 2.76E-04 |
| response to muramyl dipeptide (GO:0032495) | 20 | 4 | 32.88 | 2.77E-04 |

| | | | | |
|---|---|---|---|---|
| cytokine secretion (GO:0050663) | 45 | 5 | 18.27 | 2.84E-04 |
| positive regulation of alpha-beta T cell differentiation (GO:0046638) | 45 | 5 | 18.27 | 2.84E-04 |
| negative regulation of smooth muscle cell proliferation (GO:0048662) | 45 | 5 | 18.27 | 2.85E-04 |
| regulation of stress fiber assembly (GO:0051492) | 79 | 6 | 12.49 | 2.86E-04 |
| Fc-epsilon receptor signaling pathway (GO:0038095) | 121 | 7 | 9.51 | 2.87E-04 |
| lymphocyte differentiation (GO:0030098) | 227 | 9 | 6.52 | 3.06E-04 |
| positive regulation of epithelial to mesenchymal transition (GO:0010718) | 46 | 5 | 17.87 | 3.10E-04 |
| positive regulation of cytokine-mediated signaling pathway (GO:0001961) | 46 | 5 | 17.87 | 3.11E-04 |
| regulation of CD4-positive, alpha-beta T cell differentiation (GO:0043370) | 46 | 5 | 17.87 | 3.11E-04 |
| T cell receptor signaling pathway (GO:0050852) | 123 | 7 | 9.36 | 3.15E-04 |
| positive regulation of T-helper cell differentiation (GO:0045624) | 21 | 4 | 31.31 | 3.22E-04 |
| regulation of lymphocyte chemotaxis (GO:1901623) | 21 | 4 | 31.31 | 3.22E-04 |
| positive regulation of leukocyte mediated immunity (GO:0002705) | 124 | 7 | 9.28 | 3.30E-04 |
| regulation of epithelial to mesenchymal transition (GO:0010717) | 82 | 6 | 12.03 | 3.44E-04 |
| cellular response to reactive oxygen species (GO:0034614) | 125 | 7 | 9.21 | 3.46E-04 |
| response to radiation (GO:0009314) | 432 | 12 | 4.57 | 3.61E-04 |
| regulation of wound healing (GO:0061041) | 126 | 7 | 9.13 | 3.62E-04 |
| positive regulation of cell division (GO:0051781) | 83 | 6 | 11.88 | 3.65E-04 |
| response to hypoxia (GO:0001666) | 295 | 10 | 5.57 | 3.67E-04 |
| regulation of biomineral tissue development (GO:0070167) | 84 | 6 | 11.74 | 3.86E-04 |
| regulation of binding (GO:0051098) | 365 | 11 | 4.95 | 3.94E-04 |
| positive regulation of production of molecular mediator of immune response (GO:0002702) | 85 | 6 | 11.6 | 4.10E-04 |
| positive regulation of cysteine-type endopeptidase activity involved in apoptotic process (GO:0043280) | 129 | 7 | 8.92 | 4.13E-04 |
| regulation of calcium ion transport (GO:0051924) | 238 | 9 | 6.22 | 4.26E-04 |
| cellular response to prostaglandin stimulus (GO:0071379) | 23 | 4 | 28.59 | 4.31E-04 |
| positive regulation of myoblast differentiation (GO:0045663) | 23 | 4 | 28.59 | 4.32E-04 |
| regulation of viral genome replication (GO:0045069) | 86 | 6 | 11.47 | 4.34E-04 |
| alpha-beta T cell differentiation (GO:0046632) | 50 | 5 | 16.44 | 4.37E-04 |
| regulation of morphogenesis of an epithelium (GO:1905330) | 131 | 7 | 8.78 | 4.50E-04 |

| | | | | |
|---|---|---|---|---|
| antigen receptor-mediated signaling pathway (GO:0050851) | 240 | 9 | 6.16 | 4.50E-04 |
| positive regulation of cell development (GO:0010720) | 520 | 13 | 4.11 | 4.55E-04 |
| response to decreased oxygen levels (GO:0036293) | 305 | 10 | 5.39 | 4.73E-04 |
| positive regulation of stress fiber assembly (GO:0051496) | 51 | 5 | 16.12 | 4.74E-04 |
| regulation of peptidyl-tyrosine phosphorylation (GO:0050730) | 242 | 9 | 6.11 | 4.76E-04 |
| heart morphogenesis (GO:0003007) | 242 | 9 | 6.11 | 4.77E-04 |
| negative regulation of biomineral tissue development (GO:0070168) | 24 | 4 | 27.4 | 4.93E-04 |
| TRIF-dependent toll-like receptor signaling pathway (GO:0035666) | 24 | 4 | 27.4 | 4.93E-04 |
| positive regulation of osteoclast differentiation (GO:0045672) | 24 | 4 | 27.4 | 4.94E-04 |
| sensory organ development (GO:0007423) | 525 | 13 | 4.07 | 4.96E-04 |
| regulation of lymphocyte migration (GO:2000401) | 52 | 5 | 15.81 | 5.12E-04 |
| positive regulation of phagocytosis (GO:0050766) | 52 | 5 | 15.81 | 5.12E-04 |
| positive regulation of neurogenesis (GO:0050769) | 450 | 12 | 4.38 | 5.13E-04 |
| regulation of interleukin-2 production (GO:0032663) | 53 | 5 | 15.51 | 5.53E-04 |
| response to prostaglandin E (GO:0034695) | 25 | 4 | 26.3 | 5.61E-04 |
| negative regulation of cell cycle (GO:0045786) | 532 | 13 | 4.02 | 5.61E-04 |
| negative regulation of transport (GO:0051051) | 456 | 12 | 4.33 | 5.75E-04 |
| negative regulation of viral genome replication (GO:0045071) | 54 | 5 | 15.22 | 5.98E-04 |
| regulation of nervous system development (GO:0051960) | 883 | 17 | 3.16 | 6.30E-04 |
| MyD88-independent toll-like receptor signaling pathway (GO:0002756) | 26 | 4 | 25.29 | 6.36E-04 |
| posttranscriptional regulation of gene expression (GO:0010608) | 461 | 12 | 4.28 | 6.36E-04 |
| neuroinflammatory response (GO:0150076) | 26 | 4 | 25.29 | 6.37E-04 |
| regulation of hormone biosynthetic process (GO:0046885) | 26 | 4 | 25.29 | 6.38E-04 |
| glial cell activation (GO:0061900) | 26 | 4 | 25.29 | 6.39E-04 |
| regulation of interleukin-17 production (GO:0032660) | 26 | 4 | 25.29 | 6.40E-04 |
| regulation of monooxygenase activity (GO:0032768) | 55 | 5 | 14.94 | 6.42E-04 |
| positive regulation of protein complex assembly (GO:0031334) | 254 | 9 | 5.82 | 6.64E-04 |
| positive regulation of supramolecular fiber organization (GO:1902905) | 195 | 8 | 6.74 | 6.82E-04 |
| regulation of CD4-positive, alpha-beta T cell activation (GO:2000514) | 56 | 5 | 14.68 | 6.93E-04 |
| regulation of T-helper 1 type immune response (GO:0002825) | 27 | 4 | 24.35 | 7.14E-04 |

| | | | | |
|---|---|---|---|---|
| nucleotide-binding oligomerization domain containing signaling pathway (GO:0070423) | 27 | 4 | 24.35 | 7.15E-04 |
| positive regulation of cAMP-mediated signaling (GO:0043950) | 27 | 4 | 24.35 | 7.16E-04 |
| response to angiotensin (GO:1990776) | 27 | 4 | 24.35 | 7.17E-04 |
| inflammatory response to antigenic stimulus (GO:0002437) | 27 | 4 | 24.35 | 7.18E-04 |
| negative regulation of myoblast differentiation (GO:0045662) | 27 | 4 | 24.35 | 7.19E-04 |
| regulation of cellular response to growth factor stimulus (GO:0090287) | 258 | 9 | 5.73 | 7.35E-04 |
| cellular response to fatty acid (GO:0071398) | 57 | 5 | 14.42 | 7.39E-04 |
| regulation of T cell cytokine production (GO:0002724) | 28 | 4 | 23.48 | 8.07E-04 |
| nucleotide-binding domain, leucine rich repeat containing receptor signaling pathway (GO:0035872) | 28 | 4 | 23.48 | 8.08E-04 |
| positive regulation of monooxygenase activity (GO:0032770) | 28 | 4 | 23.48 | 8.09E-04 |
| response to oxygen levels (GO:0070482) | 328 | 10 | 5.01 | 8.10E-04 |
| regulation of actin filament organization (GO:0110053) | 262 | 9 | 5.65 | 8.18E-04 |
| modification of morphology or physiology of other organism (GO:0035821) | 147 | 7 | 7.83 | 8.52E-04 |
| protein secretion (GO:0009306) | 148 | 7 | 7.78 | 8.86E-04 |
| positive regulation of CD4-positive, alpha-beta T cell differentiation (GO:0043372) | 29 | 4 | 22.67 | 9.06E-04 |
| positive regulation of erythrocyte differentiation (GO:0045648) | 29 | 4 | 22.67 | 9.07E-04 |
| salivary gland morphogenesis (GO:0007435) | 29 | 4 | 22.67 | 9.08E-04 |
| mesenchymal cell differentiation (GO:0048762) | 149 | 7 | 7.72 | 9.16E-04 |
| regulation of protein kinase B signaling (GO:0051896) | 205 | 8 | 6.42 | 9.27E-04 |
| regulation of response to wounding (GO:1903034) | 150 | 7 | 7.67 | 9.52E-04 |
| myeloid cell differentiation (GO:0030099) | 206 | 8 | 6.38 | 9.56E-04 |
| positive regulation of alpha-beta T cell activation (GO:0046635) | 61 | 5 | 13.47 | 9.79E-04 |
| positive regulation of gliogenesis (GO:0014015) | 61 | 5 | 13.47 | 9.80E-04 |
| modification of morphology or physiology of other organism involved in symbiotic interaction (GO:0051817) | 102 | 6 | 9.67 | 9.88E-04 |
| positive regulation of cellular catabolic process (GO:0031331) | 337 | 10 | 4.88 | 9.90E-04 |
| skeletal system development (GO:0001501) | 486 | 12 | 4.06 | 9.91E-04 |
| regulation of vascular endothelial growth factor receptor signaling pathway (GO:0030947) | 30 | 4 | 21.92 | 1.00E-03 |

| | | | | |
|---|---|---|---|---|
| positive regulation of cardiac muscle hypertrophy (GO:0010613) | 30 | 4 | 21.92 | 1.00E-03 |
| positive regulation of epithelial cell apoptotic process (GO:1904037) | 30 | 4 | 21.92 | 1.00E-03 |
| positive regulation of muscle hypertrophy (GO:0014742) | 30 | 4 | 21.92 | 1.00E-03 |
| positive regulation of interleukin-2 production (GO:0032743) | 30 | 4 | 21.92 | 1.01E-03 |
| regulation of interleukin-4 production (GO:0032673) | 30 | 4 | 21.92 | 1.01E-03 |
| cell cycle arrest (GO:0007050) | 152 | 7 | 7.57 | 1.02E-03 |
| regulation of pathway-restricted SMAD protein phosphorylation (GO:0060393) | 62 | 5 | 13.26 | 1.04E-03 |
| response to unfolded protein (GO:0006986) | 153 | 7 | 7.52 | 1.05E-03 |
| regulation of ion transport (GO:0043269) | 657 | 14 | 3.5 | 1.09E-03 |
| negative regulation of intracellular signal transduction (GO:1902532) | 494 | 12 | 3.99 | 1.14E-03 |
| regulation of supramolecular fiber organization (GO:1902903) | 346 | 10 | 4.75 | 1.20E-03 |
| positive regulation of lymphocyte migration (GO:2000403) | 32 | 4 | 20.55 | 1.24E-03 |
| regulation of superoxide metabolic process (GO:0090322) | 32 | 4 | 20.55 | 1.24E-03 |
| negative regulation of blood vessel endothelial cell migration (GO:0043537) | 32 | 4 | 20.55 | 1.24E-03 |
| interaction with host (GO:0051701) | 158 | 7 | 7.28 | 1.26E-03 |
| regulation of osteoblast differentiation (GO:0045667) | 108 | 6 | 9.13 | 1.29E-03 |
| positive regulation of protein kinase B signaling (GO:0051897) | 159 | 7 | 7.24 | 1.30E-03 |
| striated muscle tissue development (GO:0014706) | 281 | 9 | 5.27 | 1.31E-03 |
| cardiac muscle tissue development (GO:0048738) | 160 | 7 | 7.19 | 1.35E-03 |
| embryonic organ development (GO:0048568) | 426 | 11 | 4.24 | 1.35E-03 |
| positive regulation of CD4-positive, alpha-beta T cell activation (GO:2000516) | 33 | 4 | 19.93 | 1.36E-03 |
| cellular response to interleukin-6 (GO:0071354) | 33 | 4 | 19.93 | 1.36E-03 |
| positive regulation of vascular smooth muscle cell proliferation (GO:1904707) | 33 | 4 | 19.93 | 1.36E-03 |
| salivary gland development (GO:0007431) | 33 | 4 | 19.93 | 1.37E-03 |
| response to hydrogen peroxide (GO:0042542) | 111 | 6 | 8.89 | 1.47E-03 |
| negative regulation of catalytic activity (GO:0043086) | 768 | 15 | 3.21 | 1.47E-03 |
| cell proliferation (GO:0008283) | 679 | 14 | 3.39 | 1.48E-03 |
| positive regulation of phosphatidylinositol 3-kinase activity (GO:0043552) | 34 | 4 | 19.34 | 1.49E-03 |
| connective tissue development (GO:0061448) | 222 | 8 | 5.92 | 1.49E-03 |
| peptidyl-threonine phosphorylation (GO:0018107) | 68 | 5 | 12.09 | 1.50E-03 |
| regulation of bone mineralization (GO:0030500) | 68 | 5 | 12.09 | 1.50E-03 |

| | | | | |
|---|---|---|---|---|
| positive regulation of nervous system development (GO:0051962) | 514 | 12 | 3.84 | 1.58E-03 |
| peptide secretion (GO:0002790) | 165 | 7 | 6.97 | 1.59E-03 |
| ER-nucleus signaling pathway (GO:0006984) | 35 | 4 | 18.79 | 1.65E-03 |
| cellular response to antibiotic (GO:0071236) | 114 | 6 | 8.65 | 1.67E-03 |
| cellular response to oxidative stress (GO:0034599) | 226 | 8 | 5.82 | 1.67E-03 |
| positive regulation of lipase activity (GO:0060193) | 70 | 5 | 11.74 | 1.69E-03 |
| muscle tissue development (GO:0060537) | 294 | 9 | 5.03 | 1.76E-03 |
| negative regulation of extrinsic apoptotic signaling pathway via death domain receptors (GO:1902042) | 36 | 4 | 18.27 | 1.79E-03 |
| outflow tract morphogenesis (GO:0003151) | 71 | 5 | 11.58 | 1.79E-03 |
| negative regulation of immune effector process (GO:0002698) | 116 | 6 | 8.5 | 1.80E-03 |
| regulation of sequestering of calcium ion (GO:0051282) | 117 | 6 | 8.43 | 1.88E-03 |
| muscle organ development (GO:0007517) | 297 | 9 | 4.98 | 1.88E-03 |
| regulation of defense response to virus (GO:0050688) | 72 | 5 | 11.42 | 1.89E-03 |
| regulation of metal ion transport (GO:0010959) | 370 | 10 | 4.44 | 1.94E-03 |
| response to endoplasmic reticulum stress (GO:0034976) | 232 | 8 | 5.67 | 1.95E-03 |
| response to interleukin-6 (GO:0070741) | 37 | 4 | 17.77 | 1.96E-03 |
| positive regulation of lipid kinase activity (GO:0090218) | 37 | 4 | 17.77 | 1.96E-03 |
| regulation of hormone metabolic process (GO:0032350) | 37 | 4 | 17.77 | 1.96E-03 |
| response to topologically incorrect protein (GO:0035966) | 172 | 7 | 6.69 | 1.98E-03 |
| regulation of cysteine-type endopeptidase activity (GO:2000116) | 234 | 8 | 5.62 | 2.04E-03 |
| lymphocyte proliferation (GO:0046651) | 74 | 5 | 11.11 | 2.10E-03 |
| positive regulation of B cell mediated immunity (GO:0002714) | 38 | 4 | 17.3 | 2.12E-03 |
| negative regulation of toll-like receptor signaling pathway (GO:0034122) | 38 | 4 | 17.3 | 2.13E-03 |
| positive regulation of immunoglobulin mediated immune response (GO:0002891) | 38 | 4 | 17.3 | 2.13E-03 |
| peptidyl-threonine modification (GO:0018210) | 75 | 5 | 10.96 | 2.21E-03 |
| regulation of cellular response to oxidative stress (GO:1900407) | 75 | 5 | 10.96 | 2.21E-03 |
| response to osmotic stress (GO:0006970) | 75 | 5 | 10.96 | 2.22E-03 |
| regulation of insulin secretion (GO:0050796) | 176 | 7 | 6.54 | 2.23E-03 |
| positive regulation of cytokine production involved in immune response (GO:0002720) | 39 | 4 | 16.86 | 2.31E-03 |

| | | | | |
|---|---|---|---|---|
| T cell selection (GO:0045058) | 39 | 4 | 16.86 | 2.32E-03 |
| regulation of tissue remodeling (GO:0034103) | 76 | 5 | 10.82 | 2.33E-03 |
| regulation of reactive oxygen species biosynthetic process (GO:1903426) | 76 | 5 | 10.82 | 2.33E-03 |
| negative regulation of response to external stimulus (GO:0032102) | 307 | 9 | 4.82 | 2.33E-03 |
| regulation of transmembrane transport (GO:0034762) | 540 | 12 | 3.65 | 2.35E-03 |
| digestive tract development (GO:0048565) | 124 | 6 | 7.95 | 2.46E-03 |
| cellular response to vascular endothelial growth factor stimulus (GO:0035924) | 40 | 4 | 16.44 | 2.50E-03 |
| negative regulation of leukocyte migration (GO:0002686) | 40 | 4 | 16.44 | 2.50E-03 |
| positive regulation of DNA replication (GO:0045740) | 40 | 4 | 16.44 | 2.51E-03 |
| cardiac ventricle development (GO:0003231) | 125 | 6 | 7.89 | 2.55E-03 |
| Ras protein signal transduction (GO:0007265) | 243 | 8 | 5.41 | 2.55E-03 |
| regulation of hemostasis (GO:1900046) | 78 | 5 | 10.54 | 2.58E-03 |
| negative regulation of viral life cycle (GO:1903901) | 78 | 5 | 10.54 | 2.58E-03 |
| regulation of phospholipid metabolic process (GO:1903725) | 78 | 5 | 10.54 | 2.58E-03 |
| regulation of blood coagulation (GO:0030193) | 78 | 5 | 10.54 | 2.58E-03 |
| response to starvation (GO:0042594) | 181 | 7 | 6.36 | 2.58E-03 |
| regulation of cellular amide metabolic process (GO:0034248) | 386 | 10 | 4.26 | 2.59E-03 |
| positive regulation of peptidyl-tyrosine phosphorylation (GO:0050731) | 182 | 7 | 6.32 | 2.66E-03 |
| negative regulation of growth of symbiont in host (GO:0044130) | 15 | 3 | 32.88 | 2.83E-03 |
| detection of bacterium (GO:0016045) | 15 | 3 | 32.88 | 2.83E-03 |
| negative regulation of cell activation (GO:0050866) | 184 | 7 | 6.25 | 2.83E-03 |
| positive regulation of interleukin-17 production (GO:0032740) | 15 | 3 | 32.88 | 2.84E-03 |
| interleukin-1 production (GO:0032612) | 15 | 3 | 32.88 | 2.84E-03 |
| ephrin receptor signaling pathway (GO:0048013) | 80 | 5 | 10.27 | 2.84E-03 |
| cardiac chamber morphogenesis (GO:0003206) | 128 | 6 | 7.71 | 2.84E-03 |
| negative regulation of leukocyte proliferation (GO:0070664) | 80 | 5 | 10.27 | 2.85E-03 |
| regulation of DNA-templated transcription in response to stress (GO:0043620) | 80 | 5 | 10.27 | 2.85E-03 |
| regulation of epidermis development (GO:0045682) | 81 | 5 | 10.15 | 3.00E-03 |
| exocrine system development (GO:0035272) | 43 | 4 | 15.29 | 3.16E-03 |
| regulation of coagulation (GO:0050818) | 82 | 5 | 10.02 | 3.16E-03 |
| positive regulation of ossification (GO:0045778) | 82 | 5 | 10.02 | 3.17E-03 |

| | | | | |
|---|---|---|---|---|
| regulation of growth of symbiont in host (GO:0044126) | 16 | 3 | 30.82 | 3.30E-03 |
| negative regulation of growth of symbiont involved in interaction with host (GO:0044146) | 16 | 3 | 30.82 | 3.31E-03 |
| response to ammonium ion (GO:0060359) | 133 | 6 | 7.42 | 3.42E-03 |
| regulation of response to oxidative stress (GO:1902882) | 84 | 5 | 9.79 | 3.49E-03 |
| negative regulation of growth (GO:0045926) | 257 | 8 | 5.12 | 3.59E-03 |
| negative regulation of protein modification process (GO:0031400) | 569 | 12 | 3.47 | 3.61E-03 |
| regulation of cell junction assembly (GO:1901888) | 85 | 5 | 9.67 | 3.63E-03 |
| response to dsRNA (GO:0043331) | 85 | 5 | 9.67 | 3.63E-03 |
| digestive system development (GO:0055123) | 135 | 6 | 7.31 | 3.63E-03 |
| bone development (GO:0060348) | 193 | 7 | 5.96 | 3.63E-03 |
| positive regulation of catabolic process (GO:0009896) | 405 | 10 | 4.06 | 3.63E-03 |
| peptidyl-amino acid modification (GO:0018193) | 845 | 15 | 2.92 | 3.66E-03 |
| modulation of growth of symbiont involved in interaction with host (GO:0044144) | 17 | 3 | 29.01 | 3.77E-03 |
| regulation of immunoglobulin secretion (GO:0051023) | 17 | 3 | 29.01 | 3.77E-03 |
| cellular response to interferon-beta (GO:0035458) | 17 | 3 | 29.01 | 3.77E-03 |
| regulation of leukocyte apoptotic process (GO:2000106) | 86 | 5 | 9.56 | 3.77E-03 |
| positive regulation of viral process (GO:0048524) | 86 | 5 | 9.56 | 3.77E-03 |
| regulation of glucose transmembrane transport (GO:0010827) | 86 | 5 | 9.56 | 3.77E-03 |
| toll-like receptor 4 signaling pathway (GO:0034142) | 17 | 3 | 29.01 | 3.78E-03 |
| cellular response to prostaglandin E stimulus (GO:0071380) | 17 | 3 | 29.01 | 3.78E-03 |
| positive regulation of animal organ morphogenesis (GO:0110110) | 86 | 5 | 9.56 | 3.78E-03 |
| regulation of vesicle-mediated transport (GO:0060627) | 488 | 11 | 3.71 | 3.78E-03 |
| positive regulation of ion transport (GO:0043270) | 261 | 8 | 5.04 | 3.86E-03 |
| embryonic cranial skeleton morphogenesis (GO:0048701) | 46 | 4 | 14.29 | 3.87E-03 |
| response to exogenous dsRNA (GO:0043330) | 46 | 4 | 14.29 | 3.87E-03 |
| positive regulation of lipid metabolic process (GO:0045834) | 137 | 6 | 7.2 | 3.87E-03 |
| regulation of interleukin-10 production (GO:0032653) | 46 | 4 | 14.29 | 3.88E-03 |
| gliogenesis (GO:0042063) | 196 | 7 | 5.87 | 3.89E-03 |
| cellular response to toxic substance (GO:0097237) | 196 | 7 | 5.87 | 3.90E-03 |
| aging (GO:0007568) | 262 | 8 | 5.02 | 3.93E-03 |

| | | | | |
|---|---|---|---|---|
| positive regulation of cellular amide metabolic process (GO:0034250) | 138 | 6 | 7.15 | 3.98E-03 |
| positive regulation of endocytosis (GO:0045807) | 138 | 6 | 7.15 | 3.99E-03 |
| cellular response to metal ion (GO:0071248) | 198 | 7 | 5.81 | 4.12E-03 |
| gland development (GO:0048732) | 413 | 10 | 3.98 | 4.13E-03 |
| regulation of erythrocyte differentiation (GO:0045646) | 47 | 4 | 13.99 | 4.14E-03 |
| lymph node development (GO:0048535) | 18 | 3 | 27.4 | 4.27E-03 |
| membrane protein intracellular domain proteolysis (GO:0031293) | 18 | 3 | 27.4 | 4.27E-03 |
| negative regulation of lymphocyte activation (GO:0051250) | 140 | 6 | 7.05 | 4.27E-03 |
| G-protein coupled acetylcholine receptor signaling pathway (GO:0007213) | 18 | 3 | 27.4 | 4.28E-03 |
| regulation of interleukin-2 biosynthetic process (GO:0045076) | 18 | 3 | 27.4 | 4.28E-03 |
| positive regulation of monocyte chemotaxis (GO:0090026) | 18 | 3 | 27.4 | 4.28E-03 |
| dendritic cell chemotaxis (GO:0002407) | 18 | 3 | 27.4 | 4.29E-03 |
| positive regulation of pathway-restricted SMAD protein phosphorylation (GO:0010862) | 48 | 4 | 13.7 | 4.42E-03 |
| negative regulation of endothelial cell migration (GO:0010596) | 48 | 4 | 13.7 | 4.42E-03 |
| regulation of phosphatidylinositol 3-kinase activity (GO:0043551) | 48 | 4 | 13.7 | 4.43E-03 |
| ventricular cardiac muscle tissue morphogenesis (GO:0055010) | 48 | 4 | 13.7 | 4.43E-03 |
| visual system development (GO:0150063) | 341 | 9 | 4.34 | 4.52E-03 |
| eye development (GO:0001654) | 341 | 9 | 4.34 | 4.53E-03 |
| regulation of oxidoreductase activity (GO:0051341) | 91 | 5 | 9.03 | 4.69E-03 |
| cellular response to starvation (GO:0009267) | 143 | 6 | 6.9 | 4.69E-03 |
| positive regulation of reactive oxygen species metabolic process (GO:2000379) | 91 | 5 | 9.03 | 4.70E-03 |
| positive regulation of phospholipid metabolic process (GO:1903727) | 49 | 4 | 13.42 | 4.72E-03 |
| negative regulation of leukocyte apoptotic process (GO:2000107) | 49 | 4 | 13.42 | 4.73E-03 |
| positive regulation of T cell cytokine production (GO:0002726) | 19 | 3 | 25.96 | 4.83E-03 |
| negative regulation of glucose transmembrane transport (GO:0010829) | 19 | 3 | 25.96 | 4.84E-03 |
| positive regulation of glycoprotein biosynthetic process (GO:0010560) | 19 | 3 | 25.96 | 4.84E-03 |
| response to muscle stretch (GO:0035994) | 19 | 3 | 25.96 | 4.85E-03 |
| defense response to protozoan (GO:0042832) | 19 | 3 | 25.96 | 4.85E-03 |
| regulation of hair follicle development (GO:0051797) | 19 | 3 | 25.96 | 4.86E-03 |

| | | | | |
|---|---|---|---|---|
| activation of cysteine-type endopeptidase activity involved in apoptotic process (GO:0006919) | 92 | 5 | 8.93 | 4.88E-03 |
| exocytosis (GO:0006887) | 777 | 14 | 2.96 | 4.92E-03 |
| sensory system development (GO:0048880) | 346 | 9 | 4.28 | 4.95E-03 |
| positive regulation of oxidoreductase activity (GO:0051353) | 50 | 4 | 13.15 | 5.02E-03 |
| response to fungus (GO:0009620) | 50 | 4 | 13.15 | 5.02E-03 |
| positive regulation of wound healing (GO:0090303) | 50 | 4 | 13.15 | 5.03E-03 |
| regulation of peptide hormone secretion (GO:0090276) | 207 | 7 | 5.56 | 5.16E-03 |
| regulation of lipase activity (GO:0060191) | 94 | 5 | 8.74 | 5.32E-03 |
| cellular response to calcium ion (GO:0071277) | 94 | 5 | 8.74 | 5.32E-03 |
| regulation of cAMP-mediated signaling (GO:0043949) | 51 | 4 | 12.89 | 5.35E-03 |
| T cell costimulation (GO:0031295) | 51 | 4 | 12.89 | 5.35E-03 |
| leukocyte cell-cell adhesion (GO:0007159) | 51 | 4 | 12.89 | 5.36E-03 |
| lymphocyte homeostasis (GO:0002260) | 51 | 4 | 12.89 | 5.36E-03 |
| negative regulation of phosphorylation (GO:0042326) | 430 | 10 | 3.82 | 5.42E-03 |
| mRNA transcription by RNA polymerase II (GO:0042789) | 20 | 3 | 24.66 | 5.44E-03 |
| regulation of tumor necrosis factor biosynthetic process (GO:0042534) | 20 | 3 | 24.66 | 5.44E-03 |
| response to protozoan (GO:0001562) | 20 | 3 | 24.66 | 5.45E-03 |
| negative regulation of oxidative stress-induced intrinsic apoptotic signaling pathway (GO:1902176) | 20 | 3 | 24.66 | 5.45E-03 |
| negative regulation of catabolic process (GO:0009895) | 279 | 8 | 4.71 | 5.59E-03 |
| lymphocyte costimulation (GO:0031294) | 52 | 4 | 12.65 | 5.64E-03 |
| tissue remodeling (GO:0048771) | 96 | 5 | 8.56 | 5.71E-03 |
| positive regulation of plasma membrane bounded cell projection assembly (GO:0120034) | 96 | 5 | 8.56 | 5.71E-03 |
| regulation of cysteine-type endopeptidase activity involved in apoptotic process (GO:0043281) | 212 | 7 | 5.43 | 5.79E-03 |
| negative regulation of cellular protein metabolic process (GO:0032269) | 992 | 16 | 2.65 | 5.92E-03 |
| mesenchyme development (GO:0060485) | 213 | 7 | 5.4 | 5.94E-03 |
| regulation of B cell mediated immunity (GO:0002712) | 53 | 4 | 12.41 | 5.99E-03 |
| regulation of morphogenesis of a branching structure (GO:0060688) | 53 | 4 | 12.41 | 6.00E-03 |

| | | | | |
|---|---|---|---|---|
| regulation of immunoglobulin mediated immune response (GO:0002889) | 53 | 4 | 12.41 | 6.01E-03 |
| regulation of nuclease activity (GO:0032069) | 21 | 3 | 23.48 | 6.05E-03 |
| T cell migration (GO:0072678) | 21 | 3 | 23.48 | 6.05E-03 |
| positive regulation of glycoprotein metabolic process (GO:1903020) | 21 | 3 | 23.48 | 6.06E-03 |
| regulation of heterotypic cell-cell adhesion (GO:0034114) | 21 | 3 | 23.48 | 6.07E-03 |
| ventricular cardiac muscle tissue development (GO:0003229) | 54 | 4 | 12.18 | 6.38E-03 |
| gland morphogenesis (GO:0022612) | 99 | 5 | 8.3 | 6.45E-03 |
| endoplasmic reticulum unfolded protein response (GO:0030968) | 99 | 5 | 8.3 | 6.46E-03 |
| regulation of protein binding (GO:0043393) | 217 | 7 | 5.3 | 6.54E-03 |
| cellular response to nutrient levels (GO:0031669) | 217 | 7 | 5.3 | 6.55E-03 |
| cellular response to angiotensin (GO:1904385) | 22 | 3 | 22.42 | 6.77E-03 |
| response to gamma radiation (GO:0010332) | 55 | 4 | 11.96 | 6.77E-03 |
| regulation of SMAD protein signal transduction (GO:0060390) | 22 | 3 | 22.42 | 6.78E-03 |
| dendritic cell migration (GO:0036336) | 22 | 3 | 22.42 | 6.78E-03 |
| regulation of cardiac muscle hypertrophy (GO:0010611) | 55 | 4 | 11.96 | 6.78E-03 |
| positive regulation of toll-like receptor signaling pathway (GO:0034123) | 22 | 3 | 22.42 | 6.79E-03 |
| cell adhesion (GO:0007155) | 906 | 15 | 2.72 | 6.83E-03 |
| positive regulation of DNA metabolic process (GO:0051054) | 219 | 7 | 5.25 | 6.84E-03 |
| regulation of cell division (GO:0051302) | 157 | 6 | 6.28 | 7.12E-03 |
| positive regulation of cell projection organization (GO:0031346) | 367 | 9 | 4.03 | 7.12E-03 |
| cognition (GO:0050890) | 291 | 8 | 4.52 | 7.13E-03 |
| regulation of lipid kinase activity (GO:0043550) | 56 | 4 | 11.74 | 7.14E-03 |
| biological adhesion (GO:0022610) | 912 | 15 | 2.7 | 7.26E-03 |
| cellular response to inorganic substance (GO:0071241) | 222 | 7 | 5.18 | 7.36E-03 |
| embryo development (GO:0009790) | 914 | 15 | 2.7 | 7.41E-03 |
| regulation of inflammatory response to antigenic stimulus (GO:0002861) | 23 | 3 | 21.44 | 7.52E-03 |
| regulation of monocyte chemotaxis (GO:0090025) | 23 | 3 | 21.44 | 7.53E-03 |
| response to interferon-alpha (GO:0035455) | 23 | 3 | 21.44 | 7.53E-03 |
| positive regulation of transcription from RNA polymerase II promoter involved in cellular response to chemical stimulus (GO:1901522) | 23 | 3 | 21.44 | 7.54E-03 |
| regulation of muscle hypertrophy (GO:0014743) | 57 | 4 | 11.54 | 7.54E-03 |

| | | | | |
|---|---|---|---|---|
| regulation of system process (GO:0044057) | 538 | 11 | 3.36 | 7.54E-03 |
| positive regulation of response to wounding (GO:1903036) | 57 | 4 | 11.54 | 7.55E-03 |
| regulation of extrinsic apoptotic signaling pathway via death domain receptors (GO:1902041) | 57 | 4 | 11.54 | 7.55E-03 |
| regulation of cell-substrate junction assembly (GO:0090109) | 58 | 4 | 11.34 | 7.89E-03 |
| regulation of focal adhesion assembly (GO:0051893) | 58 | 4 | 11.34 | 7.90E-03 |
| response to metal ion (GO:0010038) | 375 | 9 | 3.95 | 8.05E-03 |
| regulation of cellular extravasation (GO:0002691) | 24 | 3 | 20.55 | 8.22E-03 |
| positive regulation of interleukin-4 production (GO:0032753) | 24 | 3 | 20.55 | 8.23E-03 |
| positive regulation of interleukin-6 secretion (GO:2000778) | 24 | 3 | 20.55 | 8.24E-03 |
| negative regulation of viral transcription (GO:0032897) | 24 | 3 | 20.55 | 8.24E-03 |
| regulation of protein complex assembly (GO:0043254) | 459 | 10 | 3.58 | 8.27E-03 |
| positive regulation of phospholipase activity (GO:0010518) | 59 | 4 | 11.15 | 8.31E-03 |
| positive regulation of viral life cycle (GO:1903902) | 59 | 4 | 11.15 | 8.32E-03 |
| regulation of muscle system process (GO:0090257) | 228 | 7 | 5.05 | 8.32E-03 |
| negative regulation of leukocyte activation (GO:0002695) | 164 | 6 | 6.01 | 8.55E-03 |
| negative regulation of T cell activation (GO:0050868) | 107 | 5 | 7.68 | 8.60E-03 |
| regulation of gliogenesis (GO:0014013) | 107 | 5 | 7.68 | 8.61E-03 |
| muscle structure development (GO:0061061) | 463 | 10 | 3.55 | 8.78E-03 |
| cardiac chamber development (GO:0003205) | 165 | 6 | 5.98 | 8.79E-03 |
| icosanoid metabolic process (GO:0006690) | 108 | 5 | 7.61 | 8.93E-03 |
| negative regulation of binding (GO:0051100) | 166 | 6 | 5.94 | 9.05E-03 |
| B cell homeostasis (GO:0001782) | 25 | 3 | 19.73 | 9.07E-03 |
| positive regulation of macroautophagy (GO:0016239) | 61 | 4 | 10.78 | 9.27E-03 |
| negative regulation of cell-cell adhesion (GO:0022408) | 167 | 6 | 5.91 | 9.27E-03 |
| negative regulation of cytokine secretion (GO:0050710) | 61 | 4 | 10.78 | 9.28E-03 |
| viral life cycle (GO:0019058) | 167 | 6 | 5.91 | 9.28E-03 |
| SMAD protein signal transduction (GO:0060395) | 61 | 4 | 10.78 | 9.29E-03 |
| regulation of cell-matrix adhesion (GO:0001952) | 110 | 5 | 7.47 | 9.59E-03 |
| regulation of smooth muscle cell migration (GO:0014910) | 62 | 4 | 10.61 | 9.78E-03 |
| T-helper cell differentiation (GO:0042093) | 26 | 3 | 18.97 | 9.91E-03 |

| | | | | |
|---|---|---|---|---|
| positive regulation of blood coagulation (GO:0030194) | 26 | 3 | 18.97 | 9.92E-03 |
| response to interferon-beta (GO:0035456) | 26 | 3 | 18.97 | 9.93E-03 |
| positive regulation of hemostasis (GO:1900048) | 26 | 3 | 18.97 | 9.93E-03 |
| small GTPase mediated signal transduction (GO:0007264) | 309 | 8 | 4.26 | 9.93E-03 |
| replacement ossification (GO:0036075) | 26 | 3 | 18.97 | 9.94E-03 |
| regulation of lipopolysaccharide-mediated signaling pathway (GO:0031664) | 26 | 3 | 18.97 | 9.95E-03 |
| positive regulation of T cell migration (GO:2000406) | 26 | 3 | 18.97 | 9.96E-03 |
| CD4-positive, alpha-beta T cell differentiation involved in immune response (GO:0002294) | 26 | 3 | 18.97 | 9.97E-03 |
| endochondral ossification (GO:0001958) | 26 | 3 | 18.97 | 9.98E-03 |
| cell cycle process (GO:0022402) | 947 | 15 | 2.6 | 1.00E-02 |
| regulation of immunoglobulin production (GO:0002637) | 63 | 4 | 10.44 | 1.02E-02 |
| regulation of adherens junction organization (GO:1903391) | 63 | 4 | 10.44 | 1.02E-02 |
| bone morphogenesis (GO:0060349) | 113 | 5 | 7.27 | 1.05E-02 |
| negative regulation of transmembrane transport (GO:0034763) | 113 | 5 | 7.27 | 1.05E-02 |
| response to acetylcholine (GO:1905144) | 27 | 3 | 18.27 | 1.06E-02 |
| mRNA transcription (GO:0009299) | 27 | 3 | 18.27 | 1.06E-02 |
| regulation of hair cycle (GO:0042634) | 27 | 3 | 18.27 | 1.06E-02 |
| signal transduction involved in cellular response to ammonium ion (GO:1903831) | 27 | 3 | 18.27 | 1.06E-02 |
| stimulatory C-type lectin receptor signaling pathway (GO:0002223) | 64 | 4 | 10.27 | 1.06E-02 |
| cellular response to hydrogen peroxide (GO:0070301) | 64 | 4 | 10.27 | 1.06E-02 |
| positive regulation of acute inflammatory response (GO:0002675) | 27 | 3 | 18.27 | 1.07E-02 |
| alpha-beta T cell differentiation involved in immune response (GO:0002293) | 27 | 3 | 18.27 | 1.07E-02 |
| alpha-beta T cell activation involved in immune response (GO:0002287) | 27 | 3 | 18.27 | 1.07E-02 |
| modulation by host of symbiont transcription (GO:0052472) | 27 | 3 | 18.27 | 1.07E-02 |
| acetylcholine receptor signaling pathway (GO:0095500) | 27 | 3 | 18.27 | 1.07E-02 |
| modulation by host of viral transcription (GO:0043921) | 27 | 3 | 18.27 | 1.07E-02 |
| positive regulation of coagulation (GO:0050820) | 27 | 3 | 18.27 | 1.07E-02 |
| positive regulation of cardiocyte differentiation (GO:1905209) | 27 | 3 | 18.27 | 1.07E-02 |
| cellular response to acetylcholine (GO:1905145) | 27 | 3 | 18.27 | 1.07E-02 |
| cartilage development (GO:0051216) | 173 | 6 | 5.7 | 1.07E-02 |

| | | | | |
|---|---|---|---|---|
| regulation of oxidative stress-induced cell death (GO:1903201) | 65 | 4 | 10.12 | 1.11E-02 |
| cellular response to ammonium ion (GO:0071242) | 65 | 4 | 10.12 | 1.11E-02 |
| positive regulation of tyrosine phosphorylation of STAT protein (GO:0042531) | 65 | 4 | 10.12 | 1.11E-02 |
| negative regulation of epithelial cell migration (GO:0010633) | 65 | 4 | 10.12 | 1.11E-02 |
| response to fibroblast growth factor (GO:0071774) | 115 | 5 | 7.15 | 1.11E-02 |
| regulation of transforming growth factor beta production (GO:0071634) | 28 | 3 | 17.61 | 1.16E-02 |
| modulation of transcription in other organism involved in symbiotic interaction (GO:0052312) | 28 | 3 | 17.61 | 1.16E-02 |
| regulation of myeloid cell apoptotic process (GO:0033032) | 28 | 3 | 17.61 | 1.16E-02 |
| positive regulation of histone acetylation (GO:0035066) | 28 | 3 | 17.61 | 1.16E-02 |
| epithelial to mesenchymal transition (GO:0001837) | 66 | 4 | 9.96 | 1.16E-02 |
| modification by host of symbiont morphology or physiology (GO:0051851) | 66 | 4 | 9.96 | 1.16E-02 |
| regulation of T cell mediated immunity (GO:0002709) | 66 | 4 | 9.96 | 1.16E-02 |
| neuron differentiation (GO:0030182) | 964 | 15 | 2.56 | 1.16E-02 |
| negative regulation of leukocyte cell-cell adhesion (GO:1903038) | 117 | 5 | 7.03 | 1.19E-02 |
| cardiocyte differentiation (GO:0035051) | 117 | 5 | 7.03 | 1.19E-02 |
| cellular response to extracellular stimulus (GO:0031668) | 246 | 7 | 4.68 | 1.20E-02 |
| innate immune response activating cell surface receptor signaling pathway (GO:0002220) | 67 | 4 | 9.81 | 1.22E-02 |
| leukocyte homeostasis (GO:0001776) | 67 | 4 | 9.81 | 1.22E-02 |
| regulation of developmental growth (GO:0048638) | 322 | 8 | 4.08 | 1.22E-02 |
| regulation of mRNA stability (GO:0043488) | 118 | 5 | 6.97 | 1.23E-02 |
| negative regulation of protein secretion (GO:0050709) | 119 | 5 | 6.91 | 1.27E-02 |
| regulation of DNA binding (GO:0051101) | 119 | 5 | 6.91 | 1.27E-02 |
| cellular response to unfolded protein (GO:0034620) | 119 | 5 | 6.91 | 1.27E-02 |
| ossification (GO:0001503) | 250 | 7 | 4.6 | 1.30E-02 |
| rhythmic process (GO:0048511) | 252 | 7 | 4.57 | 1.35E-02 |
| regulation of endothelial cell differentiation (GO:0045601) | 30 | 3 | 16.44 | 1.36E-02 |
| positive regulation of interleukin-10 production (GO:0032733) | 30 | 3 | 16.44 | 1.36E-02 |
| regulation of oxidative stress-induced intrinsic apoptotic signaling pathway (GO:1902175) | 30 | 3 | 16.44 | 1.36E-02 |

| | | | | |
|---|---|---|---|---|
| regulation of platelet activation (GO:0010543) | 30 | 3 | 16.44 | 1.36E-02 |
| regulation of cellular localization (GO:0060341) | 879 | 14 | 2.62 | 1.36E-02 |
| interaction with symbiont (GO:0051702) | 70 | 4 | 9.39 | 1.39E-02 |
| regulation of cofactor metabolic process (GO:0051193) | 70 | 4 | 9.39 | 1.39E-02 |
| cardiac muscle tissue morphogenesis (GO:0055008) | 70 | 4 | 9.39 | 1.39E-02 |
| regulation of phospholipase activity (GO:0010517) | 70 | 4 | 9.39 | 1.39E-02 |
| ERBB signaling pathway (GO:0038127) | 71 | 4 | 9.26 | 1.46E-02 |
| negative regulation of transforming growth factor beta receptor signaling pathway (GO:0030512) | 71 | 4 | 9.26 | 1.46E-02 |
| positive regulation of peptidyl-lysine acetylation (GO:2000758) | 31 | 3 | 15.91 | 1.47E-02 |
| leukotriene metabolic process (GO:0006691) | 31 | 3 | 15.91 | 1.47E-02 |
| T cell differentiation involved in immune response (GO:0002292) | 31 | 3 | 15.91 | 1.47E-02 |
| hyaluronan metabolic process (GO:0030212) | 31 | 3 | 15.91 | 1.47E-02 |
| dendritic cell differentiation (GO:0097028) | 31 | 3 | 15.91 | 1.47E-02 |
| peptidyl-tyrosine phosphorylation (GO:0018108) | 187 | 6 | 5.27 | 1.50E-02 |
| hair follicle development (GO:0001942) | 72 | 4 | 9.13 | 1.52E-02 |
| negative regulation of inflammatory response (GO:0050728) | 125 | 5 | 6.58 | 1.52E-02 |
| negative regulation of peptide secretion (GO:0002792) | 126 | 5 | 6.52 | 1.57E-02 |
| regulation of translation (GO:0006417) | 337 | 8 | 3.9 | 1.57E-02 |
| negative regulation of cell adhesion (GO:0007162) | 260 | 7 | 4.43 | 1.58E-02 |
| muscle adaptation (GO:0043500) | 32 | 3 | 15.41 | 1.59E-02 |
| positive regulation of transcription from RNA polymerase II promoter in response to stress (GO:0036003) | 32 | 3 | 15.41 | 1.59E-02 |
| extrinsic apoptotic signaling pathway via death domain receptors (GO:0008625) | 32 | 3 | 15.41 | 1.59E-02 |
| negative regulation of insulin receptor signaling pathway (GO:0046627) | 32 | 3 | 15.41 | 1.59E-02 |
| negative regulation of cellular response to transforming growth factor beta stimulus (GO:1903845) | 73 | 4 | 9.01 | 1.59E-02 |
| negative regulation of defense response (GO:0031348) | 190 | 6 | 5.19 | 1.60E-02 |
| peptidyl-tyrosine modification (GO:0018212) | 190 | 6 | 5.19 | 1.61E-02 |
| regulation of RNA stability (GO:0043487) | 127 | 5 | 6.47 | 1.62E-02 |
| skin epidermis development (GO:0098773) | 74 | 4 | 8.89 | 1.64E-02 |

| | | | | |
|---|---|---|---|---|
| regulation of transcription from RNA polymerase II promoter in response to stress (GO:0043618) | 74 | 4 | 8.89 | 1.64E-02 |
| positive regulation of growth (GO:0045927) | 263 | 7 | 4.38 | 1.65E-02 |
| regulation of hormone secretion (GO:0046883) | 264 | 7 | 4.36 | 1.68E-02 |
| CD4-positive, alpha-beta T cell differentiation (GO:0043367) | 33 | 3 | 14.94 | 1.69E-02 |
| regulation of tyrosine phosphorylation of STAT protein (GO:0042509) | 75 | 4 | 8.77 | 1.71E-02 |
| hair cycle process (GO:0022405) | 75 | 4 | 8.77 | 1.71E-02 |
| molting cycle process (GO:0022404) | 75 | 4 | 8.77 | 1.71E-02 |
| regulation of T cell migration (GO:2000404) | 34 | 3 | 14.51 | 1.83E-02 |
| embryonic digestive tract development (GO:0048566) | 34 | 3 | 14.51 | 1.83E-02 |
| positive regulation of small molecule metabolic process (GO:0062013) | 133 | 5 | 6.18 | 1.93E-02 |
| negative regulation of cellular response to insulin stimulus (GO:1900077) | 35 | 3 | 14.09 | 1.96E-02 |
| positive regulation of tissue remodeling (GO:0034105) | 35 | 3 | 14.09 | 1.97E-02 |
| negative regulation of animal organ morphogenesis (GO:0110111) | 35 | 3 | 14.09 | 1.97E-02 |
| NIK/NF-kappaB signaling (GO:0038061) | 35 | 3 | 14.09 | 1.97E-02 |
| homeostasis of number of cells (GO:0048872) | 200 | 6 | 4.93 | 1.98E-02 |
| Rho protein signal transduction (GO:0007266) | 79 | 4 | 8.32 | 2.00E-02 |
| positive regulation of JAK-STAT cascade (GO:0046427) | 79 | 4 | 8.32 | 2.00E-02 |
| cell-cell junction organization (GO:0045216) | 135 | 5 | 6.09 | 2.01E-02 |
| cellular response to catecholamine stimulus (GO:0071870) | 36 | 3 | 13.7 | 2.07E-02 |
| cellular response to monoamine stimulus (GO:0071868) | 36 | 3 | 13.7 | 2.07E-02 |
| mRNA stabilization (GO:0048255) | 36 | 3 | 13.7 | 2.07E-02 |
| positive regulation of bone mineralization (GO:0030501) | 36 | 3 | 13.7 | 2.07E-02 |
| cellular response to topologically incorrect protein (GO:0035967) | 136 | 5 | 6.04 | 2.07E-02 |
| regulation of epithelial cell differentiation (GO:0030856) | 136 | 5 | 6.04 | 2.07E-02 |
| negative regulation of hemopoiesis (GO:1903707) | 136 | 5 | 6.04 | 2.07E-02 |
| regulation of cell-substrate adhesion (GO:0010810) | 202 | 6 | 4.88 | 2.07E-02 |
| import into cell (GO:0098657) | 723 | 12 | 2.73 | 2.07E-02 |
| response to estradiol (GO:0032355) | 137 | 5 | 6 | 2.12E-02 |
| defense response to Gram-negative bacterium (GO:0050829) | 81 | 4 | 8.12 | 2.16E-02 |
| positive regulation of STAT cascade (GO:1904894) | 81 | 4 | 8.12 | 2.16E-02 |

| | | | | |
|---|---|---|---|---|
| negative regulation of protein modification by small protein conjugation or removal (GO:1903321) | 81 | 4 | 8.12 | 2.16E-02 |
| muscle tissue morphogenesis (GO:0060415) | 81 | 4 | 8.12 | 2.17E-02 |
| membrane protein proteolysis (GO:0033619) | 37 | 3 | 13.33 | 2.21E-02 |
| CD4-positive, alpha-beta T cell activation (GO:0035710) | 37 | 3 | 13.33 | 2.21E-02 |
| regulation of mRNA catabolic process (GO:0061013) | 140 | 5 | 5.87 | 2.31E-02 |
| cellular response to acid chemical (GO:0071229) | 207 | 6 | 4.76 | 2.31E-02 |
| cellular response to alcohol (GO:0097306) | 83 | 4 | 7.92 | 2.32E-02 |
| positive regulation of epidermis development (GO:0045684) | 38 | 3 | 12.98 | 2.34E-02 |
| negative regulation of interleukin-6 production (GO:0032715) | 38 | 3 | 12.98 | 2.34E-02 |
| B cell proliferation (GO:0042100) | 38 | 3 | 12.98 | 2.34E-02 |
| regulation of glycoprotein biosynthetic process (GO:0010559) | 38 | 3 | 12.98 | 2.34E-02 |
| negative regulation of lipid metabolic process (GO:0045833) | 84 | 4 | 7.83 | 2.41E-02 |
| regulation of plasma membrane bounded cell projection organization (GO:0120035) | 640 | 11 | 2.83 | 2.42E-02 |
| cell junction organization (GO:0034330) | 210 | 6 | 4.7 | 2.44E-02 |
| cellular response to cadmium ion (GO:0071276) | 39 | 3 | 12.65 | 2.49E-02 |
| positive regulation of nitric oxide metabolic process (GO:1904407) | 39 | 3 | 12.65 | 2.49E-02 |
| negative regulation of secretion (GO:0051048) | 211 | 6 | 4.67 | 2.49E-02 |
| positive regulation of nitric oxide biosynthetic process (GO:0045429) | 39 | 3 | 12.65 | 2.50E-02 |
| regulation of fibroblast proliferation (GO:0048145) | 85 | 4 | 7.74 | 2.50E-02 |
| ear development (GO:0043583) | 212 | 6 | 4.65 | 2.54E-02 |
| regulation of muscle adaptation (GO:0043502) | 86 | 4 | 7.65 | 2.59E-02 |
| non-canonical Wnt signaling pathway (GO:0035567) | 86 | 4 | 7.65 | 2.59E-02 |
| roof of mouth development (GO:0060021) | 86 | 4 | 7.65 | 2.60E-02 |
| regulation of neural precursor cell proliferation (GO:2000177) | 86 | 4 | 7.65 | 2.60E-02 |
| response to ionizing radiation (GO:0010212) | 145 | 5 | 5.67 | 2.61E-02 |
| regulation of G-protein coupled receptor protein signaling pathway (GO:0008277) | 145 | 5 | 5.67 | 2.61E-02 |
| regulation of cell projection organization (GO:0031344) | 648 | 11 | 2.79 | 2.63E-02 |
| response to catecholamine (GO:0071869) | 40 | 3 | 12.33 | 2.64E-02 |
| response to monoamine (GO:0071867) | 40 | 3 | 12.33 | 2.64E-02 |
| positive regulation of protein acetylation (GO:1901985) | 40 | 3 | 12.33 | 2.64E-02 |

| | | | | |
|---|---|---|---|---|
| regulation of peptidyl-threonine phosphorylation (GO:0010799) | 40 | 3 | 12.33 | 2.64E-02 |
| positive regulation of glial cell differentiation (GO:0045687) | 40 | 3 | 12.33 | 2.65E-02 |
| positive regulation of muscle cell differentiation (GO:0051149) | 87 | 4 | 7.56 | 2.68E-02 |
| response to glucocorticoid (GO:0051384) | 147 | 5 | 5.59 | 2.73E-02 |
| embryonic morphogenesis (GO:0048598) | 556 | 10 | 2.96 | 2.73E-02 |
| endocytosis (GO:0006897) | 653 | 11 | 2.77 | 2.74E-02 |
| muscle organ morphogenesis (GO:0048644) | 88 | 4 | 7.47 | 2.76E-02 |
| acute-phase response (GO:0006953) | 41 | 3 | 12.03 | 2.78E-02 |
| regulation of cell adhesion mediated by integrin (GO:0033628) | 41 | 3 | 12.03 | 2.78E-02 |
| response to testosterone (GO:0033574) | 41 | 3 | 12.03 | 2.78E-02 |
| RNA stabilization (GO:0043489) | 41 | 3 | 12.03 | 2.78E-02 |
| osteoclast differentiation (GO:0030316) | 41 | 3 | 12.03 | 2.78E-02 |
| positive regulation of protein catabolic process (GO:0045732) | 218 | 6 | 4.52 | 2.83E-02 |
| negative regulation of nervous system development (GO:0051961) | 295 | 7 | 3.9 | 2.83E-02 |
| regulation of autophagy (GO:0010506) | 295 | 7 | 3.9 | 2.83E-02 |
| regulation of lipid metabolic process (GO:0019216) | 379 | 8 | 3.47 | 2.86E-02 |
| regulation of protein modification by small protein conjugation or removal (GO:1903320) | 219 | 6 | 4.5 | 2.88E-02 |
| camera-type eye development (GO:0043010) | 296 | 7 | 3.89 | 2.88E-02 |
| negative regulation of osteoblast differentiation (GO:0045668) | 42 | 3 | 11.74 | 2.94E-02 |
| bone remodeling (GO:0046849) | 42 | 3 | 11.74 | 2.94E-02 |
| cellular monovalent inorganic cation homeostasis (GO:0030004) | 91 | 4 | 7.23 | 3.07E-02 |
| negative regulation of cellular response to oxidative stress (GO:1900408) | 43 | 3 | 11.47 | 3.09E-02 |
| negative regulation of I-kappaB kinase/NF-kappaB signaling (GO:0043124) | 43 | 3 | 11.47 | 3.09E-02 |
| positive regulation of phospholipase C activity (GO:0010863) | 43 | 3 | 11.47 | 3.09E-02 |
| regulation of muscle cell differentiation (GO:0051147) | 152 | 5 | 5.41 | 3.09E-02 |
| body morphogenesis (GO:0010171) | 43 | 3 | 11.47 | 3.10E-02 |
| regulation of glycoprotein metabolic process (GO:1903018) | 43 | 3 | 11.47 | 3.10E-02 |
| negative regulation of mRNA catabolic process (GO:1902373) | 43 | 3 | 11.47 | 3.10E-02 |
| positive regulation of calcium-mediated signaling (GO:0050850) | 43 | 3 | 11.47 | 3.10E-02 |
| modification by symbiont of host morphology or physiology (GO:0044003) | 43 | 3 | 11.47 | 3.10E-02 |

| | | | | |
|---|---|---|---|---|
| negative regulation of oxidative stress-induced cell death (GO:1903202) | 43 | 3 | 11.47 | 3.11E-02 |
| ventricular septum morphogenesis (GO:0060412) | 43 | 3 | 11.47 | 3.11E-02 |
| glial cell differentiation (GO:0010001) | 153 | 5 | 5.37 | 3.13E-02 |
| hair cycle (GO:0042633) | 92 | 4 | 7.15 | 3.14E-02 |
| molting cycle (GO:0042303) | 92 | 4 | 7.15 | 3.14E-02 |
| regulation of cell shape (GO:0008360) | 154 | 5 | 5.34 | 3.21E-02 |
| cellular response to amino acid starvation (GO:0034198) | 44 | 3 | 11.21 | 3.27E-02 |
| regulation of nitric-oxide synthase activity (GO:0050999) | 44 | 3 | 11.21 | 3.27E-02 |
| positive regulation of biomineral tissue development (GO:0070169) | 44 | 3 | 11.21 | 3.27E-02 |
| viral entry into host cell (GO:0046718) | 94 | 4 | 7 | 3.36E-02 |
| negative regulation of protein phosphorylation (GO:0001933) | 392 | 8 | 3.35 | 3.42E-02 |
| response to dexamethasone (GO:0071548) | 45 | 3 | 10.96 | 3.45E-02 |
| regulation of cardiocyte differentiation (GO:1905207) | 45 | 3 | 10.96 | 3.45E-02 |
| regulation of phospholipase C activity (GO:1900274) | 45 | 3 | 10.96 | 3.45E-02 |
| regulation of viral transcription (GO:0046782) | 45 | 3 | 10.96 | 3.45E-02 |
| regulation of macroautophagy (GO:0016241) | 157 | 5 | 5.24 | 3.45E-02 |
| negative regulation of fat cell differentiation (GO:0045599) | 45 | 3 | 10.96 | 3.46E-02 |
| developmental maturation (GO:0021700) | 229 | 6 | 4.31 | 3.47E-02 |
| multicellular organismal homeostasis (GO:0048871) | 308 | 7 | 3.74 | 3.47E-02 |
| response to calcium ion (GO:0051592) | 158 | 5 | 5.2 | 3.53E-02 |
| negative regulation of response to oxidative stress (GO:1902883) | 46 | 3 | 10.72 | 3.61E-02 |
| response to amino acid starvation (GO:1990928) | 46 | 3 | 10.72 | 3.62E-02 |
| release of sequestered calcium ion into cytosol (GO:0051209) | 46 | 3 | 10.72 | 3.62E-02 |
| positive regulation of T cell mediated immunity (GO:0002711) | 46 | 3 | 10.72 | 3.62E-02 |
| heart valve morphogenesis (GO:0003179) | 46 | 3 | 10.72 | 3.62E-02 |
| neuron development (GO:0048666) | 784 | 12 | 2.52 | 3.62E-02 |
| embryonic skeletal system morphogenesis (GO:0048704) | 97 | 4 | 6.78 | 3.68E-02 |
| regulation of circadian rhythm (GO:0042752) | 97 | 4 | 6.78 | 3.68E-02 |
| negative regulation of small molecule metabolic process (GO:0062014) | 97 | 4 | 6.78 | 3.68E-02 |
| fatty acid derivative metabolic process (GO:1901568) | 160 | 5 | 5.14 | 3.68E-02 |
| positive regulation of cell cycle (GO:0045787) | 398 | 8 | 3.3 | 3.68E-02 |

| | | | | |
|---|---|---|---|---|
| negative regulation of leukocyte differentiation (GO:1902106) | 98 | 4 | 6.71 | 3.80E-02 |
| face development (GO:0060324) | 47 | 3 | 10.49 | 3.81E-02 |
| negative regulation of cold-induced thermogenesis (GO:0120163) | 47 | 3 | 10.49 | 3.81E-02 |
| regulation of striated muscle cell differentiation (GO:0051153) | 99 | 4 | 6.64 | 3.93E-02 |
| regulation of calcium ion transport into cytosol (GO:0010522) | 99 | 4 | 6.64 | 3.93E-02 |
| response to corticosteroid (GO:0031960) | 163 | 5 | 5.04 | 3.95E-02 |
| trabecula morphogenesis (GO:0061383) | 48 | 3 | 10.27 | 3.98E-02 |
| positive regulation of reactive oxygen species biosynthetic process (GO:1903428) | 48 | 3 | 10.27 | 3.98E-02 |
| negative regulation of sequestering of calcium ion (GO:0051283) | 48 | 3 | 10.27 | 3.99E-02 |
| calcium ion transport (GO:0006816) | 239 | 6 | 4.13 | 4.15E-02 |
| lymphocyte activation involved in immune response (GO:0002285) | 101 | 4 | 6.51 | 4.16E-02 |
| response to nicotine (GO:0035094) | 49 | 3 | 10.06 | 4.20E-02 |
| skeletal system morphogenesis (GO:0048705) | 240 | 6 | 4.11 | 4.22E-02 |
| protein complex oligomerization (GO:0051259) | 501 | 9 | 2.95 | 4.22E-02 |
| positive regulation of transmembrane receptor protein serine/threonine kinase signaling pathway (GO:0090100) | 102 | 4 | 6.45 | 4.28E-02 |
| negative regulation of protein binding (GO:0032091) | 102 | 4 | 6.45 | 4.28E-02 |
| entry into host cell (GO:0030260) | 102 | 4 | 6.45 | 4.28E-02 |
| entry into other organism involved in symbiotic interaction (GO:0051828) | 102 | 4 | 6.45 | 4.29E-02 |
| entry into cell of other organism involved in symbiotic interaction (GO:0051806) | 102 | 4 | 6.45 | 4.29E-02 |
| entry into host (GO:0044409) | 102 | 4 | 6.45 | 4.29E-02 |
| negative regulation of RNA catabolic process (GO:1902369) | 50 | 3 | 9.86 | 4.40E-02 |
| regulation of synapse assembly (GO:0051963) | 103 | 4 | 6.38 | 4.42E-02 |
| negative regulation of protein transport (GO:0051224) | 170 | 5 | 4.84 | 4.60E-02 |
| regulation of plasma membrane bounded cell projection assembly (GO:0120032) | 170 | 5 | 4.84 | 4.60E-02 |
| regulation of histone acetylation (GO:0035065) | 51 | 3 | 9.67 | 4.61E-02 |
| negative regulation of endocytosis (GO:0045806) | 51 | 3 | 9.67 | 4.61E-02 |
| epidermal growth factor receptor signaling pathway (GO:0007173) | 51 | 3 | 9.67 | 4.62E-02 |
| positive regulation of autophagy (GO:0010508) | 105 | 4 | 6.26 | 4.68E-02 |
| regulation of transforming growth factor beta receptor signaling pathway (GO:0017015) | 105 | 4 | 6.26 | 4.69E-02 |

| | | | | |
|---|---|---|---|---|
| regulation of cell projection assembly (GO:0060491) | 172 | 5 | 4.78 | 4.80E-02 |
| negative regulation of reactive oxygen species metabolic process (GO:2000378) | 52 | 3 | 9.48 | 4.83E-02 |
| regulation of nitric oxide biosynthetic process (GO:0045428) | 52 | 3 | 9.48 | 4.84E-02 |
| leukocyte activation involved in immune response (GO:0002366) | 611 | 10 | 2.69 | 4.84E-02 |
| positive regulation of cytokine production involved in inflammatory response (GO:1900017) | 15 | 2 | 21.92 | 4.96E-02 |
| positive regulation of type 2 immune response (GO:0002830) | 15 | 2 | 21.92 | 4.96E-02 |
| negative regulation of leukocyte chemotaxis (GO:0002689) | 15 | 2 | 21.92 | 4.96E-02 |
| negative regulation of myeloid cell apoptotic process (GO:0033033) | 15 | 2 | 21.92 | 4.97E-02 |
| negative regulation of cell migration involved in sprouting angiogenesis (GO:0090051) | 15 | 2 | 21.92 | 4.97E-02 |
| interleukin-6-mediated signaling pathway (GO:0070102) | 15 | 2 | 21.92 | 4.97E-02 |
| regulation of cellular response to transforming growth factor beta stimulus (GO:1903844) | 107 | 4 | 6.15 | 4.97E-02 |
| regulation of podosome assembly (GO:0071801) | 15 | 2 | 21.92 | 4.98E-02 |
| positive regulation of interleukin-13 production (GO:0032736) | 15 | 2 | 21.92 | 4.98E-02 |
| regulation of DNA replication (GO:0006275) | 107 | 4 | 6.15 | 4.98E-02 |
| negative regulation of establishment of protein localization (GO:1904950) | 174 | 5 | 4.72 | 4.99E-02 |
