# Supplementary material for: Gene expression profiling distinguishes prefibrotic from overtly fibrotic myeloproliferative neoplasms and identifies disease subsets with distinct inflammatory signatures
Source: PLoS One. 2019 May 9;14(5):e0216810. doi: 10.1371/journal.pone.0216810 (PMC6534080; doi:10.1371/journal.pone.0216810)
Supplement: S3 Table — (PDF) [file pone.0216810.s003.pdf]

Supplementary Table 3. Differentially expressed genes in *ASXL1* mutant MPN

Gene	Accession #	Class Name	Fold Change	FDR
TNF	NM_000594.2	Endogenous	1.997644454	0.00124755
KEAP1	NM_012289.3	Endogenous	1.448390906	0.001571147
TLR3	NM_003265.2	Endogenous	2.242367339	0.002029959
PTK2	NM_005607.3	Endogenous	1.760867445	0.002029959
PRKCA	NM_002737.2	Endogenous	1.873774557	0.002454124
DAXX	NM_001350.3	Endogenous	1.277181491	0.002454124
MAPK8	NM_002750.2	Endogenous	1.337665107	0.002654181
SHC1	NM_001130040.1	Endogenous	1.478693146	0.003271173
HSPB2	NM_001541.3	Endogenous	2.345643786	0.003271173
ROCK2	NM_004850.3	Endogenous	1.423928709	0.004789658
ELK1	NM_005229.3	Endogenous	1.518128204	0.005529966
PTGIR	NM_000960.3	Endogenous	2.044793633	0.005529966
TCF4	NM_003199.1	Endogenous	1.694764505	0.005529966
PDGFA	NM_002607.5	Endogenous	2.197411324	0.005529966
RIPK2	NM_003821.5	Endogenous	1.423404996	0.005529966
CCL8	NM_005623.2	Endogenous	2.31717802	0.005529966
CCL2	NM_002982.3	Endogenous	2.605058756	0.005529966
MAX	NM_002382.3	Endogenous	1.435244408	0.005529966
MEF2A	NM_005587.2	Endogenous	1.501140588	0.005536145
PTGER4	NM_000958.2	Endogenous	1.472121097	0.006976064
FLT1	NM_002019.4	Endogenous	1.732461978	0.006976064
CXCL3	NM_002090.2	Endogenous	2.312267357	0.008327024
RAC1	NM_198829.1	Endogenous	1.428404242	0.008327024
PTGER3	NM_000957.2	Endogenous	1.844764709	0.008327024
CSF1	NM_000757.4	Endogenous	1.728429004	0.009354092
CCR4	NM_005508.4	Endogenous	2.012846202	0.009354092
MAP2K4	NM_003010.2	Endogenous	1.31157648	0.009354092
CCL5	NM_002985.2	Endogenous	2.044718359	0.009354092
SMAD7	NM_005904.2	Endogenous	1.849036526	0.009354092
CD40LG	NM_000074.2	Endogenous	1.813907489	0.011767982
TBXA2R	NM_001060.3	Endogenous	1.792519929	0.017377281
TGFB3	NM_003239.2	Endogenous	2.216383849	0.017509073
HSPB1	NM_001540.3	Endogenous	1.625956306	0.017509073
ATF2	NM_001880.2	Endogenous	1.256094369	0.017765451
CCL13	NM_005408.2	Endogenous	2.572002746	0.017768754
CD40	NM_001250.4	Endogenous	1.655502562	0.017786064
CXCL2	NM_002089.3	Endogenous	1.990999211	0.017786064
DDIT3	NM_004083.4	Endogenous	1.549409673	0.019608694
TGFB2	NM_003238.2	Endogenous	2.095550623	0.019608694
MAPKAPK5	NM_003668.2	Endogenous	1.209103204	0.021568997
MAPKAPK2	NM_004759.3	Endogenous	1.224314285	0.025975475
TNFAIP3	NM_006290.2	Endogenous	1.569252731	0.025975475
GNAQ	NM_002072.2	Endogenous	1.267898632	0.025975475
MEF2D	NM_005920.2	Endogenous	1.487715404	0.025975475
TGFB1	NM_000660.3	Endogenous	1.601329961	0.025975475

RELA	NM_021975.2	Endogenous	1.307282922	0.025975475
MAP3K5	NM_005923.3	Endogenous	1.317794659	0.029709462
MEF2C	NM_002397.3	Endogenous	1.648650663	0.031385207
TLR9	NM_017442.2	Endogenous	1.645327936	0.035520835
FXYD2	NM_021603.3	Endogenous	1.857700002	0.035911541
RIPK1	NM_003804.3	Endogenous	1.244280554	0.036424744
FASLG	NM_000639.1	Endogenous	1.896202388	0.038292543
JUN	NM_002228.3	Endogenous	2.083039643	0.04245127
TGFBR1	NM_004612.2	Endogenous	1.366863488	0.045607715
IL1R1	NM_000877.2	Endogenous	1.483423531	0.045607715
C4A	NM_007293.2	Endogenous	1.666603902	0.045607715
CD163	NM_004244.4	Endogenous	1.563838289	0.045607715
C1S	NM_001734.2	Endogenous	1.840361733	0.045607715
