# Supplementary material for: Gene expression profiling distinguishes prefibrotic from overtly fibrotic myeloproliferative neoplasms and identifies disease subsets with distinct inflammatory signatures
Source: PLoS One. 2019 May 9;14(5):e0216810. doi: 10.1371/journal.pone.0216810 (PMC6534080; doi:10.1371/journal.pone.0216810)
Supplement: S4 Table — (PDF) [file pone.0216810.s004.pdf]

Supplementary Table 4. Gene ontology terms enriched in *ASXL1* mutant MPN

Analysis Type:	PANTHER Overrepresentation Test (Released 20171205)
Annotation Version and Release Date:	GO Ontology database Released 2018-08-09
Analyzed List:	*ASXL1* mutant MPN (Homo sapiens)
Reference List:	Homo sapiens (all genes in database)
Test Type:	FISHER

**GO biological process complete**	**REFLIST**	***ASXL1* mutant**	**Fold enrichment**	**FDR**
inflammatory response (GO:0006954)	466	26	18.94	5.69E-23
protein phosphorylation (GO:0006468)	927	31	11.35	3.95E-22
positive regulation of MAPK cascade (GO:0043410)	552	26	15.99	1.50E-21
positive regulation of protein phosphorylation (GO:0001934)	990	29	9.94	2.57E-19
signal transduction by protein phosphorylation (GO:0023014)	356	21	20.02	5.38E-19
regulation of MAPK cascade (GO:0043408)	753	26	11.72	9.15E-19
response to molecule of bacterial origin (GO:0002237)	323	20	21.01	1.72E-18
response to biotic stimulus (GO:0009607)	950	27	9.65	1.18E-17
response to lipopolysaccharide (GO:0032496)	307	19	21	1.57E-17
regulation of protein kinase activity (GO:0045859)	765	25	11.09	1.68E-17
positive regulation of protein kinase activity (GO:0045860)	518	22	14.41	2.27E-17
MAPK cascade (GO:0000165)	328	19	19.66	4.66E-17
response to other organism (GO:0051707)	911	26	9.69	5.79E-17
response to external biotic stimulus (GO:0043207)	913	26	9.66	6.00E-17
positive regulation of kinase activity (GO:0033674)	559	22	13.36	9.68E-17
response to bacterium (GO:0009617)	643	23	12.14	9.75E-17
regulation of kinase activity (GO:0043549)	832	25	10.2	9.98E-17
regulation of transferase activity (GO:0051338)	942	26	9.37	1.18E-16
response to lipid (GO:0033993)	846	25	10.03	1.40E-16
positive regulation of MAP kinase activity (GO:0043406)	264	17	21.85	7.56E-16
positive regulation of cell proliferation (GO:0008284)	922	25	9.2	9.53E-16

positive regulation of transferase activity (GO:0051347)	631	22	11.83	1.00E-15
positive regulation of cell death (GO:0010942)	667	22	11.19	3.02E-15
regulation of cytokine production (GO:0001817)	634	21	11.24	1.67E-14
regulation of MAP kinase activity (GO:0043405)	341	17	16.92	3.61E-14
positive regulation of protein serine/threonine kinase activity (GO:0071902)	341	17	16.92	3.65E-14
regulation of protein serine/threonine kinase activity (GO:0071900)	506	19	12.74	6.67E-14
regulation of cell migration (GO:0030334)	799	22	9.34	9.74E-14
response to growth factor (GO:0070848)	521	19	12.38	1.08E-13
positive regulation of apoptotic process (GO:0043065)	609	20	11.15	1.08E-13
positive regulation of programmed cell death (GO:0043068)	615	20	11.04	1.26E-13
regulation of locomotion (GO:0040012)	930	23	8.39	1.56E-13
cellular response to cytokine stimulus (GO:0071345)	931	23	8.38	1.58E-13
cellular response to oxygen-containing compound (GO:1901701)	960	23	8.13	2.92E-13
regulation of cell motility (GO:2000145)	854	22	8.74	3.29E-13
cellular response to biotic stimulus (GO:0071216)	206	14	23.07	3.31E-13
positive regulation of locomotion (GO:0040017)	519	18	11.77	1.37E-12
cellular response to lipopolysaccharide (GO:0071222)	179	13	24.65	1.50E-12
regulation of response to external stimulus (GO:0032101)	816	21	8.73	1.54E-12
regulation of cellular component movement (GO:0051270)	935	22	7.99	1.80E-12
cellular response to molecule of bacterial origin (GO:0071219)	186	13	23.72	2.30E-12
positive regulation of cell migration (GO:0030335)	468	17	12.33	3.88E-12
positive regulation of cytokine production (GO:0001819)	403	16	13.47	6.55E-12
positive regulation of cell motility (GO:2000147)	486	17	11.87	6.80E-12
positive regulation of cellular component movement (GO:0051272)	500	17	11.54	1.02E-11
cellular response to lipid (GO:0071396)	522	17	11.05	1.94E-11
cytokine-mediated signaling pathway (GO:0019221)	619	18	9.87	2.12E-11
activation of protein kinase activity (GO:0032147)	313	14	15.18	5.96E-11
apoptotic process (GO:0006915)	889	20	7.64	7.43E-11
cellular response to growth factor stimulus (GO:0071363)	490	16	11.08	1.04E-10
regulation of DNA-binding transcription factor activity (GO:0051090)	408	15	12.48	1.15E-10
negative regulation of apoptotic process (GO:0043066)	922	20	7.36	1.36E-10

activation of MAPK activity (GO:0000187)	152	11	24.56	1.62E-10
negative regulation of programmed cell death (GO:0043069)	936	20	7.25	1.75E-10
stress-activated MAPK cascade (GO:0051403)	108	10	31.42	1.80E-10
regulation of angiogenesis (GO:0045765)	282	13	15.65	2.82E-10
positive regulation of response to external stimulus (GO:0032103)	284	13	15.54	3.03E-10
regulation of neuron death (GO:1901214)	292	13	15.11	4.19E-10
positive regulation of immune response (GO:0050778)	763	18	8.01	5.64E-10
regulation of production of molecular mediator of immune response (GO:0002700)	125	10	27.15	6.61E-10
regulation of signaling receptor activity (GO:0010469)	562	16	9.66	6.88E-10
positive regulation of neuron death (GO:1901216)	84	9	36.36	6.95E-10
positive regulation of ERK1 and ERK2 cascade (GO:0070374)	239	12	17.04	7.59E-10
regulation of vasculature development (GO:1901342)	310	13	14.23	8.03E-10
JNK cascade (GO:0007254)	86	9	35.52	8.09E-10
circulatory system development (GO:0072359)	839	18	7.28	2.39E-09
stress-activated protein kinase signaling cascade (GO:0031098)	145	10	23.41	2.42E-09
regulation of immune effector process (GO:0002697)	436	14	10.9	3.40E-09
positive regulation of stress-activated MAPK cascade (GO:0032874)	161	10	21.08	6.26E-09
positive regulation of stress-activated protein kinase signaling cascade (GO:0070304)	162	10	20.95	6.59E-09
response to wounding (GO:0009611)	560	15	9.09	7.13E-09
enzyme linked receptor protein signaling pathway (GO:0007167)	686	16	7.92	1.09E-08
positive regulation of JUN kinase activity (GO:0043507)	76	8	35.72	1.11E-08
regulation of ERK1 and ERK2 cascade (GO:0070372)	311	12	13.1	1.26E-08
myeloid leukocyte migration (GO:0097529)	123	9	24.83	1.46E-08
regulation of epithelial cell proliferation (GO:0050678)	320	12	12.73	1.71E-08
regulation of smooth muscle cell proliferation (GO:0048660)	131	9	23.32	2.42E-08
positive regulation of DNA-binding transcription factor activity (GO:0051091)	254	11	14.7	2.42E-08
chemotaxis (GO:0006935)	518	14	9.17	2.80E-08
immune response-regulating signaling pathway (GO:0002764)	520	14	9.14	2.91E-08
taxis (GO:0042330)	520	14	9.14	2.93E-08
regulation of JUN kinase activity (GO:0043506)	90	8	30.17	3.61E-08
positive regulation of JNK cascade (GO:0046330)	138	9	22.13	3.61E-08

leukocyte chemotaxis (GO:0030595)	139	9	21.97	3.81E-08
cell chemotaxis (GO:0060326)	198	10	17.14	3.83E-08
positive regulation of hydrolase activity (GO:0051345)	754	16	7.2	3.83E-08
cellular response to tumor necrosis factor (GO:0071356)	199	10	17.05	3.97E-08
positive regulation of NF-kappaB transcription factor activity (GO:0051092)	145	9	21.07	5.30E-08
apoptotic signaling pathway (GO:0097190)	277	11	13.48	5.40E-08
response to organic cyclic compound (GO:0014070)	901	17	6.4	5.56E-08
leukocyte migration (GO:0050900)	361	12	11.28	5.84E-08
regulation of epithelial cell migration (GO:0010632)	210	10	16.16	6.32E-08
response to interleukin-1 (GO:0070555)	149	9	20.5	6.46E-08
cell migration (GO:0016477)	913	17	6.32	6.65E-08
regulation of leukocyte proliferation (GO:0070663)	218	10	15.57	8.81E-08
positive regulation of defense response (GO:0031349)	378	12	10.77	9.44E-08
positive regulation of interleukin-12 production (GO:0032735)	32	6	63.64	9.60E-08
response to transforming growth factor beta (GO:0071559)	157	9	19.46	9.77E-08
regulation of stress-activated MAPK cascade (GO:0032872)	221	10	15.36	9.81E-08
positive regulation of cell differentiation (GO:0045597)	939	17	6.14	9.82E-08
response to tumor necrosis factor (GO:0034612)	222	10	15.29	1.00E-07
regulation of stress-activated protein kinase signaling cascade (GO:0070302)	222	10	15.29	1.01E-07
regulation of I-kappaB kinase/NF-kappaB signaling (GO:0043122)	222	10	15.29	1.01E-07
blood vessel development (GO:0001568)	478	13	9.23	1.06E-07
regulation of cytokine production involved in immune response (GO:0002718)	68	7	34.94	1.59E-07
I-kappaB kinase/NF-kappaB signaling (GO:0007249)	68	7	34.94	1.60E-07
vasculature development (GO:0001944)	501	13	8.81	1.78E-07
heart development (GO:0007507)	505	13	8.74	1.95E-07
regulation of leukocyte migration (GO:0002685)	172	9	17.76	1.96E-07
cellular calcium ion homeostasis (GO:0006874)	409	12	9.96	2.06E-07
cardiovascular system development (GO:0072358)	512	13	8.62	2.26E-07
calcium ion homeostasis (GO:0055074)	422	12	9.65	2.86E-07
regulation of JNK cascade (GO:0046328)	181	9	16.88	2.92E-07
regulation of ossification (GO:0030278)	181	9	16.88	2.93E-07

regulation of defense response (GO:0031347)	755	15	6.74	2.99E-07
chemokine-mediated signaling pathway (GO:0070098)	76	7	31.26	3.08E-07
negative regulation of epithelial cell proliferation (GO:0050680)	124	8	21.9	3.18E-07
response to hormone (GO:0009725)	889	16	6.11	3.22E-07
activation of JUN kinase activity (GO:0007257)	41	6	49.67	3.23E-07
cellular response to interleukin-1 (GO:0071347)	126	8	21.55	3.54E-07
cellular response to mechanical stimulus (GO:0071260)	80	7	29.7	4.18E-07
cellular divalent inorganic cation homeostasis (GO:0072503)	441	12	9.24	4.33E-07
neutrophil chemotaxis (GO:0030593)	81	7	29.33	4.47E-07
regulation of leukocyte differentiation (GO:1902105)	265	10	12.81	4.50E-07
response to chemokine (GO:1990868)	82	7	28.97	4.79E-07
cellular response to chemokine (GO:1990869)	82	7	28.97	4.81E-07
animal organ morphogenesis (GO:0009887)	924	16	5.88	5.27E-07
regulation of neuron apoptotic process (GO:0043523)	199	9	15.35	5.93E-07
negative regulation of cell proliferation (GO:0008285)	676	14	7.03	5.95E-07
divalent inorganic cation homeostasis (GO:0072507)	462	12	8.82	6.88E-07
tube development (GO:0035295)	813	15	6.26	7.21E-07
granulocyte chemotaxis (GO:0071621)	88	7	27	7.34E-07
wound healing (GO:0042060)	470	12	8.67	8.19E-07
neutrophil migration (GO:1990266)	90	7	26.4	8.42E-07
regulation of vascular smooth muscle cell proliferation (GO:1904705)	50	6	40.73	8.69E-07
positive regulation of neuron apoptotic process (GO:0043525)	50	6	40.73	8.73E-07
regulation of adaptive immune response based on somatic recombination of immune receptors built from immunoglobulin superfamily domains (GO:0002822)	144	8	18.85	8.73E-07
extrinsic apoptotic signaling pathway (GO:0097191)	92	7	25.82	9.53E-07
response to mechanical stimulus (GO:0009612)	213	9	14.34	9.92E-07
regulation of interleukin-12 production (GO:0032655)	52	6	39.16	1.06E-06
cellular response to transforming growth factor beta stimulus (GO:0071560)	149	8	18.22	1.11E-06
regulation of endothelial cell migration (GO:0010594)	150	8	18.1	1.16E-06
negative regulation of cytokine production involved in immune response (GO:0002719)	24	5	70.71	1.22E-06
transforming growth factor beta receptor signaling pathway (GO:0007179)	96	7	24.75	1.23E-06

regulation of hemopoiesis (GO:1903706)	391	11	9.55	1.28E-06
regulation of cell activation (GO:0050865)	607	13	7.27	1.36E-06
blood vessel morphogenesis (GO:0048514)	396	11	9.43	1.43E-06
positive regulation of cell adhesion (GO:0045785)	397	11	9.4	1.45E-06
granulocyte migration (GO:0097530)	99	7	24	1.46E-06
regulation of adaptive immune response (GO:0002819)	156	8	17.4	1.49E-06
negative regulation of angiogenesis (GO:0016525)	104	7	22.84	1.96E-06
regulation of inflammatory response (GO:0050727)	410	11	9.11	1.97E-06
positive regulation of cytokine biosynthetic process (GO:0042108)	59	6	34.51	1.99E-06
cellular response to external stimulus (GO:0071496)	318	10	10.67	2.06E-06
tube morphogenesis (GO:0035239)	634	13	6.96	2.13E-06
negative regulation of blood vessel morphogenesis (GO:2000181)	106	7	22.41	2.19E-06
programmed necrotic cell death (GO:0097300)	28	5	60.6	2.24E-06
cellular response to drug (GO:0035690)	327	10	10.38	2.60E-06
cellular metal ion homeostasis (GO:0006875)	530	12	7.68	2.61E-06
regulation of cellular response to stress (GO:0080135)	647	13	6.82	2.63E-06
pattern recognition receptor signaling pathway (GO:0002221)	110	7	21.6	2.72E-06
necrotic cell death (GO:0070265)	30	5	56.56	2.96E-06
regulation of cell adhesion (GO:0030155)	655	13	6.74	2.98E-06
negative regulation of developmental process (GO:0051093)	918	15	5.55	2.98E-06
regulation of protein secretion (GO:0050708)	430	11	8.68	2.99E-06
positive regulation of I-kappaB kinase/NF-kappaB signaling (GO:0043123)	175	8	15.51	3.23E-06
negative regulation of cytokine production (GO:0001818)	253	9	12.07	3.60E-06
activation of immune response (GO:0002253)	550	12	7.4	3.72E-06
vascular endothelial growth factor receptor signaling pathway (GO:0048010)	67	6	30.39	3.73E-06
positive regulation of leukocyte migration (GO:0002687)	116	7	20.48	3.73E-06
positive regulation of tumor necrosis factor production (GO:0032760)	67	6	30.39	3.74E-06
lipopolysaccharide-mediated signaling pathway (GO:0031663)	32	5	53.03	3.82E-06
negative regulation of vasculature development (GO:1901343)	117	7	20.31	3.88E-06
regulation of tumor necrosis factor production (GO:0032680)	118	7	20.13	4.09E-06

negative regulation of production of molecular mediator of immune response (GO:0002701)	33	5	51.42	4.35E-06
positive regulation of tumor necrosis factor superfamily cytokine production (GO:1903557)	70	6	29.09	4.68E-06
response to drug (GO:0042493)	956	15	5.33	4.80E-06
regulation of tumor necrosis factor superfamily cytokine production (GO:1903555)	122	7	19.47	5.01E-06
positive regulation of phosphatidylinositol 3-kinase signaling (GO:0014068)	71	6	28.68	5.02E-06
regulation of peptide secretion (GO:0002791)	458	11	8.15	5.26E-06
regulation of endothelial cell proliferation (GO:0001936)	124	7	19.16	5.50E-06
response to ketone (GO:1901654)	195	8	13.92	6.76E-06
cellular cation homeostasis (GO:0030003)	589	12	6.91	7.32E-06
protein kinase B signaling (GO:0043491)	38	5	44.66	7.92E-06
negative regulation of endothelial cell proliferation (GO:0001937)	38	5	44.66	7.94E-06
metal ion homeostasis (GO:0055065)	597	12	6.82	8.33E-06
cellular response to nitrogen compound (GO:1901699)	599	12	6.8	8.61E-06
transmembrane receptor protein tyrosine kinase signaling pathway (GO:0007169)	485	11	7.7	8.89E-06
cellular response to hormone stimulus (GO:0032870)	601	12	6.78	8.89E-06
cellular ion homeostasis (GO:0006873)	603	12	6.75	9.15E-06
regulation of cell-cell adhesion (GO:0022407)	382	10	8.88	9.40E-06
positive regulation of mononuclear cell proliferation (GO:0032946)	137	7	17.34	1.01E-05
positive regulation of epithelial cell migration (GO:0010634)	137	7	17.34	1.01E-05
ion homeostasis (GO:0050801)	737	13	5.99	1.02E-05
regulation of lymphocyte activation (GO:0051249)	497	11	7.51	1.11E-05
positive regulation of leukocyte proliferation (GO:0070665)	140	7	16.97	1.15E-05
regulation of mononuclear cell proliferation (GO:0032944)	211	8	12.87	1.15E-05
regulation of animal organ morphogenesis (GO:2000027)	212	8	12.81	1.18E-05
monocyte chemotaxis (GO:0002548)	42	5	40.4	1.19E-05
leukocyte proliferation (GO:0070661)	84	6	24.24	1.19E-05
positive regulation of leukocyte differentiation (GO:1902107)	143	7	16.61	1.30E-05
regulation of actomyosin structure organization (GO:0110020)	87	6	23.41	1.43E-05
regulation of blood vessel endothelial cell migration (GO:0043535)	87	6	23.41	1.43E-05

cellular response to abiotic stimulus (GO:0071214)	304	9	10.05	1.43E-05
cellular response to environmental stimulus (GO:0104004)	304	9	10.05	1.43E-05
regulation of secretion (GO:0051046)	763	13	5.78	1.44E-05
mononuclear cell migration (GO:0071674)	44	5	38.57	1.45E-05
angiogenesis (GO:0001525)	309	9	9.89	1.62E-05
regulation of endothelial cell apoptotic process (GO:2000351)	46	5	36.89	1.75E-05
positive regulation of endothelial cell apoptotic process (GO:2000353)	18	4	75.42	2.10E-05
positive regulation of interleukin-8 production (GO:0032757)	48	5	35.35	2.10E-05
regulation of cytokine-mediated signaling pathway (GO:0001959)	155	7	15.33	2.10E-05
peptidyl-serine phosphorylation (GO:0018105)	156	7	15.23	2.16E-05
cation homeostasis (GO:0055080)	660	12	6.17	2.16E-05
negative regulation of immune system process (GO:0002683)	423	10	8.02	2.17E-05
cardiac muscle tissue development (GO:0048738)	160	7	14.85	2.54E-05
regulation of cytokine biosynthetic process (GO:0042035)	98	6	20.78	2.63E-05
inorganic ion homeostasis (GO:0098771)	673	12	6.05	2.63E-05
heart morphogenesis (GO:0003007)	242	8	11.22	2.91E-05
positive regulation of myeloid leukocyte differentiation (GO:0002763)	52	5	32.63	2.97E-05
regulation of response to cytokine stimulus (GO:0060759)	165	7	14.4	3.06E-05
innate immune response-activating signal transduction (GO:0002758)	166	7	14.31	3.17E-05
cellular chemical homeostasis (GO:0055082)	691	12	5.89	3.39E-05
positive regulation of cell-cell adhesion (GO:0022409)	248	8	10.95	3.42E-05
eosinophil chemotaxis (GO:0048245)	21	4	64.65	3.45E-05
regulation of myoblast differentiation (GO:0045661)	54	5	31.42	3.46E-05
positive regulation of proteolysis (GO:0045862)	345	9	8.85	3.67E-05
regulation of phosphatidylinositol 3-kinase signaling (GO:0014066)	105	6	19.39	3.71E-05
regulation of leukocyte activation (GO:0002694)	572	11	6.53	3.82E-05
negative regulation of extrinsic apoptotic signaling pathway (GO:2001237)	106	6	19.21	3.88E-05
cellular homeostasis (GO:0019725)	841	13	5.25	3.89E-05
eosinophil migration (GO:0072677)	22	4	61.71	3.93E-05
necroptotic process (GO:0070266)	22	4	61.71	3.94E-05
regulation of secretion by cell (GO:1903530)	705	12	5.78	4.00E-05

regulation of establishment of protein localization (GO:0070201)	708	12	5.75	4.16E-05
innate immune response (GO:0045087)	708	12	5.75	4.17E-05
peptidyl-serine modification (GO:0018209)	175	7	13.58	4.20E-05
interspecies interaction between organisms (GO:0044419)	709	12	5.74	4.21E-05
positive regulation of lymphocyte activation (GO:0051251)	359	9	8.51	4.87E-05
positive regulation of hemopoiesis (GO:1903708)	181	7	13.13	5.17E-05
anatomical structure formation involved in morphogenesis (GO:0048646)	866	13	5.09	5.18E-05
positive regulation of pri-miRNA transcription by RNA polymerase II (GO:1902895)	24	4	56.56	5.20E-05
positive regulation of innate immune response (GO:0045089)	265	8	10.25	5.20E-05
activation of innate immune response (GO:0002218)	184	7	12.91	5.68E-05
regulation of myeloid leukocyte differentiation (GO:0002761)	115	6	17.71	5.83E-05
regulation of pathway-restricted SMAD protein phosphorylation (GO:0060393)	62	5	27.37	6.12E-05
immune response-activating signal transduction (GO:0002757)	485	10	7	6.49E-05
regulation of immunoglobulin production (GO:0002637)	63	5	26.94	6.55E-05
response to virus (GO:0009615)	275	8	9.87	6.66E-05
regulation of interleukin-6 production (GO:0032675)	119	6	17.11	6.97E-05
transmembrane receptor protein serine/threonine kinase signaling pathway (GO:0007178)	192	7	12.37	7.33E-05
immune system development (GO:0002520)	618	11	6.04	7.46E-05
cranial skeletal system development (GO:1904888)	65	5	26.11	7.48E-05
striated muscle tissue development (GO:0014706)	281	8	9.66	7.69E-05
positive regulation of interferon-gamma production (GO:0032729)	66	5	25.71	7.99E-05
regulation of interleukin-8 production (GO:0032677)	67	5	25.33	8.55E-05
regulation of leukocyte cell-cell adhesion (GO:1903037)	286	8	9.49	8.68E-05
regulation of chemotaxis (GO:0050920)	198	7	12	8.78E-05
regulation of apoptotic signaling pathway (GO:2001233)	390	9	7.83	8.98E-05
positive regulation of chemotaxis (GO:0050921)	126	6	16.16	9.34E-05
response to extracellular stimulus (GO:0009991)	509	10	6.67	9.56E-05
salivary gland morphogenesis (GO:0007435)	29	4	46.81	9.76E-05
muscle tissue development (GO:0060537)	294	8	9.24	1.04E-04
positive regulation of leukocyte activation (GO:0002696)	398	9	7.67	1.04E-04

positive regulation of cardiac muscle hypertrophy (GO:0010613)	30	4	45.25	1.09E-04
positive regulation of epithelial cell apoptotic process (GO:1904037)	30	4	45.25	1.09E-04
positive regulation of muscle hypertrophy (GO:0014742)	30	4	45.25	1.09E-04
regulation of epithelial cell apoptotic process (GO:1904035)	72	5	23.57	1.15E-04
cardiac ventricle morphogenesis (GO:0003208)	72	5	23.57	1.15E-04
regulation of lymphocyte proliferation (GO:0050670)	210	7	11.31	1.23E-04
positive regulation of leukocyte cell-cell adhesion (GO:1903039)	210	7	11.31	1.24E-04
positive regulation of cell activation (GO:0050867)	408	9	7.49	1.24E-04
negative regulation of cell cycle (GO:0045786)	532	10	6.38	1.36E-04
positive regulation of lymphocyte proliferation (GO:0050671)	136	6	14.97	1.37E-04
symbiont process (GO:0044403)	664	11	5.62	1.38E-04
regulation of protein transport (GO:0051223)	664	11	5.62	1.38E-04
response to antibiotic (GO:0046677)	309	8	8.79	1.43E-04
mononuclear cell proliferation (GO:0032943)	76	5	22.33	1.44E-04
regulation of protein localization (GO:0032880)	960	13	4.6	1.44E-04
salivary gland development (GO:0007431)	33	4	41.14	1.49E-04
positive regulation of vascular smooth muscle cell proliferation (GO:1904707)	33	4	41.14	1.49E-04
negative regulation of phosphate metabolic process (GO:0045936)	542	10	6.26	1.57E-04
negative regulation of phosphorus metabolic process (GO:0010563)	543	10	6.25	1.59E-04
regulation of pri-miRNA transcription by RNA polymerase II (GO:1902893)	34	4	39.93	1.64E-04
negative regulation of immune response (GO:0050777)	141	6	14.44	1.64E-04
regulation of stress fiber assembly (GO:0051492)	79	5	21.48	1.69E-04
negative regulation of apoptotic signaling pathway (GO:2001234)	222	7	10.7	1.69E-04
immune response-regulating cell surface receptor signaling pathway (GO:0002768)	426	9	7.17	1.69E-04
regulation of myeloid cell differentiation (GO:0045637)	223	7	10.65	1.73E-04
tumor necrosis factor-mediated signaling pathway (GO:0033209)	80	5	21.21	1.78E-04
response to fluid shear stress (GO:0034405)	35	4	38.79	1.79E-04
negative regulation of transcription by RNA polymerase II (GO:0000122)	833	12	4.89	1.86E-04
regulation of epithelial to mesenchymal transition (GO:0010717)	82	5	20.69	1.97E-04
regulation of peptide transport (GO:0090087)	694	11	5.38	1.99E-04
response to peptide (GO:1901652)	437	9	6.99	2.01E-04

peptidyl-amino acid modification (GO:0018193)	845	12	4.82	2.12E-04
positive regulation of smooth muscle cell proliferation (GO:0048661)	84	5	20.2	2.18E-04
regulation of actin cytoskeleton organization (GO:0032956)	332	8	8.18	2.26E-04
toll-like receptor signaling pathway (GO:0002224)	85	5	19.96	2.29E-04
response to progesterone (GO:0032570)	38	4	35.72	2.36E-04
positive regulation of animal organ morphogenesis (GO:0110110)	86	5	19.73	2.41E-04
response to unfolded protein (GO:0006986)	153	6	13.31	2.44E-04
regulation of mononuclear cell migration (GO:0071675)	39	4	34.81	2.57E-04
regulation of catabolic process (GO:0009894)	863	12	4.72	2.57E-04
positive regulation of myeloid cell differentiation (GO:0045639)	88	5	19.28	2.65E-04
cellular response to vascular endothelial growth factor stimulus (GO:0035924)	40	4	33.94	2.81E-04
humoral immune response (GO:0006959)	345	8	7.87	2.90E-04
regulation of extrinsic apoptotic signaling pathway (GO:2001236)	161	6	12.65	3.17E-04
regulation of actin filament bundle assembly (GO:0032231)	93	5	18.25	3.36E-04
positive regulation of angiogenesis (GO:0045766)	163	6	12.49	3.36E-04
exocrine system development (GO:0035272)	43	4	31.57	3.58E-04
positive regulation of endopeptidase activity (GO:0010950)	166	6	12.27	3.69E-04
regulation of cellular catabolic process (GO:0031329)	747	11	5	3.71E-04
response to organonitrogen compound (GO:0010243)	899	12	4.53	3.72E-04
response to nutrient levels (GO:0031667)	478	9	6.39	3.82E-04
positive regulation of endothelial cell migration (GO:0010595)	96	5	17.68	3.83E-04
negative regulation of smooth muscle cell proliferation (GO:0048662)	45	4	30.17	4.18E-04
regulation of innate immune response (GO:0045088)	365	8	7.44	4.18E-04
positive regulation of adaptive immune response based on somatic recombination of immune receptors built from immunoglobulin superfamily domains (GO:0002824)	99	5	17.14	4.36E-04
gland morphogenesis (GO:0022612)	99	5	17.14	4.37E-04
regulation of interferon-gamma production (GO:0032649)	99	5	17.14	4.38E-04
response to topologically incorrect protein (GO:0035966)	172	6	11.84	4.38E-04
positive regulation of epithelial to mesenchymal transition (GO:0010718)	46	4	29.51	4.46E-04
lymphocyte chemotaxis (GO:0048247)	46	4	29.51	4.47E-04
regulation of cellular component biogenesis (GO:0044087)	923	12	4.41	4.71E-04

transcription by RNA polymerase II (GO:0006366)	770	11	4.85	4.76E-04
positive regulation of cytosolic calcium ion concentration (GO:0007204)	268	7	8.86	4.96E-04
regulation of actin filament-based process (GO:0032970)	375	8	7.24	4.96E-04
positive regulation of pathway-restricted SMAD protein phosphorylation (GO:0010862)	48	4	28.28	5.14E-04
positive regulation of adaptive immune response (GO:0002821)	103	5	16.48	5.14E-04
ventricular cardiac muscle tissue morphogenesis (GO:0055010)	48	4	28.28	5.15E-04
positive regulation of vasculature development (GO:1904018)	180	6	11.31	5.49E-04
response to interferon-gamma (GO:0034341)	181	6	11.25	5.65E-04
myeloid leukocyte differentiation (GO:0002573)	106	5	16.01	5.80E-04
regulation of proteolysis (GO:0030162)	789	11	4.73	5.82E-04
response to laminar fluid shear stress (GO:0034616)	15	3	67.88	5.88E-04
positive regulation of nitric-oxide synthase biosynthetic process (GO:0051770)	15	3	67.88	5.89E-04
positive regulation of peptidase activity (GO:0010952)	183	6	11.13	5.94E-04
positive regulation of epithelial cell proliferation (GO:0050679)	185	6	11.01	6.28E-04
positive regulation of stress fiber assembly (GO:0051496)	51	4	26.62	6.29E-04
positive regulation of cellular component biogenesis (GO:0044089)	515	9	5.93	6.42E-04
regulation of cytoskeleton organization (GO:0051493)	515	9	5.93	6.43E-04
cellular response to organic cyclic compound (GO:0071407)	518	9	5.9	6.69E-04
regulation of type I interferon production (GO:0032479)	110	5	15.43	6.73E-04
regulation of leukocyte mediated immunity (GO:0002703)	189	6	10.77	6.95E-04
Fc receptor signaling pathway (GO:0038093)	189	6	10.77	6.96E-04
positive regulation of chemokine production (GO:0032722)	53	4	25.61	7.14E-04
ventricular cardiac muscle tissue development (GO:0003229)	54	4	25.14	7.62E-04
positive regulation of blood vessel endothelial cell migration (GO:0043536)	54	4	25.14	7.64E-04
cellular response to antibiotic (GO:0071236)	114	5	14.89	7.83E-04
regulation of immunoglobulin secretion (GO:0051023)	17	3	59.89	7.92E-04
response to inorganic substance (GO:0010035)	532	9	5.74	8.06E-04
regulation of cardiac muscle hypertrophy (GO:0010611)	55	4	24.68	8.08E-04
negative regulation of immune effector process (GO:0002698)	116	5	14.63	8.42E-04
muscle organ development (GO:0007517)	297	7	8	8.87E-04
regulation of transmembrane transport (GO:0034762)	540	9	5.66	8.97E-04

gland development (GO:0048732)	413	8	6.57	9.10E-04
regulation of vascular endothelial cell proliferation (GO:1905562)	18	3	56.56	9.11E-04
regulation of muscle hypertrophy (GO:0014743)	57	4	23.82	9.13E-04
positive regulation of immune effector process (GO:0002699)	201	6	10.13	9.43E-04
positive regulation of T cell activation (GO:0050870)	201	6	10.13	9.44E-04
Fc-epsilon receptor signaling pathway (GO:0038095)	121	5	14.02	1.00E-03
regulation of nitric-oxide synthase biosynthetic process (GO:0051769)	19	3	53.59	1.03E-03
regulation of T cell activation (GO:0050863)	305	7	7.79	1.03E-03
regulation of protein kinase B signaling (GO:0051896)	205	6	9.93	1.04E-03
lymphocyte migration (GO:0072676)	60	4	22.63	1.08E-03
regulation of cytosolic calcium ion concentration (GO:0051480)	308	7	7.71	1.08E-03
SMAD protein signal transduction (GO:0060395)	61	4	22.25	1.15E-03
cellular response to reactive oxygen species (GO:0034614)	125	5	13.58	1.15E-03
cardiac ventricle development (GO:0003231)	125	5	13.58	1.15E-03
response to muramyl dipeptide (GO:0032495)	20	3	50.91	1.17E-03
positive regulation of inflammatory response (GO:0050729)	126	5	13.47	1.19E-03
positive regulation of actin filament bundle assembly (GO:0032233)	62	4	21.9	1.21E-03
adenylate cyclase-activating G-protein coupled receptor signaling pathway (GO:0007189)	127	5	13.36	1.22E-03
cardiac chamber morphogenesis (GO:0003206)	128	5	13.26	1.27E-03
negative regulation of protein modification process (GO:0031400)	569	9	5.37	1.28E-03
regulation of macrophage differentiation (GO:0045649)	21	3	48.48	1.32E-03
leukocyte differentiation (GO:0002521)	319	7	7.45	1.32E-03
positive regulation of type I interferon production (GO:0032481)	64	4	21.21	1.34E-03
regulation of protein modification by small protein conjugation or removal (GO:1903320)	219	6	9.3	1.43E-03
positive regulation of DNA metabolic process (GO:0051054)	219	6	9.3	1.44E-03
positive regulation of interferon-alpha production (GO:0032727)	22	3	46.28	1.47E-03
regulation of SMAD protein signal transduction (GO:0060390)	22	3	46.28	1.48E-03
positive regulation of mononuclear cell migration (GO:0071677)	22	3	46.28	1.48E-03
hematopoietic or lymphoid organ development (GO:0048534)	585	9	5.22	1.55E-03

leukocyte activation (GO:0045321)	892	11	4.19	1.58E-03
viral process (GO:0016032)	589	9	5.19	1.63E-03
positive regulation of NIK/NF-kappaB signaling (GO:1901224)	68	4	19.96	1.64E-03
peptidyl-threonine phosphorylation (GO:0018107)	68	4	19.96	1.65E-03
platelet activation (GO:0030168)	137	5	12.39	1.68E-03
regulation of muscle system process (GO:0090257)	228	6	8.93	1.75E-03
positive regulation of organelle organization (GO:0010638)	597	9	5.12	1.78E-03
posttranscriptional regulation of gene expression (GO:0010608)	461	8	5.89	1.80E-03
cardiac muscle tissue morphogenesis (GO:0055008)	70	4	19.39	1.81E-03
muscle structure development (GO:0061061)	463	8	5.86	1.85E-03
ERBB signaling pathway (GO:0038127)	71	4	19.12	1.90E-03
negative regulation of transforming growth factor beta receptor signaling pathway (GO:0030512)	71	4	19.12	1.90E-03
negative regulation of ossification (GO:0030279)	71	4	19.12	1.90E-03
outflow tract morphogenesis (GO:0003151)	71	4	19.12	1.90E-03
negative regulation of neuron apoptotic process (GO:0043524)	142	5	11.95	1.94E-03
regulation of chemokine production (GO:0032642)	73	4	18.6	2.09E-03
negative regulation of cellular response to transforming growth factor beta stimulus (GO:1903845)	73	4	18.6	2.09E-03
positive regulation of cysteine-type endopeptidase activity (GO:2001056)	145	5	11.7	2.12E-03
lymphocyte proliferation (GO:0046651)	74	4	18.35	2.18E-03
regulation of lymphocyte mediated immunity (GO:0002706)	146	5	11.62	2.18E-03
cAMP-mediated signaling (GO:0019933)	146	5	11.62	2.19E-03
negative regulation of catalytic activity (GO:0043086)	768	10	4.42	2.21E-03
peptidyl-threonine modification (GO:0018210)	75	4	18.1	2.27E-03
regulation of phagocytosis (GO:0050764)	75	4	18.1	2.27E-03
response to osmotic stress (GO:0006970)	75	4	18.1	2.28E-03
regulation of peptidyl-tyrosine phosphorylation (GO:0050730)	242	6	8.41	2.31E-03
G-protein coupled receptor signaling pathway, coupled to cyclic nucleotide second messenger (GO:0007187)	243	6	8.38	2.36E-03
regulation of tissue remodeling (GO:0034103)	76	4	17.86	2.37E-03

regulation of GTPase activity (GO:0043087)	483	8	5.62	2.38E-03
positive regulation of protein secretion (GO:0050714)	244	6	8.35	2.40E-03
nucleotide-binding oligomerization domain containing signaling pathway (GO:0070423)	27	3	37.71	2.41E-03
positive regulation of cardiocyte differentiation (GO:1905209)	27	3	37.71	2.41E-03
negative regulation of myoblast differentiation (GO:0045662)	27	3	37.71	2.42E-03
positive regulation of transport (GO:0051050)	943	11	3.96	2.42E-03
skeletal system development (GO:0001501)	486	8	5.59	2.46E-03
response to toxic substance (GO:0009636)	488	8	5.56	2.53E-03
neuron development (GO:0048666)	784	10	4.33	2.53E-03
regulation of phospholipid metabolic process (GO:1903725)	78	4	17.4	2.56E-03
regulation of body fluid levels (GO:0050878)	489	8	5.55	2.56E-03
negative regulation of cell migration (GO:0030336)	249	6	8.18	2.62E-03
regulation of interferon-alpha production (GO:0032647)	28	3	36.36	2.63E-03
nucleotide-binding domain, leucine rich repeat containing receptor signaling pathway (GO:0035872)	28	3	36.36	2.63E-03
defense response to other organism (GO:0098542)	492	8	5.52	2.64E-03
regulation of binding (GO:0051098)	365	7	6.51	2.73E-03
rhythmic process (GO:0048511)	252	6	8.08	2.78E-03
neuron differentiation (GO:0030182)	964	11	3.87	2.88E-03
regulation of epidermis development (GO:0045682)	81	4	16.76	2.90E-03
positive regulation of interleukin-6 production (GO:0032755)	81	4	16.76	2.90E-03
muscle tissue morphogenesis (GO:0060415)	81	4	16.76	2.91E-03
negative regulation of DNA-binding transcription factor activity (GO:0043433)	158	5	10.74	2.97E-03
positive regulation of ossification (GO:0045778)	82	4	16.56	3.02E-03
positive regulation of protein kinase B signaling (GO:0051897)	159	5	10.67	3.04E-03
cellular response to interferon-gamma (GO:0071346)	159	5	10.67	3.04E-03
regulation of cellular response to growth factor stimulus (GO:0090287)	258	6	7.89	3.11E-03
positive regulation of cell division (GO:0051781)	83	4	16.36	3.14E-03
cellular response to organonitrogen compound (GO:0071417)	506	8	5.37	3.14E-03
regulation of biomineral tissue development (GO:0070167)	84	4	16.16	3.25E-03
regulation of ion transport (GO:0043269)	657	9	4.65	3.32E-03

regulation of cell junction assembly (GO:1901888)	85	4	15.97	3.38E-03
positive regulation of production of molecular mediator of immune response (GO:0002702)	85	4	15.97	3.38E-03
negative regulation of cell motility (GO:2000146)	264	6	7.71	3.45E-03
positive regulation of peptide secretion (GO:0002793)	264	6	7.71	3.45E-03
regulation of muscle adaptation (GO:0043502)	86	4	15.79	3.50E-03
roof of mouth development (GO:0060021)	86	4	15.79	3.51E-03
cardiac chamber development (GO:0003205)	165	5	10.28	3.51E-03
cytoplasmic pattern recognition receptor signaling pathway (GO:0002753)	32	3	31.82	3.59E-03
extrinsic apoptotic signaling pathway via death domain receptors (GO:0008625)	32	3	31.82	3.60E-03
regulation of superoxide metabolic process (GO:0090322)	32	3	31.82	3.61E-03
negative regulation of blood vessel endothelial cell migration (GO:0043537)	32	3	31.82	3.61E-03
positive regulation of interferon-beta production (GO:0032728)	32	3	31.82	3.62E-03
muscle organ morphogenesis (GO:0048644)	88	4	15.43	3.77E-03
regulation of reactive oxygen species metabolic process (GO:2000377)	169	5	10.04	3.85E-03
cyclic-nucleotide-mediated signaling (GO:0019935)	169	5	10.04	3.85E-03
regulation of endopeptidase activity (GO:0052548)	390	7	6.09	3.85E-03
sensory organ development (GO:0007423)	525	8	5.17	3.86E-03
cell proliferation (GO:0008283)	679	9	4.5	4.11E-03
positive regulation of phosphatidylinositol 3-kinase activity (GO:0043552)	34	3	29.95	4.20E-03
hemopoiesis (GO:0030097)	538	8	5.05	4.49E-03
regulation of system process (GO:0044057)	538	8	5.05	4.50E-03
negative regulation of cell differentiation (GO:0045596)	688	9	4.44	4.50E-03
negative regulation of extrinsic apoptotic signaling pathway via death domain receptors (GO:1902042)	36	3	28.28	4.86E-03
positive regulation of bone mineralization (GO:0030501)	36	3	28.28	4.86E-03
cellular response to ketone (GO:1901655)	96	4	14.14	5.07E-03
tissue remodeling (GO:0048771)	96	4	14.14	5.07E-03
response to starvation (GO:0042594)	181	5	9.38	5.12E-03
positive regulation of GTPase activity (GO:0043547)	411	7	5.78	5.15E-03
positive regulation of lipid kinase activity (GO:0090218)	37	3	27.52	5.19E-03

regulation of circadian rhythm (GO:0042752)	97	4	14	5.23E-03
blood coagulation (GO:0007596)	289	6	7.05	5.30E-03
coagulation (GO:0050817)	291	6	7	5.48E-03
positive regulation of B cell mediated immunity (GO:0002714)	38	3	26.79	5.49E-03
positive regulation of immunoglobulin mediated immune response (GO:0002891)	38	3	26.79	5.50E-03
B cell proliferation (GO:0042100)	38	3	26.79	5.51E-03
regulation of peptidase activity (GO:0052547)	417	7	5.7	5.51E-03
negative regulation of interleukin-6 production (GO:0032715)	38	3	26.79	5.52E-03
negative regulation of toll-like receptor signaling pathway (GO:0034122)	38	3	26.79	5.53E-03
regulation of NIK/NF-kappaB signaling (GO:1901222)	99	4	13.71	5.53E-03
hemostasis (GO:0007599)	294	6	6.93	5.69E-03
response to reactive oxygen species (GO:0000302)	187	5	9.07	5.78E-03
cellular response to cadmium ion (GO:0071276)	39	3	26.11	5.86E-03
negative regulation of cellular component movement (GO:0051271)	298	6	6.83	6.08E-03
regulation of leukocyte chemotaxis (GO:0002688)	102	4	13.31	6.12E-03
positive regulation of transmembrane receptor protein serine/threonine kinase signaling pathway (GO:0090100)	102	4	13.31	6.13E-03
embryonic organ development (GO:0048568)	426	7	5.58	6.18E-03
regulation of peptidyl-threonine phosphorylation (GO:0010799)	40	3	25.45	6.22E-03
regulation of granulocyte chemotaxis (GO:0071622)	40	3	25.45	6.23E-03
positive regulation of DNA replication (GO:0045740)	40	3	25.45	6.24E-03
cellular response to peptide (GO:1901653)	300	6	6.79	6.24E-03
regulation of protein ubiquitination (GO:0031396)	192	5	8.84	6.41E-03
positive regulation of lymphocyte mediated immunity (GO:0002708)	104	4	13.05	6.51E-03
negative regulation of neuron death (GO:1901215)	193	5	8.79	6.53E-03
bone development (GO:0060348)	193	5	8.79	6.54E-03
osteoclast differentiation (GO:0030316)	41	3	24.83	6.58E-03
positive regulation of B cell proliferation (GO:0030890)	41	3	24.83	6.59E-03
response to testosterone (GO:0033574)	41	3	24.83	6.60E-03
response to radiation (GO:0009314)	432	7	5.5	6.60E-03
regulation of cell adhesion mediated by integrin (GO:0033628)	41	3	24.83	6.61E-03

regulation of transforming growth factor beta receptor signaling pathway (GO:0017015)	105	4	12.93	6.67E-03
cellular response to toxic substance (GO:0097237)	196	5	8.66	6.87E-03
positive regulation of establishment of protein localization (GO:1904951)	436	7	5.45	6.87E-03
bone remodeling (GO:0046849)	42	3	24.24	6.92E-03
regulation of cellular response to transforming growth factor beta stimulus (GO:1903844)	107	4	12.69	7.04E-03
cellular response to metal ion (GO:0071248)	198	5	8.57	7.14E-03
regulation of osteoblast differentiation (GO:0045667)	108	4	12.57	7.26E-03
ventricular septum morphogenesis (GO:0060412)	43	3	23.68	7.33E-03
embryo development (GO:0009790)	914	10	3.71	7.33E-03
negative regulation of locomotion (GO:0040013)	312	6	6.53	7.38E-03
homeostasis of number of cells (GO:0048872)	200	5	8.48	7.42E-03
regulation of dephosphorylation (GO:0035303)	201	5	8.44	7.58E-03
regulation of cell-matrix adhesion (GO:0001952)	110	4	12.34	7.70E-03
extracellular matrix organization (GO:0030198)	315	6	6.46	7.72E-03
positive regulation of biomineral tissue development (GO:0070169)	44	3	23.14	7.75E-03
response to hydrogen peroxide (GO:0042542)	111	4	12.23	7.92E-03
regulation of ion transmembrane transport (GO:0034765)	448	7	5.3	7.92E-03
negative regulation of transmembrane receptor protein serine/threonine kinase signaling pathway (GO:0090101)	112	4	12.12	8.03E-03
regulation of cardiocyte differentiation (GO:1905207)	45	3	22.63	8.04E-03
negative regulation of fat cell differentiation (GO:0045599)	45	3	22.63	8.05E-03
positive regulation of immunoglobulin production (GO:0002639)	45	3	22.63	8.06E-03
myeloid cell differentiation (GO:0030099)	206	5	8.24	8.20E-03
negative regulation of transmembrane transport (GO:0034763)	113	4	12.01	8.23E-03
bone morphogenesis (GO:0060349)	113	4	12.01	8.24E-03
adenylate cyclase-modulating G-protein coupled receptor signaling pathway (GO:0007188)	207	5	8.2	8.34E-03
heart valve morphogenesis (GO:0003179)	46	3	22.13	8.47E-03
embryonic cranial skeleton morphogenesis (GO:0048701)	46	3	22.13	8.48E-03
regulation of B cell activation (GO:0050864)	208	5	8.16	8.48E-03

negative regulation of transport (GO:0051051)	456	7	5.21	8.48E-03
positive regulation of cytokine-mediated signaling pathway (GO:0001961)	46	3	22.13	8.49E-03
cell junction organization (GO:0034330)	210	5	8.08	8.83E-03
cardiocyte differentiation (GO:0035051)	117	4	11.6	9.24E-03
second-messenger-mediated signaling (GO:0019932)	329	6	6.19	9.26E-03
negative regulation of endothelial cell migration (GO:0010596)	48	3	21.21	9.43E-03
regulation of phosphatidylinositol 3-kinase activity (GO:0043551)	48	3	21.21	9.44E-03
regulation of mRNA stability (GO:0043488)	118	4	11.5	9.47E-03
neuron migration (GO:0001764)	118	4	11.5	9.48E-03
cellular response to unfolded protein (GO:0034620)	119	4	11.41	9.62E-03
regulation of DNA binding (GO:0051101)	119	4	11.41	9.64E-03
positive regulation of phospholipid metabolic process (GO:1903727)	49	3	20.78	9.78E-03
regulation of interferon-beta production (GO:0032648)	49	3	20.78	9.79E-03
response to steroid hormone (GO:0048545)	334	6	6.1	9.80E-03
regulation of fat cell differentiation (GO:0045598)	120	4	11.31	9.87E-03
positive regulation of protein catabolic process (GO:0045732)	218	5	7.78	1.01E-02
positive regulation of cellular catabolic process (GO:0031331)	337	6	6.04	1.02E-02
epidermal growth factor receptor signaling pathway (GO:0007173)	51	3	19.96	1.08E-02
leukocyte cell-cell adhesion (GO:0007159)	51	3	19.96	1.08E-02
lymphocyte homeostasis (GO:0002260)	51	3	19.96	1.08E-02
interleukin-1-mediated signaling pathway (GO:0070498)	51	3	19.96	1.08E-02
cellular response to inorganic substance (GO:0071241)	222	5	7.64	1.08E-02
connective tissue development (GO:0061448)	222	5	7.64	1.08E-02
eye development (GO:0001654)	341	6	5.97	1.08E-02
visual system development (GO:0150063)	341	6	5.97	1.08E-02
positive regulation of leukocyte mediated immunity (GO:0002705)	124	4	10.95	1.09E-02
regulation of transmembrane receptor protein serine/threonine kinase signaling pathway (GO:0090092)	223	5	7.61	1.09E-02
digestive tract development (GO:0048565)	124	4	10.95	1.10E-02
positive regulation of phagocytosis (GO:0050766)	52	3	19.58	1.12E-02
positive regulation of response to cytokine stimulus (GO:0060760)	52	3	19.58	1.12E-02

regulation of wound healing (GO:0061041)	126	4	10.77	1.14E-02
sensory system development (GO:0048880)	346	6	5.89	1.14E-02
regulation of supramolecular fiber organization (GO:1902903)	346	6	5.89	1.14E-02
cellular response to oxidative stress (GO:0034599)	226	5	7.51	1.15E-02
regulation of immunoglobulin mediated immune response (GO:0002889)	53	3	19.21	1.17E-02
regulation of morphogenesis of a branching structure (GO:0060688)	53	3	19.21	1.17E-02
regulation of B cell mediated immunity (GO:0002712)	53	3	19.21	1.17E-02
heart valve development (GO:0003170)	53	3	19.21	1.17E-02
regulation of RNA stability (GO:0043487)	127	4	10.69	1.17E-02
platelet degranulation (GO:0002576)	128	4	10.61	1.20E-02
positive regulation of cysteine-type endopeptidase activity involved in apoptotic process (GO:0043280)	129	4	10.52	1.23E-02
negative regulation of cellular protein metabolic process (GO:0032269)	992	10	3.42	1.23E-02
negative regulation of intracellular signal transduction (GO:1902532)	494	7	4.81	1.26E-02
response to endoplasmic reticulum stress (GO:0034976)	232	5	7.31	1.27E-02
positive regulation of fibroblast proliferation (GO:0048146)	55	3	18.51	1.28E-02
regulation of morphogenesis of an epithelium (GO:1905330)	131	4	10.36	1.29E-02
regulation of cysteine-type endopeptidase activity (GO:2000116)	234	5	7.25	1.31E-02
regulation of lipid kinase activity (GO:0043550)	56	3	18.18	1.34E-02
extracellular structure organization (GO:0043062)	359	6	5.67	1.34E-02
regulation of extrinsic apoptotic signaling pathway via death domain receptors (GO:1902041)	57	3	17.86	1.40E-02
regulation of tumor necrosis factor-mediated signaling pathway (GO:0010803)	57	3	17.86	1.40E-02
lymphocyte activation (GO:0046649)	362	6	5.63	1.40E-02
regulation of peptidyl-serine phosphorylation (GO:0033135)	135	4	10.06	1.42E-02
digestive system development (GO:0055123)	135	4	10.06	1.42E-02
cell-cell junction organization (GO:0045216)	135	4	10.06	1.43E-02
regulation of focal adhesion assembly (GO:0051893)	58	3	17.55	1.44E-02
regulation of interleukin-1 beta production (GO:0032651)	58	3	17.55	1.44E-02
regulation of cell-substrate junction assembly (GO:0090109)	58	3	17.55	1.44E-02
cellular response to topologically incorrect protein (GO:0035967)	136	4	9.98	1.44E-02

skeletal system morphogenesis (GO:0048705)	240	5	7.07	1.44E-02
positive regulation of lipid metabolic process (GO:0045834)	137	4	9.91	1.47E-02
positive regulation of cellular protein catabolic process (GO:1903364)	137	4	9.91	1.47E-02
positive regulation of phospholipase activity (GO:0010518)	59	3	17.26	1.50E-02
Ras protein signal transduction (GO:0007265)	243	5	6.98	1.50E-02
positive regulation of secretion by cell (GO:1903532)	369	6	5.52	1.50E-02
artery morphogenesis (GO:0048844)	60	3	16.97	1.57E-02
negative regulation of cellular response to growth factor stimulus (GO:0090288)	140	4	9.7	1.58E-02
regulation of mRNA catabolic process (GO:0061013)	140	4	9.7	1.58E-02
regulation of multi-organism process (GO:0043900)	374	6	5.44	1.59E-02
response to peptide hormone (GO:0043434)	375	6	5.43	1.60E-02
regulation of B cell proliferation (GO:0030888)	61	3	16.69	1.62E-02
regulation of protein catabolic process (GO:0042176)	376	6	5.42	1.62E-02
cellular response to starvation (GO:0009267)	143	4	9.49	1.69E-02
regulation of smooth muscle cell migration (GO:0014910)	62	3	16.42	1.70E-02
kidney development (GO:0001822)	252	5	6.73	1.73E-02
cellular response to peptide hormone stimulus (GO:0071375)	252	5	6.73	1.73E-02
regulation of growth (GO:0040008)	690	8	3.93	1.75E-02
regulation of toll-like receptor signaling pathway (GO:0034121)	63	3	16.16	1.76E-02
regulation of adherens junction organization (GO:1903391)	63	3	16.16	1.76E-02
regulation of cartilage development (GO:0061035)	63	3	16.16	1.76E-02
positive regulation of protein complex assembly (GO:0031334)	254	5	6.68	1.78E-02
regulation of endocytosis (GO:0030100)	254	5	6.68	1.78E-02
negative regulation of mitotic cell cycle (GO:0045930)	255	5	6.65	1.80E-02
cellular response to hydrogen peroxide (GO:0070301)	64	3	15.91	1.82E-02
regulation of cellular amide metabolic process (GO:0034248)	386	6	5.28	1.82E-02
modification of morphology or physiology of other organism (GO:0035821)	147	4	9.24	1.83E-02
regulation of osteoclast differentiation (GO:0045670)	65	3	15.66	1.89E-02
response to cadmium ion (GO:0046686)	65	3	15.66	1.89E-02
negative regulation of epithelial cell migration (GO:0010633)	65	3	15.66	1.89E-02
mesenchymal cell differentiation (GO:0048762)	149	4	9.11	1.91E-02

regulation of response to wounding (GO:1903034)	150	4	9.05	1.95E-02
negative regulation of protein phosphorylation (GO:0001933)	392	6	5.19	1.95E-02
epithelial to mesenchymal transition (GO:0001837)	66	3	15.43	1.96E-02
antimicrobial humoral immune response mediated by antimicrobial peptide (GO:0061844)	66	3	15.43	1.96E-02
positive regulation of macrophage migration (GO:1905523)	15	2	45.25	1.99E-02
regulation of actin filament organization (GO:0110053)	262	5	6.48	1.99E-02
aging (GO:0007568)	262	5	6.48	1.99E-02
leukocyte homeostasis (GO:0001776)	67	3	15.2	2.03E-02
cell cycle arrest (GO:0007050)	152	4	8.93	2.03E-02
regulation of bone mineralization (GO:0030500)	68	3	14.97	2.11E-02
regulation of acute inflammatory response (GO:0002673)	154	4	8.82	2.12E-02
positive regulation of secretion (GO:0051047)	400	6	5.09	2.13E-02
positive regulation of protein transport (GO:0051222)	401	6	5.08	2.16E-02
macrophage migration (GO:1905517)	16	2	42.42	2.21E-02
ventricular trabecula myocardium morphogenesis (GO:0003222)	16	2	42.42	2.21E-02
adaptive immune response based on somatic recombination of immune receptors built from immunoglobulin superfamily domains (GO:0002460)	269	5	6.31	2.21E-02
renal system development (GO:0072001)	270	5	6.28	2.24E-02
embryonic morphogenesis (GO:0048598)	556	7	4.27	2.24E-02
regulation of phospholipase activity (GO:0010517)	70	3	14.55	2.25E-02
ventricular septum development (GO:0003281)	70	3	14.55	2.25E-02
regulation of cell division (GO:0051302)	157	4	8.65	2.25E-02
regulation of T cell proliferation (GO:0042129)	157	4	8.65	2.25E-02
protein deubiquitination (GO:0016579)	271	5	6.26	2.25E-02
positive regulation of catabolic process (GO:0009896)	405	6	5.03	2.25E-02
positive regulation of lipase activity (GO:0060193)	70	3	14.55	2.26E-02
regulation of DNA metabolic process (GO:0051052)	406	6	5.02	2.26E-02
regulation of cell development (GO:0060284)	903	9	3.38	2.26E-02
cell adhesion (GO:0007155)	906	9	3.37	2.31E-02
negative regulation of ATPase activity (GO:0032780)	17	2	39.93	2.42E-02

regulation of interferon-gamma biosynthetic process (GO:0045072)	17	2	39.93	2.42E-02
lymphocyte apoptotic process (GO:0070227)	17	2	39.93	2.42E-02
hair follicle development (GO:0001942)	72	3	14.14	2.42E-02
biological adhesion (GO:0022610)	912	9	3.35	2.42E-02
positive regulation of multi-organism process (GO:0043902)	162	4	8.38	2.48E-02
regulation of membrane potential (GO:0042391)	416	6	4.9	2.52E-02
regulation of lymphocyte differentiation (GO:0045619)	163	4	8.33	2.53E-02
negative regulation of catabolic process (GO:0009895)	279	5	6.08	2.53E-02
regulation of interleukin-1 production (GO:0032652)	74	3	13.76	2.58E-02
skin epidermis development (GO:0098773)	74	3	13.76	2.58E-02
cardiac septum morphogenesis (GO:0060411)	74	3	13.76	2.59E-02
membrane protein intracellular domain proteolysis (GO:0031293)	18	2	37.71	2.64E-02
ventricular cardiac muscle cell differentiation (GO:0055012)	18	2	37.71	2.64E-02
monocyte differentiation (GO:0030224)	18	2	37.71	2.64E-02
craniofacial suture morphogenesis (GO:0097094)	18	2	37.71	2.64E-02
molting cycle process (GO:0022404)	75	3	13.58	2.64E-02
positive regulation of cell adhesion mediated by integrin (GO:0033630)	18	2	37.71	2.65E-02
mononuclear cell differentiation (GO:1903131)	18	2	37.71	2.65E-02
negative regulation of vascular smooth muscle cell proliferation (GO:1904706)	18	2	37.71	2.65E-02
regulation of cellular response to oxidative stress (GO:1900407)	75	3	13.58	2.65E-02
regulation of cardiac muscle tissue development (GO:0055024)	75	3	13.58	2.65E-02
hair cycle process (GO:0022405)	75	3	13.58	2.65E-02
protein modification by small protein removal (GO:0070646)	287	5	5.91	2.80E-02
positive regulation of B cell activation (GO:0050871)	169	4	8.03	2.82E-02
negative regulation of cation transmembrane transport (GO:1904063)	77	3	13.22	2.83E-02
response to muscle stretch (GO:0035994)	19	2	35.72	2.85E-02
positive regulation of cellular response to oxidative stress (GO:1900409)	19	2	35.72	2.85E-02
leukocyte activation involved in inflammatory response (GO:0002269)	19	2	35.72	2.86E-02
microglial cell activation (GO:0001774)	19	2	35.72	2.86E-02
regulation of hair follicle development (GO:0051797)	19	2	35.72	2.86E-02
response to salt (GO:1902074)	19	2	35.72	2.86E-02

regulation of monocyte differentiation (GO:0045655)	19	2	35.72	2.87E-02
positive regulation of cardiac muscle cell differentiation (GO:2000727)	19	2	35.72	2.87E-02
positive regulation of apoptotic signaling pathway (GO:2001235)	170	4	7.99	2.87E-02
intracellular receptor signaling pathway (GO:0030522)	170	4	7.99	2.87E-02
regulation of phosphatase activity (GO:0010921)	170	4	7.99	2.87E-02
negative regulation of phosphorylation (GO:0042326)	430	6	4.74	2.88E-02
regulation of translational initiation (GO:0006446)	78	3	13.05	2.89E-02
regulation of hemostasis (GO:1900046)	78	3	13.05	2.89E-02
regulation of blood coagulation (GO:0030193)	78	3	13.05	2.89E-02
positive regulation of leukocyte chemotaxis (GO:0002690)	79	3	12.89	2.98E-02
cell cycle process (GO:0022402)	947	9	3.23	2.98E-02
negative regulation of hydrolase activity (GO:0051346)	434	6	4.69	2.99E-02
positive regulation of T-helper 1 type immune response (GO:0002827)	20	2	33.94	3.08E-02
negative regulation of leukocyte proliferation (GO:0070664)	80	3	12.73	3.08E-02
regulation of DNA-templated transcription in response to stress (GO:0043620)	80	3	12.73	3.08E-02
ephrin receptor signaling pathway (GO:0048013)	80	3	12.73	3.08E-02
regulation of bicellular tight junction assembly (GO:2000810)	20	2	33.94	3.09E-02
vascular endothelial growth factor signaling pathway (GO:0038084)	20	2	33.94	3.09E-02
regulation of tumor necrosis factor biosynthetic process (GO:0042534)	20	2	33.94	3.09E-02
camera-type eye development (GO:0043010)	296	5	5.73	3.10E-02
artery development (GO:0060840)	81	3	12.57	3.16E-02
negative regulation of protein modification by small protein conjugation or removal (GO:1903321)	81	3	12.57	3.16E-02
negative regulation of multi-organism process (GO:0043901)	176	4	7.71	3.16E-02
chondrocyte differentiation (GO:0002062)	82	3	12.42	3.26E-02
regulation of coagulation (GO:0050818)	82	3	12.42	3.26E-02
defense response to bacterium (GO:0042742)	301	5	5.64	3.31E-02
leukocyte apoptotic process (GO:0071887)	21	2	32.32	3.34E-02
negative regulation of cell junction assembly (GO:1901889)	21	2	32.32	3.34E-02
regulation of lymphocyte chemotaxis (GO:1901623)	21	2	32.32	3.34E-02
protein autophosphorylation (GO:0046777)	180	4	7.54	3.39E-02

regulation of cytokine secretion (GO:0050707)	180	4	7.54	3.40E-02
urogenital system development (GO:0001655)	304	5	5.58	3.43E-02
regulation of response to oxidative stress (GO:1902882)	84	3	12.12	3.46E-02
positive regulation of peptidyl-tyrosine phosphorylation (GO:0050731)	182	4	7.46	3.51E-02
tissue homeostasis (GO:0001894)	182	4	7.46	3.52E-02
regulation of fibroblast proliferation (GO:0048145)	85	3	11.98	3.56E-02
positive regulation of response to oxidative stress (GO:1902884)	22	2	30.85	3.59E-02
positive regulation of toll-like receptor signaling pathway (GO:0034123)	22	2	30.85	3.60E-02
response to cholesterol (GO:0070723)	22	2	30.85	3.60E-02
regulation of macrophage chemotaxis (GO:0010758)	22	2	30.85	3.60E-02
metal ion transport (GO:0030001)	615	7	3.86	3.60E-02
negative regulation of cell activation (GO:0050866)	184	4	7.38	3.63E-02
small GTPase mediated signal transduction (GO:0007264)	309	5	5.49	3.64E-02
positive regulation of muscle cell differentiation (GO:0051149)	87	3	11.7	3.76E-02
defense response to virus (GO:0051607)	186	4	7.3	3.76E-02
inner ear development (GO:0048839)	186	4	7.3	3.76E-02
regulation of protein complex assembly (GO:0043254)	459	6	4.44	3.81E-02
peptidyl-tyrosine phosphorylation (GO:0018108)	187	4	7.26	3.82E-02
secondary palate development (GO:0062009)	23	2	29.51	3.85E-02
branching involved in mammary gland duct morphogenesis (GO:0060444)	23	2	29.51	3.86E-02
positive regulation of myoblast differentiation (GO:0045663)	23	2	29.51	3.86E-02
cardiac muscle cell differentiation (GO:0055007)	89	3	11.44	3.97E-02
peptidyl-tyrosine modification (GO:0018212)	190	4	7.14	4.02E-02
response to vitamin (GO:0033273)	90	3	11.31	4.09E-02
negative regulation of ion transmembrane transport (GO:0034766)	90	3	11.31	4.09E-02
regulation of cellular extravasation (GO:0002691)	24	2	28.28	4.11E-02
TRIF-dependent toll-like receptor signaling pathway (GO:0035666)	24	2	28.28	4.12E-02
protein import into nucleus, translocation (GO:0000060)	24	2	28.28	4.12E-02
negative regulation of viral transcription (GO:0032897)	24	2	28.28	4.13E-02
response to salt stress (GO:0009651)	24	2	28.28	4.13E-02
negative regulation of biomineral tissue development (GO:0070168)	24	2	28.28	4.13E-02

regulation of homotypic cell-cell adhesion (GO:0034110)	24	2	28.28	4.14E-02
positive regulation of osteoclast differentiation (GO:0045672)	24	2	28.28	4.14E-02
hyperosmotic response (GO:0006972)	24	2	28.28	4.15E-02
regulation of organ growth (GO:0046620)	91	3	11.19	4.16E-02
positive regulation of endothelial cell proliferation (GO:0001938)	91	3	11.19	4.17E-02
positive regulation of reactive oxygen species metabolic process (GO:2000379)	91	3	11.19	4.17E-02
actin cytoskeleton organization (GO:0030036)	471	6	4.32	4.23E-02
molting cycle (GO:0042303)	92	3	11.07	4.27E-02
activation of cysteine-type endopeptidase activity involved in apoptotic process (GO:0006919)	92	3	11.07	4.28E-02
hair cycle (GO:0042633)	92	3	11.07	4.28E-02
positive regulation of supramolecular fiber organization (GO:1902905)	195	4	6.96	4.32E-02
B cell homeostasis (GO:0001782)	25	2	27.15	4.39E-02
positive regulation of muscle cell apoptotic process (GO:0010661)	25	2	27.15	4.39E-02
negative regulation of viral process (GO:0048525)	94	3	10.83	4.49E-02
regulation of lipase activity (GO:0060191)	94	3	10.83	4.50E-02
cellular response to calcium ion (GO:0071277)	94	3	10.83	4.50E-02
positive regulation of ubiquitin-dependent protein catabolic process (GO:2000060)	94	3	10.83	4.50E-02
cell-cell adhesion (GO:0098609)	481	6	4.23	4.63E-02
neuroinflammatory response (GO:0150076)	26	2	26.11	4.64E-02
MyD88-independent toll-like receptor signaling pathway (GO:0002756)	26	2	26.11	4.64E-02
replacement ossification (GO:0036075)	26	2	26.11	4.65E-02
cardiac muscle tissue growth (GO:0055017)	26	2	26.11	4.65E-02
regulation of lipopolysaccharide-mediated signaling pathway (GO:0031664)	26	2	26.11	4.66E-02
response to sterol (GO:0036314)	26	2	26.11	4.66E-02
glial cell activation (GO:0061900)	26	2	26.11	4.66E-02
positive regulation of collagen biosynthetic process (GO:0032967)	26	2	26.11	4.67E-02
pharyngeal system development (GO:0060037)	26	2	26.11	4.67E-02
endochondral ossification (GO:0001958)	26	2	26.11	4.68E-02
positive regulation of plasma membrane bounded cell projection assembly (GO:0120034)	96	3	10.61	4.70E-02

regulation of cell-substrate adhesion (GO:0010810)	202	4	6.72	4.79E-02
embryonic skeletal system morphogenesis (GO:0048704)	97	3	10.5	4.82E-02
regulation of T-helper 1 type immune response (GO:0002825)	27	2	25.14	4.88E-02
positive regulation of isotype switching (GO:0045830)	27	2	25.14	4.88E-02
positive regulation of T cell proliferation (GO:0042102)	98	3	10.39	4.88E-02
positive regulation of collagen metabolic process (GO:0010714)	27	2	25.14	4.89E-02
epithelial cell apoptotic process (GO:1904019)	27	2	25.14	4.89E-02
positive regulation of peptidyl-threonine phosphorylation (GO:0010800)	27	2	25.14	4.90E-02
negative regulation of epithelial to mesenchymal transition (GO:0010719)	27	2	25.14	4.90E-02
regulation of translation (GO:0006417)	337	5	5.04	4.90E-02
regulation of macrophage migration (GO:1905521)	27	2	25.14	4.91E-02
regulation of hair cycle (GO:0042634)	27	2	25.14	4.91E-02
regulation of odontogenesis (GO:0042481)	27	2	25.14	4.91E-02
response to angiotensin (GO:1990776)	27	2	25.14	4.92E-02
modulation by host of viral transcription (GO:0043921)	27	2	25.14	4.92E-02
viral genome replication (GO:0019079)	27	2	25.14	4.93E-02
modulation by host of symbiont transcription (GO:0052472)	27	2	25.14	4.93E-02
cardiac epithelial to mesenchymal transition (GO:0060317)	27	2	25.14	4.94E-02
positive regulation of neutrophil migration (GO:1902624)	27	2	25.14	4.94E-02
positive regulation of acute inflammatory response (GO:0002675)	27	2	25.14	4.95E-02
heart growth (GO:0060419)	27	2	25.14	4.95E-02
